# The Unified Medical Language System at 30 Years and How It Is Used and Published: Systematic Review and Content Analysis

**DOI:** 10.2196/20675

**Published:** 2021-08-27

**Authors:** Xia Jing

**Affiliations:** 1 Department of Public Health Sciences College of Behavioral, Social and Health Sciences Clemson University Clemson, SC United States

**Keywords:** Unified Medical Language System, systematic literature analysis, biomedical informatics, health informatics

## Abstract

**Background:**

The Unified Medical Language System (UMLS) has been a critical tool in biomedical and health informatics, and the year 2021 marks its 30th anniversary. The UMLS brings together many broadly used vocabularies and standards in the biomedical field to facilitate interoperability among different computer systems and applications.

**Objective:**

Despite its longevity, there is no comprehensive publication analysis of the use of the UMLS. Thus, this review and analysis is conducted to provide an overview of the UMLS and its use in English-language peer-reviewed publications, with the objective of providing a comprehensive understanding of how the UMLS has been used in English-language peer-reviewed publications over the last 30 years.

**Methods:**

PubMed, ACM Digital Library, and the Nursing & Allied Health Database were used to search for studies. The primary search strategy was as follows: UMLS was used as a Medical Subject Headings term or a keyword or appeared in the title or abstract. Only English-language publications were considered. The publications were screened first, then coded and categorized iteratively, following the grounded theory. The review process followed the PRISMA (Preferred Reporting Items for Systematic Reviews and Meta-Analyses) guidelines.

**Results:**

A total of 943 publications were included in the final analysis. Moreover, 32 publications were categorized into 2 categories; hence the total number of publications before duplicates are removed is 975. After analysis and categorization of the publications, UMLS was found to be used in the following emerging themes or areas (the number of publications and their respective percentages are given in parentheses): natural language processing (230/975, 23.6%), information retrieval (125/975, 12.8%), terminology study (90/975, 9.2%), ontology and modeling (80/975, 8.2%), medical subdomains (76/975, 7.8%), other language studies (53/975, 5.4%), artificial intelligence tools and applications (46/975, 4.7%), patient care (35/975, 3.6%), data mining and knowledge discovery (25/975, 2.6%), medical education (20/975, 2.1%), degree-related theses (13/975, 1.3%), digital library (5/975, 0.5%), and the UMLS itself (150/975, 15.4%), as well as the UMLS for other purposes (27/975, 2.8%).

**Conclusions:**

The UMLS has been used successfully in patient care, medical education, digital libraries, and software development, as originally planned, as well as in degree-related theses, the building of artificial intelligence tools, data mining and knowledge discovery, foundational work in methodology, and middle layers that may lead to advanced products. Natural language processing, the UMLS itself, and information retrieval are the 3 most common themes that emerged among the included publications. The results, although largely related to academia, demonstrate that UMLS achieves its intended uses successfully, in addition to achieving uses broadly beyond its original intentions.

## Introduction

### Background

The Unified Medical Language System (UMLS) [1] is a critical resource in biomedical and health informatics. It was created and released by the National Library of Medicine, an institute of the National Institutes of Health (NIH). The first edition of UMLS Knowledge Sources was distributed in 1991 [1], although its conceptualization can be traced to 1986 [2]. Currently, there are three UMLS Knowledge Sources: Metathesaurus, Semantic Network, and SPECIALIST Lexicon and Lexical Tools. The Metathesaurus contains approximately 4.4 million concepts and 16 million unique concept names, which are from 218 source vocabularies in 25 languages worldwide (2021AA release). The Semantic Network provides consistent categorization for all concepts included in UMLS [3]. The SPECIALIST Lexicon and Lexical Tools provide large syntactic lexicon tools that have been used broadly in the biomedical and health fields to normalize strings and lexical variants.

UMLS brings together many broadly used vocabularies and standards in the biomedical field to facilitate interoperability and semantic understanding among different computer systems and software applications [4,5]. UMLS has been maintained and further developed by the National Library of Medicine over the past 30 years. In the initial publication, UMLS was intended to be used in four main areas: patient care, medical education, library service, and product development [1]. A comprehensive evaluation of the UMLS would be a large project; however, a close examination of the literature in the form of peer-reviewed publications can provide a perspective on how the UMLS is used in academia, which is the *rationale* for this literature review.

### Objective

The year 2021 is the 30th anniversary of UMLS. Despite its longevity, there is no comprehensive publication analysis of UMLS. To call attention to the importance of UMLS and highlight its critical role in advancing biomedical informatics, health informatics, medicine, and health care, this systematic analysis was conducted to demonstrate how UMLS has been used, based on peer-reviewed publications in English over the past 30 years, which is the objective of this literature review.

## Methods

### Literature Search Sources and Strategies

#### Overview

PubMed, ACM Digital Library, and the Nursing & Allied Health Database were used for the search. The primary strategy was to search literature that either used UMLS as a MeSH (Medical Subject Headings) term or a keyword or had UMLS or *unified medical language system* in the title or abstract.

#### Search Strategy in PubMed on April 28, 2020

*unified medical language system* [MeSH term]

#### Search Strategy in ACM Digital Library on April 28, 2020

Searches were conducted within the ACM Guide to Computing Literature:

[Publication title: umls] OR [Publication title: unified medical language system*] OR [Abstract: umls] OR [Abstract: unified medical language system*]

The following journals were excluded because they are indexed in PubMed: *Journal of Biomedical Informatics, Artificial Intelligence in Medicine,* and *Bioinformatics*.

#### Search Strategy in the Nursing & Allied Health Database on April 28, 2020

Searches were conducted within peer-reviewed publications:

mesh (*unified medical language system*) OR ti(umls) OR ti(*unified medical language system*) OR ab(umls) OR ab (*unified medical language system*)

### Literature Examination

#### Literature Examination Process

The literature examination process followed the grounded theory. The steps for the content analysis were as follows: all duplicate publications were removed before the literature examination. The exclusion criteria included the following: UMLS not mentioned in the abstract, abstract unavailable, or non-English publications.

The first step of the content analysis was to go over and then code (or index) each title and to record the repeated themes or topics. The second step was to go over each abstract one by one to code (or index) each abstract again, record the repeated themes or topics, and exclude the irrelevant publications. The third step was to organize the themes and group them according to their similarities. Subsequently, each publication was classified into the corresponding theme, and additional themes were created during the process.

The classification step was conducted iteratively. The first round began with obvious and repeated themes. Each publication was examined and, as appropriate, categorized by theme. I began with the relatively obvious themes, each of which had relatively fewer publications. The initial group of themes included artificial intelligence (AI) tools and applications, other language UMLS studies, medical education, patient care, medical subdomains, digital library, and degree-related theses. The publications were then classified, one by one, for the following themes: UMLS itself, information retrieval, terminology study, natural language processing (NLP), ontology and modeling, data mining, and knowledge discovery. The publications that fell outside of these themes during the coding (or indexing) process were classified last. The classification process stopped when all publications were classified into themes without the need for additional consideration. The themes were adjusted whenever needed during the iterative classification processes. The publications were then analyzed, categorized, synthesized iteratively, counted, and recorded into each category.

A word cloud picture (Multimedia Appendix 1) based on the titles included in this comprehensive literature analysis was generated by removing all commonly used words. The Pro Word Cloud function within Microsoft Word (Microsoft Corporation) was used to generate the word cloud picture.

#### Literature Classification Principles

The following principles were followed during classification: the primary principle is that when a publication is analyzed, the objectives of the publication, not the methods implemented, are the prioritized reasons for the categorization. The secondary principle is to maximize the possibility that a publication will stand out among the publications in each category; that is, if a publication has an approximately equal possibility to be classified into 2 categories, the one with fewer publications wins. The third principle is to give publications on applications and patient care a higher priority than methodology development or foundational studies, in general. The fourth principle is to classify a publication into the most specific category whenever possible. The rationale for following these principles is based on the literature review. Instead of providing a comprehensive evaluation of all aspects of the UMLS, I attempted to determine how the UMLS is used in the real world. I focused on its application as a critical factor. As the UMLS is found in medicine, patient care is a higher priority.

In addition, I used this opportunity to recognize my peers’ contributions by maximizing the possibility of their publications standing out because only a small fraction of the work can be awarded a prize. These principles helped me to classify all the publications in a more consistent, clear, reproducible, and objective manner.

#### Literature Review Guideline

The systematic literature analysis protocol has not been registered. The data items used in this literature review including title, author, publication year, journal or conference proceeding, abstract, MeSH terms or keywords, PubMed ID (if available), full-text for some publications, and what was UMLS used for. The PRISMA (Preferred Reporting Items for Systematic Reviews and Meta-Analyses) guidelines [6] were followed and most of the checklist items were included. The PRISMA checklist is provided in Multimedia Appendix 2.

## Results

### Overview

**Figure 1:**
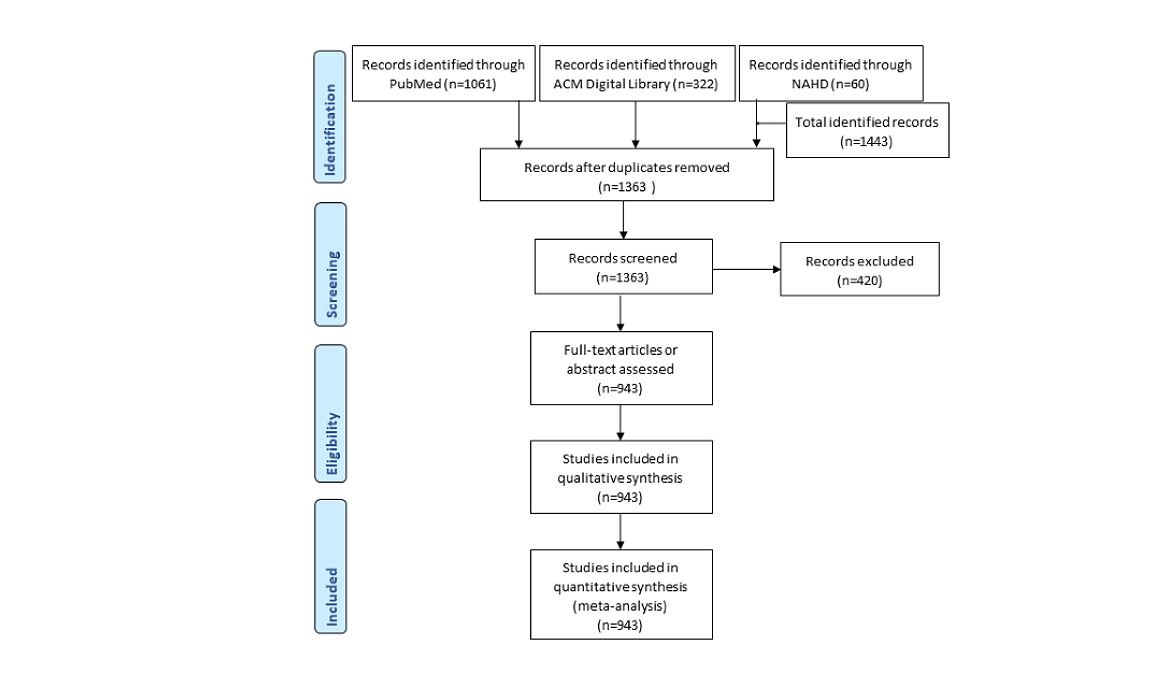
Flowchart of the literature search, screening, analysis, and its records. NAHD: Nursing & Allied Health Database.

The search strategies yielded 1061 records in PubMed, 322 in the ACM Digital Library, and 60 in the Nursing & Allied Health Database. After removing the duplicates, records without abstracts, non-English records, and abstracts that did not mention UMLS, 943 records were retained for the final analysis. Figure 1 [6] shows detailed records of the literature search, screening, and analysis.

Multimedia Appendix 3 shows the yearly number of the included UMLS publications over the last 30 years. Table 1 presents the themes that emerged and the corresponding number of publications for each category. This table provides an overview of the results of the systematic analysis. Multimedia Appendix 4 presents the major themes, topics, and corresponding publication counts.

**Table 1:** Results of the Unified Medical Language System systematic literature analysis: emerging themes, subtopics, and the number of publications in each category before and after removing duplicates.

Themes and subtopics					Publication counts (n=975), n (%)			After removing the duplicates (n=943), n (%)
**Artificial intelligence tools and applications**					46 (4.7)			46 (4.9)
	Automatic annotation or interpretation			7 (15.2)			7 (15.2)	
	Automatic coding			7 (15.2)			7 (15.2)	
	Automatic summarization			15 (32.6)			15 (32.6)	
	Question-answering systems			10 (21.7)			10 (21.7)	
	Other intelligent tools			7 (15.2)			7 (15.2)	
Data mining and knowledge discovery					25 (2.6)			25 (2.7)
Degree-related theses					13 (1.3)			8 (0.8)
Digital library					5 (0.5)			4 (0.4)
**Information retrieval**					125 (12.8)			119 (12.6)
	Image retrieval			20 (16)			20 (16.8)	
	Indexing			33(26.4)			30 (25.2)	
	Information retrieval			34 (27.2)			32 (26.9)	
	Information retrieval system and search engine			8 (6.4)			8 (6.7)	
	Performance			13 (10.4)			12 (10.1)	
	Query			17 (13.6)			17 (14.3)	
Medical education					20 (2.1)			19 (2)
Medical subdomains (34 subdomains)					76 (7.8)			76 (8.1)
**NLP^a^**					230 (23.6)			230 (24.4)
	Abbreviation			11 (4.8)			11 (4.8)	
	Feature identification or extraction or phenotyping			4 (1.7)			4 (1.7)	
	Lexicon and/or inventory			7 (3)			7 (3)	
	**Semantic**			165 (71.7)			165 (71.7)	
		Concept recognition or extraction	42 (25.5)			42 (25.5)		
		Name entity recognition or extraction	18 (10.9)			18 (10.9)		
		Natural language, vocabulary, question generation	3 (1.8)			3 (1.8)		
		Natural language understanding	3 (1.8)			3 (1.8)		
		Relationship recognition or extraction	45 (27.3)			45 (27.3)		
		Semantic similarity, relatedness, or distance	20 (12.1)			20 (12.1)		
		Word sense disambiguation	34 (20.6)			34 (20.6)		
	**Syntax**			18 (7.8)			18 (7.8)	
		Parsing	5 (27.8)			5 (27.8)		
		Tagging	5 (27.8)			5 (27.8)		
		Terminology extraction	8 (44.4)			8 (44.4)		
	Text classification			10 (4.4)			10 (4.4)	
	Other NLP-related publications			15 (6.5)			15 (6.5)	
**Ontology and modeling**					80 (8.2)			79 (8.4)
	Classification or taxonomy			21 (26.3)			21 (26.6)	
	Modeling			18 (22.5)			17 (21.5)	
	Ontology			29 (36.3)			29 (36.7)	
	Representation			12 (15)			12 (15.2)	
Other languages (10 languages)					53 (5.4)			47 (5)
Patient care					35 (3.6)			27 (2.9)
**Terminology study**					90 (9.2)			90 (9.5)
	Comparison of terminologies			6 (6.7)			60 (6.7)	
	Construction of terminology or taxonomy			19 (21.1)			19 (21.1)	
	Harmonization			46 (51.1)			46 (51.1)	
	Interoperability			7 (7.8)			7 (7.8)	
	Quality assurance			7 (7.8)			7 (7.8)	
	Other publications of terminology			5 (5.6)			5 (5.6)	
**UMLS^b^ itself**					150 (15.4)			146 (15.5)
	Applications or tools for UMLS			25 (16.7)			25 (17.1)	
	Auditing of UMLS			24 (16)			24 (16.4)	
	Components of UMLS or UMLS			78 (52)			76 (52.1)	
	Coverage of UMLS			23 (15.3)			21 (14.4)	
**UMLS for other purposes**					27 (2.8)			27 (2.9)
	Auditing			3 (11.1)			3 (11.1)	
	Consumer health			4 (14.8)			4 (14.8)	
	Integrated system or data			17 (63)			17 (63)	
	Other research use			3 (11.1)			3 (11.1)	

^a^NLP: natural language processing.

^b^UMLS: Unified Medical Language System.

### Themes, Subtopics, and Publications Under Each Category

After the included publications were examined carefully, the following themes emerged during analysis and synthesis.

#### UMLS Is Used in AI Tools and Applications

The UMLS has been used in developing AI tools and applications since 1994 [7] (publication; the actual work started many years ago). The AI tools include question-answering systems, automatic summarization, automatic coding, automatic annotation, and plagiarism detection. Question-answering systems focus on the medical domain. Some question-answering systems focus specifically on answering consumers’ questions. Automatic summarization focuses mainly on summarizing medical literature, textbooks, and patient records. This category also includes methodology exploration. Multimedia Appendix 5 includes the 46 UMLS publications in this category.

I recognize that there is an overlap between AI tools and NLP. The criterion used concerned whether a publication focused on the final products. If so, it was classified into the AI tools and applications category; if a publication focused on the middle-layer methodology to enhance performance, it was classified into the NLP category.

Automatic translation can also be categorized into this theme; however, the publications were categorized on automatic translation into the other language UMLS studies category, using a more detailed description. Similarly, intelligent tutoring systems were classified into medical education instead of AI tools and applications. These categories should be cross-referenced accordingly.

#### UMLS-Based Data Mining and Knowledge Discovery

UMLS is used broadly as a critical tool in data mining and knowledge discovery in the biomedical field. However, there are large overlaps between this category and the subcategory under NLP, namely, relationship extraction. The following categorization criteria were implemented: if a publication could be dissected into a relationship (eg, drug-drug interaction, condition-treatment, and association rule mining) extraction, identification, or discovery, the publication was classified under the relationship extraction subcategory of NLP; otherwise, the publication was included in the data mining and knowledge discovery category. Multimedia Appendix 6 lists all 25 included UMLS publications related to data mining, knowledge discovery, data analysis, and text analysis.

#### UMLS in Degree-Related Theses

Notably, there are 13 doctoral theses [8-20] included from the ACM Digital Library that used the UMLS as a key component in conducting the research. I believe that it is very likely that there is greater use of the UMLS in doctoral or master theses that might not be captured through the title, abstract, or keywords. My own doctoral thesis used UMLS as a critical foundational tool to build a knowledge base; however, UMLS was not listed as a keyword.

#### UMLS for Digital Libraries

A digital library is another initial goal of UMLS. In this systematic literature analysis, 5 publications related to the UMLS and a digital library were identified. Of these, one publication used machine learning to process information extracted from a digital library, in which UMLS served as an information source [21]. In terms of a digital library, UMLS is also used for navigation purposes [22], for the semantic query [23], to improve the functions of the digital library [24], and to extract knowledge from a digital library [25]. There could be additional publications on the topic that do not necessarily use *digital library* as the key term.

#### UMLS in Information Retrieval

Since its inception, UMLS has been used to achieve and improve information retrieval. A total of 125 publications were identified in this theme, which is the third most active theme in this review. The subtopics of this emerging theme include image retrieval (eg, radiological images, pathological images, microscopic images, computed tomography scans, and electrocardiograms), indexing, information retrieval (including information needs), information retrieval systems, and search engines (eg, PubMed, MEDLINE, electronic health record systems, books, databases of texts, images, and sounds), performance or correct measures (including ranking), and query (from generic queries, query formulation, query expansion, and more accurate queries to evaluations). The information sources for retrieval purposes included documents, information within documents, metadata, scientific literature, and patient records. Multimedia Appendix 7 lists all 125 UMLS publications related to information retrieval.

#### UMLS in Medical Education

UMLS was planned for use in medical education [1,26-29]. Most of the publications in this category included curriculum mapping [30], continuing education [31,32], problem-based learning [33], tutoring systems [33-41], and educational resource development [31,32,42-44].

#### UMLS in Different Medical Subdomains

As the most comprehensive collection of medical terminologies, UMLS has been used in 34 medical subdomains in a variety of ways. The subdomains in which UMLS has been used include Alzheimer disease [45,46], anatomical structure [47-64], appendectomy [65], asthma [66,67], blood transfusion [68,69], breast biopsy [65], breast cancer [70,71], cardiovascular diseases [72-74], colorectal cancer [75,76], depression [77,78], dilated cardiomyopathies [79], epidemiology [80,81], falling injury risk assessment [82], HIV [83], hypertension [84-86], Kawasaki disease [87], liver cancer [88], liver diseases [89,90], lupus [91], neuropsychiatric disorders [92-94], occupational medicine [95,96], oncology [97,98], Parkinson disease [99], pneumonia [100], physical therapy [101], primary care [102-104], prostate cancer [105,106], rare diseases [107-111], respiratory tract infection [112], stroke thrombolysis [113], surveillance [114-116], traditional Chinese medicine [117,118], urology [119,120], and Zika virus [121]. There are significantly more publications about anatomy than about any other medical subdomain.

#### UMLS in NLP

UMLS is used as a critical component in NLP, the most active theme in the review, with 230 publications identified. The specific use of UMLS in this category includes abbreviation-related studies, feature identification, lexicon and inventory, semantic-related studies, syntax-related studies, text classification, and other NLP-related UMLS publications.

Semantic-related publications (165/230, 71.7%) included concept recognition and extraction, named entity recognition, natural language, vocabulary, question generation, natural language understanding, relationship recognition and extraction, semantic similarity or relevance or distance, and word sense disambiguation. Named entity recognition also included negation recognition. For concept recognition or extraction, the following groups were included: adverse drug event identification, contextual property identification, disorder recognition, and identification of treatment information. Relationship recognition and extraction included association recognition, medication-indication relationships, drug-drug interaction, and disease-manifestation relationships.

Syntax-related publications (18/230, 7.8%) included part-of-speech tagging, parsing, and terminology extraction.

Other NLP-related publications (47/230, 20.4%) included rule-based NLP, statistical NLP, corpus development, morphological similarity, word embedding, and stemming.

The source document types used in NLP are very rich and include discharge summaries, problem lists, clinical trial eligibility criteria, clinical trial protocols, clinical narrative notes, patient records, radiology reports, neuroradiology reports, pathology reports, histology reports, emergency department reports, surgical operative reports, medical progress notes, literature, social media, emails, and forum posts. Multimedia Appendix 8 presents a list of all 230 publications classified into the NLP category.

#### UMLS-Based Ontology and Modeling-Related Publications

UMLS is also a common tool used in ontology, classification, taxonomy, modeling, knowledge representation, and their associated studies. Although UMLS and terminology study are 2 existing categories, there are still some publications that cannot be categorized into either of these categories. If a publication can be included in a more specific subcategory, for example, a model of an information retrieval system, then it will be classified into the information retrieval system and search engine subcategory instead of the modeling subcategory. In this category, the publications were classified into corresponding subcategories only if the publication could not be included in any other category. Multimedia Appendix 9 presents a list of all 80 publications in this category.

#### UMLS English-Language Publications About Non-English Languages

There are efforts related to using UMLS in languages other than English, as well as multilingual studies. In this category, 10 additional languages and 53 publications were identified. Some publications are related to automatic translation, whereas others are related to the coverage of an additional language of medical terms in addition to English. Languages other than English that relate to multilingual or cross-language uses of UMLS include Bulgarian [122], Dutch [123], French [63,124-145], German [76,146-149], Italian [150], Japanese [151-153], Korean [154-158], Portuguese [159], Spanish [160-162], and Swedish [146,163]. A total of 12 publications included more than two languages [146,159,163-172]. Clearly, there are more French-related UMLS publications than any other non-English language.

#### UMLS in Patient Care

One of the original goals of UMLS is to facilitate patient care directly or indirectly. As planned, UMLS has been used in patient care in many different ways, including the prediction of bariatric surgery outcomes, ensuring patient safety, development of a fall injury risk assessment instrument, patient outcome measurement, functional status measurement, clinical care quality assurance, computerized physician order entry, and clinical decision support systems. Multimedia Appendix 10 presents a list of all 35 publications in this category.

#### UMLS for Terminology Studies

As a critical tool, UMLS is used to conduct terminology studies. A total of 90 publications were classified into this category. The scope of the work includes a comparison of terminologies, construction of terminology, harmonization, interoperability, quality assurance, and other UMLS publications of terminology. UMLS is critical for achieving and advancing interoperability. The publications about the UMLS itself were classified into the UMLS category instead of under terminology studies. The roles of the UMLS in terminology studies include data sharing, aggregating data, harmonizing (including mapping among different terminologies), and vocabulary foundation. The publications on lexical mapping were classified into NLP. Multimedia Appendix 11 presents a list of all 90 UMLS publications on terminology studies.

#### Studies About the UMLS Itself

A total of 150 publications about the UMLS itself, which is the second most active theme after NLP, were identified. The scope of the publications ranged from auditing and enhancement of UMLS to the development of its own components, including Metathesaurus, SPECIALIST Lexicon and Lexical Tools, and Semantic Network, as well as its application tools MetaMap, MMTx, and SemRep. Furthermore, many efforts were related to increasing the coverage of UMLS in different subdomains, for example, in nursing, radiology, genetic disease, anatomy, and herbal supplements. In this category, the subtopics included applications or tools for UMLS, auditing of UMLS, components of UMLS, and coverage of UMLS. All studies in this category used UMLS as the study object. For example, auditing of UMLS includes publications on auditing-related studies that focus on the auditing of UMLS. If UMLS was used for other auditing purposes in a publication, then the publication was classified into UMLS in the other purposes category.

This category of publications also included modeling in UMLS. Other modeling-related publications that used UMLS were classified into the ontology and modeling categories. The publications that used UMLS to achieve different objectives (eg, identification of associations in texts) were classified into other categories based on their corresponding objectives. Multimedia Appendix 12 presents a list of all 150 UMLS publications on studies of the UMLS itself.

#### UMLS for Other Purposes

This category is used mainly for publications that use UMLS to achieve other purposes that cannot be covered by the themes noted above. In this category, auditing (not for UMLS auditing), consumer health, integrated system or data, and other research uses (including profile construction, management use, and deidentification) were included. Multimedia Appendix 13 presents a list of all 27 publications in this category.

## Discussion

### Summary and Interpretation of the Results

The results of the literature analysis showed the broad scope of the impact of UMLS in the academic world in the form of peer-reviewed journal publications, peer-reviewed conference publications, book chapters, and degree theses. What has been captured here, however, is only a *small fraction* of the real impact of UMLS. This literature analysis does not capture the following possible uses or impacts if no paper was published or if UMLS was not included in the title, abstract, or keywords: use of UMLS in the health information technology industry, health care delivery, software development, and any patent-related output.

The results show that UMLS has been broadly used, from basic science to applied projects in biomedical and health informatics. From the perspective of the number of publications, NLP, UMLS itself, and information retrieval are the 3 themes with the most publications. Anatomy is the medical subdomain with the most publications. French is the most active language, with a higher number of UMLS English-language publications of non-English languages. The large number of publications shows that certain themes are very active, although this literature analysis does not examine the overlap in different themes among different research projects. In addition, the number of publications should be used in a relative sense and with caution because a special issue of a journal or focused workshops or contests can skew the number of publications significantly.

In the *Results* section, the themes that repeatedly emerged during the literature analysis and synthesis have been listed. However, this is only an observation and a recording. From a purely ontological perspective, the same publications can be classified into different categories, depending on the axis. For example, a publication that focuses on automatic translation can be included in AI tools or applications; it can also be included in the multilingual category. Ideally, it will be useful to cross-reference each publication, which can then be classified into different categories. However, because of the large number of publications included in this literature analysis, such publications have been listed in only one category mostly (only 32/943, 3.4% of the publications was categorized into 2 categories; Table 1) instead of all possible categories. It is recognized that what was provided in this review is a *snapshot* of the publications at the gross anatomy level, not a *panoramic view* of the publications with every single detail at the molecular level. This literature analysis serves as an archive of English-language UMLS peer-reviewed publications. The themes and subtopics and the publications under each theme or subtopic show only one perspective, not the only perspective, on the publications and their organizations. It is recognized that the search strategies can find only those publications for which UMLS plays a critical role. Some additional publications may use UMLS in their work; however, if UMLS was not listed in the title, MeSH terms, or abstract, then these publications will not be found through the search strategies. Therefore, the real impact of UMLS, even as academic output, is far larger than this review can represent.

### Comparison With Existing UMLS-Use Publications

No systematic review or comprehensive literature analysis of UMLS was found during the literature search; however, there are publications on the use of UMLS through an analysis of UMLS annual reports [2] and the collection of surveys of UMLS users [173]. Nevertheless, the content of this literature analysis is complementary to these 2 studies [2,173]. The study by Fung et al [2] reported the geographical distribution of the users, the organizations of the UMLS license holders, types of information processed by UMLS, and areas of use of UMLS as well as users’ support, communications, and feedback. The study [2] drew conclusions from 1427 UMLS annual reports for the year 2004.

Chen et al [173] reported the results of a 26-item survey sent to those on a UMLS mailing list (>600 subscribers). The research team analyzed the responses from 70 respondents, provided detailed categories of the users’ employment and areas of use, and concluded that the top uses of UMLS were to access the source terminologies through UMLS and to achieve mapping among these terminologies. In addition, *terminology research, information retrieval, terminology translation, UMLS research,* and *NLP*, as well as *UMLS auditing,* were identified as the categories for the use of UMLS and as future priorities [173]. By comparison, this literature analysis paints a more comprehensive picture of publications in the last 30 years with regard to UMLS, by UMLS, and with UMLS. In analog language, this literature analysis is still at the level of *gross anatomy*; however, this review does provide more comprehensive categories, more detailed classifications, and clusters of publications on the topic. This literature analysis also lists degree-related doctoral theses in which the UMLS plays a critical role.

### About UMLS

The original intended uses of UMLS involved four main areas: patient care, medical education, library service, and product development [1]. Comparing the results of this literature analysis with the originally intended uses, it is concluded that, although the literature analysis reflects an output largely within academic settings, the original intended uses have been achieved successfully. There are multiple themes and subtopics that can be matched to each of the 4 areas. For example, the patient care and medical subdomains can be placed in the patient care category. It was, however, recognized that such a literature analysis is not the best way to capture all the uses of UMLS in the real world, especially with regard to product development. Nevertheless, it is acknowledged that many electronic health records, AI, and NLP applications in the health field commonly use UMLS [5].

UMLS has been a cornerstone of academic activities in biomedical informatics, health informatics, and health information technology as a way to facilitate interoperability in broad medical and health fields. This literature analysis demonstrates only a small fraction of the true impact of UMLS. UMLS can be used as a terminology hub that hosts the most commonly used biomedical and health terminologies worldwide by using a universal concept unique identifier. A terminology hub is different from terminology in the same way that SNOMED-CT (Systematized Nomenclature of Medicine-Clinical Terms) and UMLS are different but, at the same time, have some similarities. The 2 resources overlap but have mainly complementary purposes in the biomedical and health fields. SNOMED-CT is the most comprehensive medical terminology in the world, and UMLS includes SNOMED-CT and many additional terminologies. A common use of the UMLS is to provide machine-processable codes and meanings, which is similar to the use of SNOMED-CT; UMLS also provides mapping among different source terminologies. UMLS is critical for processing historical data and heterogeneous data sources, which will be a reality in health care in the near future. Therefore, to achieve seamless and effortless interoperability with a finer level of granularity in health care delivery sufficient to completely solve the puzzle described in e-patient Dave case study [174], at least at the front end, we need both SNOMED-CT and UMLS as well as many other resources.

However, UMLS is beyond a terminology hub. The intended uses of UMLS are mainly through software programs or systems. Many listed applications of UMLS include linking terms and codes in practice, pharmacy, and laboratory; facilitating mapping among different terminologies by providing terminology services; and serving as a lexical tool for NLP and AI, among others. Many additional UMLS applications have never been captured in the form of peer-reviewed publications. For example, my colleagues and I use UMLS as a teaching tool to introduce the concept of using controlled vocabularies to code medical records for health science major undergraduates.

### Future Work

This literature analysis provides a descriptive observation of English-language peer-reviewed publications on UMLS over the last 30 years. It is an overview of the publications in terms of scope, as well as major themes and subtopics. More detailed content and literature analysis can be conducted for each theme. In this study, most of the publications were examined through an analysis of titles and abstracts, with some full-text publications when necessary. A more detailed full-text publication analysis may provide a more in-depth understanding of this topic.

Another possible direction is to examine the overlap among different themes and subtopics. For example, future research could analyze the overlaps by classifying a publication into as many categories as possible. If a publication has only 1 position within 1 theme or one subtopic, a theme graph can be generated with all themes and subtopics (a graphical representation of Table 1) and all publications within each theme and each subtopic. Each publication would then have multiple positions in the theme graph. A visualization to consider the aggregated overlap (the same publication with multiple positions among multiple subtopics) among themes and subtopics can show or even inspire possible research collaboration opportunities among themes and subtopics.

### Conclusions

This comprehensive literature analysis provides an overview with systematic evidence of the UMLS English-language peer-reviewed publications in the last 30 years. The analysis provides a descriptive observation of the themes and their subtopics of the publications and provides a detailed list of the publications in each category. UMLS has been used and published successfully in patient care, medical education, digital libraries, and software development in biomedicine, as well as in degree-related theses, building AI tools, data mining and knowledge discovery, and many more foundational works in methodology and middle layers that may lead to advanced products. The results, although largely in academia, demonstrate that UMLS achieves its intended uses successfully and has been used successfully and broadly beyond its original intentions. NLP, UMLS itself, and information retrieval are the three themes with the most publications. Anatomy is the most active medical subdomain. French is the most active language among the UMLS English-language publications of non-English languages. Nevertheless, this systematic literature analysis only captures publications in the English language; therefore, it should not be treated as a comprehensive impact description of UMLS, which should include English-language peer-reviewed publications and much more (eg, other language publications, patents, software, apps, care quality, and patient safety).

## Appendix

Multimedia Appendix 1 Word cloud for all titles included in this systematic literature analysis (publication counts).

Multimedia Appendix 2 PRISMA (Preferred Reporting Items for Systematic Reviews and Meta-Analyses) extension for Scoping Reviews checklist.

Multimedia Appendix 3 The yearly number of publications included in the literature analysis from 1990 to 2020.

Multimedia Appendix 4 Themes and major topics of applications of the Unified Medical Language System.

Multimedia Appendix 5 Unified Medical Language System is used for building artificial intelligence applications and tools.

Multimedia Appendix 6 Publications on data mining, knowledge discovery, and text analysis using the Unified Medical Language System.

Multimedia Appendix 7 Unified Medical Language System publications related to information retrieval.

Multimedia Appendix 8 Unified Medical Language System in publications related to natural language processing.

Multimedia Appendix 9 Unified Medical Language System publications in ontology and modeling.

Multimedia Appendix 10 Publications that used the Unified Medical Language System in patient care.

Multimedia Appendix 11 Unified Medical Language System publications about terminology studies.

Multimedia Appendix 12 Publications about studies of the Unified Medical Language System itself.

Multimedia Appendix 13 Unified Medical Language System publications for purposes other than the themes noted above.

## Abbreviations

AI: artificial intelligence
MeSH: Medical Subject Headings
NIH: National Institutes of Health
NLP: natural language processing
SNOMED-CT: Systematized Nomenclature of Medicine-Clinical Terms
UMLS: Unified Medical Language System

## References

[1] Humphreys BL, Lindberg DA, Hole WT (1991). Assessing and enhancing the value of the UMLS Knowledge Sources. Proc Annu Symp Comput Appl Med Care.

[2] Fung KW, Hole WT, Srinivasan S (2006). Who is using the UMLS and how - insights from the UMLS user annual reports. AMIA Annu Symp Proc.

[3] Bodenreider O, McCray AT (2003). Exploring semantic groups through visual approaches. J Biomed Inform.

[4] (2004). Unified Medical Language System (UMLS). National Library of Medicine.

[5] Bodenreider O (2004). The Unified Medical Language System (UMLS): integrating biomedical terminology. Nucleic Acids Res.

[6] Moher D, Liberati A, Tetzlaff J, Altman DG (2009). Preferred reporting items for systematic reviews and meta-analyses: the PRISMA statement. Br Med J.

[7] Murphy SN, Barnett GO (1996). Achieving automated narrative text interpretation using phrases in the electronic medical record. Proc AMIA Annu Fall Symp.

[8] Fu LS (1992). A public domain unified medical language system (UMLS) patient database. Theses and dissertations: The University of Utah, Salt Lake City, UT.

[9] Chen Y (2008). Abstraction, Extension and Structural Auditing With the UMLS Semantic Network.

[10] Assefa S (2009). Human Conceptual Representation and Knowledge Structure in The UMLS: A Coherence Analysis.

[11] Mcinnes BT (2009). Supervised and Knowledge-Based Methods for Disambiguating Terms in Biomedical Text Using the UMLS and Metamap.

[12] Zhang L (2004). Enriching and Designing Metaschemas for the UMLS Network.

[13] Min H (2006). Structural Auditing Methodologies for Controlled Terminologies.

[14] Fowler RG, Gorry GA (1995). The virtual object model for distributed hypertext. Theses and dissertations: Rice University.

[15] Leroy GA, Chen H (2003). Facilitating knowledge discovery by integrating bottom-up and top-down knowledge sources: a text mining approach. Dissertations & Theses: The University of Arizona.

[16] Gu H (1999). Developing Techniques for Enhancing Comprehensibility of Controlled Medical Terminologies.

[17] Ruiz ME, Srinivasan P (2001). Combining Machine Learning and Hierarchical Structures for Text Categorization.

[18] An YJ (2008). Ontology Learning for the Semantic Deep Web.

[19] Zhou W (2008). Knowledge-Intensive Conceptual Retrieval of Biomedical Literature.

[20] Liu H (2002). Corpus-Based Ambiguity Resolution of Biomedical Terms Using Knowledge Bases and Machine Learning.

[21] Hu X, Lin TY, Song IY (2006). A semi-supervised efficient learning approach to extract biological relationships from web-based biomedical digital library. Web Intelli Agent Sys.

[22] McCray AT (2000). Digital library research and application. Stud Health Technol Inform.

[23] Kim EH, Oh JS, Song M, Allen R, Hunter J, Zeng M (2015). Exploring context-sensitive query reformulation in a biomedical digital library. Digital Libraries: Providing Quality Information.

[24] Robinson J, de Lusignan S, Kostkova P, Madge B (2006). Using UMLS to map from a library to a clinical classification: improving the functionality of a digital library. Stud Health Technol Inform.

[25] Mendonça EA, Cimino JJ (2000). Automated knowledge extraction from MEDLINE citations. Proc AMIA Symp.

[26] Denny JC, Bastarache L, Sastre EA, Spickard A (2009). Tracking medical students' clinical experiences using natural language processing. J Biomed Inform.

[27] Kanter SL, Miller RA, Tan M, Schwartz J (1994). Using POSTDOC to recognize biomedical concepts in medical school curricular documents. Bull Med Libr Assoc.

[28] Kanter SL (1993). Using the UMLS to represent medical curriculum content. Proc Annu Symp Comput Appl Med Care.

[29] Denny JC, Smithers JD, Miller RA, Spickard A (2003). "Understanding" medical school curriculum content using KnowledgeMap. J Am Med Inform Assoc.

[30] Komenda M, Schwarz D, Švancara J, Vaitsis C, Zary N, Dušek L (2015). Practical use of medical terminology in curriculum mapping. Comput Biol Med.

[31] Eysenbach G, Bauer J, Sager A, Bittorf A, Simon M, Diepgen T (1998). An international dermatological image atlas on the WWW: practical use for undergraduate and continuing medical education, patient education and epidemiological research. Stud Health Technol Inform.

[32] Kumar A, Quaglini S, Stefanelli M, Ciccarese P, Caffi E (2003). Modular representation of the guideline text: an approach for maintaining and updating the content of medical education. Med Inform Internet Med.

[33] Kazi H (2007). A diverse and robust tutoring system for medical problem-based learning. Proceeding of the 15th International Conference on Computers in Education, ICCE 2007.

[34] Kazi H, Haddawy P, Suebnukarn S (2013). Clinical reasoning gains in medical PBL: an UMLS based tutoring system. J Intell Inf Syst.

[35] Kazi H, Haddawy P, Suebnukarn S (2012). Employing UMLS for generating hints in a tutoring system for medical problem-based learning. J Biomed Inform.

[36] Kazi H, Haddawy P, Suebnukarn S (2007). Enriching Solution Space for Robustness in an Intelligent Tutoring System. Proceedings of the 2007 Conference on Supporting Learning Flow Through Integrative Technologies.

[37] Kazi H, Haddawy P, Suebnukarn S (2009). Expanding the space of plausible solutions in a medical tutoring system for problem-based learning. Int J Artif Intell Edu.

[38] Kazi H, Haddawy P, Suebnukarn S, Aleven V, Kay J, Mostow J (2010). Leveraging a domain ontology to increase the quality of feedback in an intelligent tutoring system. Intelligent Tutoring Systems.

[39] Kazi H, Haddawy P, Suebnukarn S (2008). Expanding the plausible solution space for robustness in an intelligent tutoring system. Intelligent Tutoring Systems.

[40] Kazi H, Haddawy P, Suebnukarn S (2011). METEOR: medical tutor employing ontology for robustness. Proceedings of the 16th International Conference on Intelligent User Interfaces.

[41] Suebnukarn S, Haddawy P, Rhienmora P (2008). A collaborative medical case authoring environment based on the UMLS. J Biomed Inform.

[42] Zeng Y, Liu X, Wang Y, Shen F, Liu S, Rastegar-Mojarad M, Wang L, Liu H (2017). Recommending education materials for diabetic questions using information retrieval approaches. J Med Internet Res.

[43] Zou H, Lu QC, Durack JC, Chao C, Strasberg HR, Zhang Y, Tsai M, Melmon K, Hahn JS (2001). Structured data management--the design and implementation of a web-based video archive prototype. Proc AMIA Symp.

[44] Denny JC, Irani PR, Wehbe FH, Smithers JD, Spickard A (2003). The KnowledgeMap project: development of a concept-based medical school curriculum database. AMIA Annu Symp Proc.

[45] Song M, Heo GE, Lee D (2014). Identifying the landscape of Alzheimer’s disease research with network and content analysis. Scientometrics.

[46] Dramé K, Diallo G, Delva F, Dartigues JF, Mouillet E, Salamon R, Mougin F (2014). Reuse of termino-ontological resources and text corpora for building a multilingual domain ontology: an application to Alzheimer's disease. J Biomed Inform.

[47] Talos I, Rubin DL, Halle M, Musen M, Kikinis R (2008). A prototype symbolic model of canonical functional neuroanatomy of the motor system. J Biomed Inform.

[48] Rosse C, Mejino JL (2003). A reference ontology for biomedical informatics: the Foundational Model of Anatomy. J Biomed Inform.

[49] Pyysalo S, Ananiadou S (2014). Anatomical entity mention recognition at literature scale. Bioinformatics.

[50] Rosse C, Ben Said M, Eno KR, Brinkley JF (1995). Enhancements of anatomical information in UMLS knowledge sources. Proc Annu Symp Comput Appl Med Care.

[51] Sato L, McClure RC, Rouse RL, Schatz CA, Greenes RA (1992). Enhancing the Metathesaurus with clinically relevant concepts: anatomic representations. Proc Annu Symp Comput Appl Med Care.

[52] Tran LT, Divita G, Carter ME, Judd J, Samore MH, Gundlapalli AV (2015). Exploiting the UMLS Metathesaurus for extracting and categorizing concepts representing signs and symptoms to anatomically related organ systems. J Biomed Inform.

[53] Bean CA (1997). Formative evaluation of a frame-based model of locative relationships in human anatomy. Proc AMIA Annu Fall Symp.

[54] Sneiderman CA, Rindflesch TC, Bean CA (1998). Identification of anatomical terminology in medical text. Proc AMIA Symp.

[55] Hishiki T, Ogasawara O, Tsuruoka Y, Okubo K (2004). Indexing anatomical concepts to OMIM Clinical Synopsis using the UMLS Metathesaurus. In Silico Biol.

[56] Bashyam V, Taira RK (2005). Indexing anatomical phrases in neuro-radiology reports to the UMLS 2005AA. AMIA Annu Symp Proc.

[57] Melgar HA, Beppler FD, Pacheco RC (2010). Knowledge retrieval in the anatomical domain. Proceedings of the 1st ACM International Health Informatics Symposium.

[58] Rosse C, Mejino JL, Modayur BR, Jakobovits R, Hinshaw KP, Brinkley JF (1998). Motivation and organizational principles for anatomical knowledge representation: the digital anatomist symbolic knowledge base. J Am Med Inform Assoc.

[59] Bowden DM, Song E, Kosheleva J, Dubach MF (2012). NeuroNames: an ontology for the BrainInfo portal to neuroscience on the web. Neuroinformatics.

[60] Mork P, Brinkley JF, Rosse C (2003). OQAFMA Querying agent for the Foundational Model of Anatomy: a prototype for providing flexible and efficient access to large semantic networks. J Biomed Inform.

[61] Cerveri P, Masseroli M, Pinciroli F (2000). Remote access to anatomical information: an integration between semantic knowledge and visual data. Proc AMIA Symp.

[62] Mejino JL, Rosse C (1998). The potential of the digital anatomist foundational model for assuring consistency in UMLS sources. Proc AMIA Symp.

[63] Merabti T, Soualmia LF, Grosjean J, Palombi O, Müller J, Darmoni SJ (2011). Translating the Foundational Model of Anatomy into French using knowledge-based and lexical methods. BMC Med Inform Decis Mak.

[64] Lowe HJ, Huang Y, Regula DP (2009). Using a statistical natural language Parser augmented with the UMLS specialist lexicon to assign SNOMED CT codes to anatomic sites and pathologic diagnoses in full text pathology reports. AMIA Annu Symp Proc.

[65] Lamiell JM, Wojcik ZM, Isaacks J (1993). Computer auditing of surgical operative reports written in English. Proc Annu Symp Comput Appl Med Care.

[66] Gabb HA, Blake C (2016). An informatics approach to evaluating combined chemical exposures from consumer products: a case study of asthma-associated chemicals and potential endocrine disruptors. Environ Health Perspect.

[67] Choong MK, Tsafnat G, Hibbert P, Runciman WB, Coiera E (2017). Linking clinical quality indicators to research evidence - a case study in asthma management for children. BMC Health Serv Res.

[68] Achour SL, Dojat M, Rieux C, Bierling P, Lepage E (2001). A UMLS-based knowledge acquisition tool for rule-based clinical decision support system development. J Am Med Inform Assoc.

[69] Achour S, Dojat M, Brethon JM, Blain G, Lepage E, Horn W, Shahar Y, Lindberg G, Andreassen S, Wyatt J (1999). The use of the UMLS knowledge sources for the design of a domain specific ontology: a practical experience in blood transfusion. Artificial Intelligence in Medicine.

[70] Herskovic JR, Subramanian D, Cohen T, Bozzo-Silva PA, Bearden CF, Bernstam EV (2012). Graph-based signal integration for high-throughput phenotyping. BMC Bioinformatics.

[71] Zeng Z, Espino S, Roy A, Li X, Khan SA, Clare SE, Jiang X, Neapolitan R, Luo Y (2018). Using natural language processing and machine learning to identify breast cancer local recurrence. BMC Bioinformatics.

[72] Jadhav A, Sheth A, Pathak J (2014). Analysis of online information searching for cardiovascular diseases on a consumer health information portal. AMIA Annu Symp Proc.

[73] Varghese J, Sünninghausen S, Dugas M (2015). Standardized cardiovascular quality assurance forms with multilingual support, UMLS coding and medical concept analyses. Stud Health Technol Inform.

[74] Shivade C, Malewadkar P, Fosler-Lussier E, Lai AM (2015). Comparison of UMLS terminologies to identify risk of heart disease using clinical notes. J Biomed Inform.

[75] Martínez M, Vázquez JM, Pereira J, Pazos A (2008). Annotation of colorectal cancer data using the UMLS Metathesaurus. Knowledge-Based Intelligent Information and Engineering Systems.

[76] Becker M, Kasper S, Böckmann B, Jöckel K, Virchow I (2019). Natural language processing of German clinical colorectal cancer notes for guideline-based treatment evaluation. Int J Med Inform.

[77] Du Y, Lin S, Huang Z (2018). Making semantic annotation on patient data of depression. Proceedings of the 2nd International Conference on Medical and Health Informatics.

[78] Kossman S, Jones J, Brennan PF (2007). Tailoring online information retrieval to user's needs based on a logical semantic approach to natural language processing and UMLS mapping. AMIA Annu Symp Proc.

[79] Gabetta M, Larizza C, Bellazzi R (2013). A Unified Medical Language System (UMLS) based system for Literature-Based Discovery in medicine. Stud Health Technol Inform.

[80] Kim H, Song S, Kim Y, Song M (2014). A display of conceptual structures in the epidemiologic literature. Proceedings of the ACM 8th International Workshop on Data and Text Mining in Bioinformatics.

[81] Xu H, Lu Y, Jiang M, Liu M, Denny JC, Dai Q, Peterson NB (2010). Mining biomedical literature for terms related to epidemiologic exposures. AMIA Annu Symp Proc.

[82] Currie LM, Mellino LV, Cimino JJ, Bakken S (2004). Development and representation of a fall-injury risk assessment instrument in a clinical information system. Stud Health Technol Inform.

[83] Bates J, Fodeh SJ, Brandt CA, Womack JA (2016). Classification of radiology reports for falls in an HIV study cohort. J Am Med Inform Assoc.

[84] Kumar A, Ciccarese P, Smith B, Piazza M (2004). Context-based task ontologies for clinical guidelines. Stud Health Technol Inform.

[85] Kumar A, Ciccarese P, Quaglini S, Stefanelli M, Caffi E, Boiocchi L (2003). Relating UMLS semantic types and task-based ontology to computer-interpretable clinical practice guidelines. Stud Health Technol Inform.

[86] Campbell JR, Kallenberg GA, Sherrick RC (1992). The clinical utility of META: an analysis for hypertension. Proc Annu Symp Comput Appl Med Care.

[87] Doan S, Maehara CK, Chaparro JD, Lu S, Liu R, Graham A, Berry E, Hsu C, Kanegaye JT, Lloyd DD, Ohno-Machado L, Burns JC, Tremoulet AH, Pediatric Emergency Medicine Kawasaki Disease Research Group (2016). Building a natural language processing tool to identify patients with high clinical suspicion for Kawasaki disease from Emergency Department notes. Acad Emerg Med.

[88] Ganzinger M, Knaup P (2013). Semantic prerequisites for data sharing in a biomedical research network. Stud Health Technol Inform.

[89] Marquet G, Burgun A, Moussouni F, Guérin E, Le Duff F, Loréal O (2003). BioMeKe: an ontology-based biomedical knowledge extraction system devoted to transcriptome analysis. Stud Health Technol Inform.

[90] Guérin E, Marquet G, Burgun A, Loréal O, Berti-Equille L, Leser U, Moussouni F, Ludäscher B, Raschid L (2005). Integrating and warehousing liver gene expression data and related biomedical resources in GEDAW. Data Integration in the Life Sciences.

[91] Turner CA, Jacobs AD, Marques CK, Oates JC, Kamen DL, Anderson PE, Obeid JS (2017). Word2Vec inversion and traditional text classifiers for phenotyping lupus. BMC Med Inform Decis Mak.

[92] Lyalina S, Percha B, LePendu P, Iyer SV, Altman RB, Shah NH (2013). Identifying phenotypic signatures of neuropsychiatric disorders from electronic medical records. J Am Med Inform Assoc.

[93] Zolnoori M, Fung KW, Patrick TB, Fontelo P, Kharrazi H, Faiola A, Wu YS, Eldredge CE, Luo J, Conway M, Zhu J, Park SK, Xu K, Moayyed H, Goudarzvand S (2019). A systematic approach for developing a corpus of patient reported adverse drug events: a case study for SSRI and SNRI medications. J Biomed Inform.

[94] Van Le D, Montgomery J, Kirkby KC, Scanlan J (2018). Risk prediction using natural language processing of electronic mental health records in an inpatient forensic psychiatry setting. J Biomed Inform.

[95] Silverstein SM, Miller PL, Cullen MR (1993). An information sources map for Occupational and Environmental Medicine: guidance to network-based information through domain-specific indexing. Proc Annu Symp Comput Appl Med Care.

[96] Harber P, Leroy G (2017). Feasibility and utility of lexical analysis for occupational health text. J Occup Environ Med.

[97] Sherertz DD, Tuttle MS, Olson NE, Hsu GT, Carlson RW, Fagan LM, Acuff RD, Cole WG, Nelson SJ (1995). Accessing oncology information at the point of care: experience using speech, pen, and 3-D interfaces with a knowledge server. Medinfo.

[98] Berman JJ, Henson DE (2003). Classifying the precancers: a metadata approach. BMC Med Inform Decis Mak.

[99] Sneiderman CA, Rindflesch TC, Aronson AR (1996). Finding the findings: identification of findings in medical literature using restricted natural language processing. Proc AMIA Annu Fall Symp.

[100] Bejan CA, Xia F, Vanderwende L, Wurfel MM, Yetisgen-Yildiz M (2012). Pneumonia identification using statistical feature selection. J Am Med Inform Assoc.

[101] Hardardottir A, Heimisdottir M, Aronson AR, Gunnarsdottir V (2008). Standardized documentation in physical therapy: testing of validity and reliability of the PT-ITC and mapping it to the Metathesaurus. AMIA Annu Symp Proc.

[102] Westberg EE, Miller RA (1999). The basis for using the internet to support the information needs of primary care. J Am Med Inform Assoc.

[103] He Z, Halper M, Perl Y, Elhanan G (2012). Clinical clarity versus terminological order - the readiness of SNOMED CT concept descriptors for primary care. MIXHS 12 (2012).

[104] Mullins HC, Scanland PM, Collins D, Treece L, Petruzzi P, Goodson A, Dickinson M (1996). The efficacy of SNOMED, Read Codes, and UMLS in coding ambulatory family practice clinical records. Proc AMIA Annu Fall Symp.

[105] Heintzelman NH, Taylor RJ, Simonsen L, Lustig R, Anderko D, Haythornthwaite JA, Childs LC, Bova GS (2013). Longitudinal analysis of pain in patients with metastatic prostate cancer using natural language processing of medical record text. J Am Med Inform Assoc.

[106] Overton JA, Romagnoli C, Chhem R (2011). Open Biomedical Ontologies applied to prostate cancer. Appl Ontol.

[107] Fung KW, Richesson R, Bodenreider O (2014). Coverage of rare disease names in standard terminologies and implications for patients, providers, and research. AMIA Annu Symp Proc.

[108] Darmoni SJ, Soualmia LF, Letord C, Jaulent M, Griffon N, Thirion B, Névéol A (2012). Improving information retrieval using Medical Subject Headings Concepts: a test case on rare and chronic diseases. J Med Libr Assoc.

[109] Rance B, Snyder M, Lewis J, Bodenreider O (2013). Leveraging terminological resources for mapping between rare disease information sources. Stud Health Technol Inform.

[110] Brandt M, Rath A, Devereau A, Aymé S, Peleg M, Lavrač N, Combi C (2011). Mapping orphanet terminology to UMLS. Artificial Intelligence in Medicine.

[111] Andrews JE, Shereff D, Patrick T, Richesson R (2010). The question about questions: is DC a good choice to address the challenges of representation of clinical research questions and value sets?. Proceedings of the DCMI International Conference on Dublin Core and Metadata Applications.

[112] Arif K, Qamar U, Wahab K, Riaz M (2019). Building a biomedical ontology for respiratory tract infection. Proceedings of the 2019 7th International Conference on Computer and Communications Management.

[113] Sung S, Chen K, Wu DP, Hung L, Su Y, Hu Y (2018). Applying natural language processing techniques to develop a task-specific EMR interface for timely stroke thrombolysis: a feasibility study. Int J Med Inform.

[114] Lu H, King C, Wu T, Shih F, Hsiao J, Zeng D, Chen H (2007). Chinese chief complaint classification for syndromic surveillance. Intelligence and Security Informatics: Biosurveillance.

[115] Tolentino H, Matters M, Walop W, Law B, Tong W, Liu F, Fontelo P, Kohl K, Payne D (2006). Concept negation in free text components of vaccine safety reports. AMIA Annu Symp Proc.

[116] Chapman WW, Fiszman M, Dowling JN, Chapman BE, Rindflesch TC (2004). Identifying respiratory findings in emergency department reports for biosurveillance using MetaMap. Stud Health Technol Inform.

[117] Lau AS, Tse SH (2010). Development of the ontology using a problem-driven approach: in the context of traditional Chinese medicine diagnosis. Int J Knowl Eng.Data Min.

[118] Zhu X, Lee KP, Cimino JJ (2004). Knowledge representation of traditional Chinese acupuncture points using the UMLS and a terminology model. Proceedings of the IDEAS Workshop on Medical Information Systems: The Digital Hospital (IDEAS-DH'04).

[119] Burgun A, Botti G, Lukacs B, Mayeux D, Seka LP, Delamarre D, Bremond M, Kohler F, Fieschi M, Le Beux P (1994). A system that facilitates the orientation within procedure nomenclatures through a semantic approach. Med Inform (Lond).

[120] Burgun A, Delamarre D, Botti G, Lukacs B, Mayeux D, Bremond M, Kohler F, Fieschi M, Le Beux P (1994). Designing a sub-set of the UMLS knowledge base applied to a clinical domain: methods and evaluation. Proc Annu Symp Comput Appl Med Care.

[121] Moreira A, Alonso-Calvo R, Muñoz A, Crespo J (2017). Enhancing collaborative case diagnoses through unified medical language system-based disambiguation: a case study of the zika virus. Telemed J E Health.

[122] Nikolova I (2011). Angelova, identifying relations between medical concepts by parsing UMLS® definitions. Conceptual Structures for Discovering Knowledge.

[123] Afzal Z, Pons E, Kang N, Sturkenboom MC, Schuemie MJ, Kors JA (2014). ContextD: an algorithm to identify contextual properties of medical terms in a Dutch clinical corpus. BMC Bioinformatics.

[124] Deléger L, Merabti T, Lecrocq T, Joubert M, Zweigenbaum P, Darmoni S (2010). A twofold strategy for translating a medical terminology into French. AMIA Annu Symp Proc.

[125] Fabry P, Baud R, Burgun A, Lovis C (2006). Amplification of Terminologia anatomica by French language terms using Latin terms matching algorithm: a prototype for other language. Int J Med Inform.

[126] Merabti T, Massari P, Joubert M, Sadou E, Lecroq T, Abdoune H, Rodrigues J, Darmoni SJ (2010). An automated approach to map a French terminology to UMLS. Stud Health Technol Inform.

[127] Maisonnasse L, Harrathi F, Roussey C, Calabretto S (2009). Analysis combination and Pseudo relevance feedback in conceptual language model: LIRIS Participation at ImageCLEFMed. Multilingual Information Access Evaluation II. Multimedia Experiments.

[128] Joubert M, Abdoune H, Merabti T, Darmoni S, Fieschi M (2009). Assisting the translation of SNOMED CT into French using UMLS and four representative French-language terminologies. AMIA Annu Symp Proc.

[129] Abdoune H, Merabti T, Darmoni SJ, Joubert M (2011). Assisting the translation of the CORE subset of SNOMED CT into French. Stud Health Technol Inform.

[130] Grabar N, Varoutas P, Rizand P, Livartowski A, Hamon T (2008). Automatic acquisition of synonyms from French UMLS for enhanced search of EHRs. Stud Health Technol Inform.

[131] Le Duff F, Burgun A, Pouliquen B, Delamarre D, Le Beux P (1999). Automatic enrichment of the unified medical language system starting from the ADM knowledge base. Stud Health Technol Inform.

[132] Joubert M, Peretti A, Darmoni S, Dahamna B, Fieschi M (2006). Contribution to an automated indexing of French-language health web sites. AMIA Annu Symp Proc.

[133] Deléger L, Merkel M, Zweigenbaum P (2006). Contribution to terminology internationalization by word alignment in parallel corpora. AMIA Annu Symp Proc.

[134] Ruiz M, Névéol A (2007). Evaluation of Automatically Assigned MeSH Terms for Retrieval of Medical Images. Proceedings of the 8th Workshop of the Cross-Language Evaluation Forum, CLEF 2007.

[135] Tran TD, Garcelon N, Burgun A, Le Beux P (2004). Experiments in cross-language medical information retrieval using a mixing translation module. Stud Health Technol Inform.

[136] Besana P (2010). From french EHR to NCI ontology via UMLS. Proceedings of the 5th International Conference on Ontology Matching.

[137] Bodenreider O, McCray AT (1998). From French vocabulary to the Unified Medical Language System: a preliminary study. Stud Health Technol Inform.

[138] Le Duff F, Burgun A, Cleret M, Pouliquen B, Barac'h V, Le Beux P (2000). Knowledge acquisition to qualify Unified Medical Language System interconceptual relationships. Proc AMIA Symp.

[139] Merabti T, Abdoune H, Letord C, Sakji S, Joubert M, Darmoni SJ (2011). Mapping the ATC classification to the UMLS metathesaurus: some pragmatic applications. Stud Health Technol Inform.

[140] Delbecque T, Zweigenbaum P (2007). MetaCoDe: A lightweight UMLS mapping tool. Artificial Intelligence in Medicine.

[141] Bousquet C, Souvignet J, Merabti T, Sadou E, Trombert B, Rodrigues J (2012). Method for mapping the French CCAM terminology to the UMLS metathesaurus. Stud Health Technol Inform.

[142] Cossin S, Lebrun L, Lobre G, Loustau R, Jouhet V, Griffier R, Mougin F, Diallo G, Thiessard F (2019). Romedi: An open data source about French drugs on the semantic web. Stud Health Technol Inform.

[143] Ventura JA (2014). Towards a mixed approach to extract biomedical terms from text corpus. Int J Knowl Disc Bioinfo.

[144] Zweigenbaum P, Baud R, Burgun A, Namer F, Jarrousse E, Grabar N, Ruch P, Le Duff F, Thirion B, Darmoni S (2003). Towards a unified medical lexicon for French. Stud Health Technol Inform.

[145] Darmoni SJ, Jarrousse E, Zweigenbaum P, Le Beux P, Namer F, Baud R, Joubert M, Vallée H, Côté RA, Buemi A, Bourigault D, Recource G, Jeanneau S, Rodrigues JM (2003). VUMeF: extending the French involvement in the UMLS Metathesaurus. AMIA Annu Symp Proc.

[146] Markó K, Schulz S, Hahn U (2005). Automatic lexicon acquisition for a medical cross-language information retrieval system. Stud Health Technol Inform.

[147] Becker M, Böckmann B (2016). Extraction of UMLS® concepts using Apache cTAKES™ for German language. Stud Health Technol Inform.

[148] Weske-Heck G, Zaiss A, Zabel M, Schulz S, Giere W, Schopen M, Klar R (2002). The German specialist lexicon. Proc AMIA Symp.

[149] Widdows D, Peters S, Cederberg S, Chan C, Steffen D, Buitelaar P (2003). Unsupervised monolingual and bilingual word-sense disambiguation of medical documents using UMLS. Proceedings of the ACL 2003 Workshop on Natural Language Processing in Biomedicine.

[150] Chiaramello E, Pinciroli F, Bonalumi A, Caroli A, Tognola G (2016). Use of "off-the-shelf" information extraction algorithms in clinical informatics: a feasibility study of MetaMap annotation of Italian medical notes. J Biomed Inform.

[151] Nishimoto N, Terae S, Uesugi M, Ogasawara K, Sakurai T (2008). Development of a medical-text parsing algorithm based on character adjacent probability distribution for Japanese radiology reports. Methods Inf Med.

[152] Onogi Y, Ohe K, Tanaka M, Nozoe A, Sasaki T, Sato M, Kikuchi Y, Shinohara T, Suzuki H, Kaihara S, Seyama Y (2004). Mapping Japanese medical terms to UMLS Metathesaurus. Stud Health Technol Inform.

[153] Nishimoto N, Satoshi T, Jiang G, Uesugi M, Terashita T, Tanikawa T, Endou A, Ogasawara K, Sakurai T (2006). Semantic distribution study of noun*noun compounds in the Japanese CT clinical reports. AMIA Annu Symp Proc.

[154] Han S, Kwak M, Kim S, Yoo S, Park H, Kijoo J, Kim J, Choi M, Choi J (2004). A comparative study on concept representation between the UMLS and the clinical terms in Korean medical records. Stud Health Technol Inform.

[155] Lee KN, Yoon J, Min WK, Lim HS, Song J, Chae SL, Jang S, Ki C, Bae SY, Kim JS, Kwon J, Lee CK, Yoon S (2008). Standardization of terminology in laboratory medicine II. J Korean Med Sci.

[156] Han S, Choi J (2005). The comparative study on concept representation between the UMLS and the clinical terms in Korean medical records. Int J Med Inform.

[157] Park HK, Choi J (2007). Towards chronological summary of medical records. AMIA Annu Symp Proc.

[158] Kang B, Kim D, Kim H (2009). Two-Phase chief complaint mapping to the UMLS metathesaurus in Korean electronic medical records. IEEE Trans Inf Technol Biomed.

[159] Ruiz ME, Southwick SB (2006). UB at CLEF 2005: Bilingual CLIR and medical image retrieval tasks. Accessing Multilingual Information Repositories.

[160] Carrero F, Cortizo JC, Gómez JM (2008). Building a Spanish MMTx by using automatic translation and biomedical ontologies. Intelligent Data Engineering and Automated Learning.

[161] Buendía F, Gayoso-Cabada J, Juanes-Méndez J, Martín-Izquierdo M (2019). Cataloguing Spanish medical reports with UMLS terms. Proceedings of the Seventh International Conference on Technological Ecosystems for Enhancing Multiculturality.

[162] Carrero F, Cortizo J, Gómez J, de Buenaga M (2008). In the development of a Spanish metamap. Proceedings of the 17th ACM Conference on Information and Knowledge Management.

[163] Markó K, Schulz S, Hahn U (2007). Automatic lexeme acquisition for a multilingual medical subword thesaurus. Int J Med Inform.

[164] Eichmann D, Ruiz M.E, Srinivasan P (1998). Cross-language information retrieval with the UMLS metathesaurus. Proceedings of the 21st Annual International ACM SIGIR Conference on Research and development in Information Retrieval.

[165] Tringali M, Hole WT, Srinivasan S (2002). Integration of a standard gastrointestinal endoscopy terminology in the UMLS Metathesaurus. Proc AMIA Symp.

[166] Hersh WR, Donohoe LC (1998). SAPHIRE International: a tool for cross-language information retrieval. Proc AMIA Symp.

[167] Göbel G, Andreatta S, Masser J, Pfeiffer KP (2001). A multilingual medical thesaurus browser for patients and medical content managers. Stud Health Technol Inform.

[168] Hellrich J, Hahn U (2014). Fostering Multilinguality in the UMLS: A computational approach to terminology expansion for multiple languages. AMIA Annu Symp Proc.

[169] Déjean H, Gaussier E, Sadat F (2002). An approach based on multilingual thesauri and model combination for bilingual lexicon extraction. Proceedings of the 19th International Conference on Computational Linguistics.

[170] Hellrich J, Hahn U (2014). Exploiting parallel corpora to scale up multilingual biomedical terminologies. Stud Health Technol Inform.

[171] Hellrich J, Schulz S, Buechel S, Hahn U (2015). JuFiT: A configurable rule engine for filtering and generating new multilingual UMLS terms. AMIA Annu Symp Proc.

[172] Guillén R (1998). Reusing translated terms to expand a multilingual thesaurus. Machine Translation and the Information Soup.

[173] Chen Y, Perl Y, Geller J, Cimino JJ (2007). Analysis of a study of the users, uses, and future agenda of the UMLS. J Am Med Inform Assoc.

[174] Hoyt R, Yoshihashi A (2014). Health Informatics: Practical Guide for Healthcare and Information Technology Professionals.

