# Supplementary figures and images for: The Unified Medical Language System at 30 Years and How It Is Used and Published: Systematic Review and Content Analysis
Source: JMIR Med Inform. 2021 Aug 27;9(8):e20675. doi: 10.2196/20675 (PMC8433943; doi:10.2196/20675)

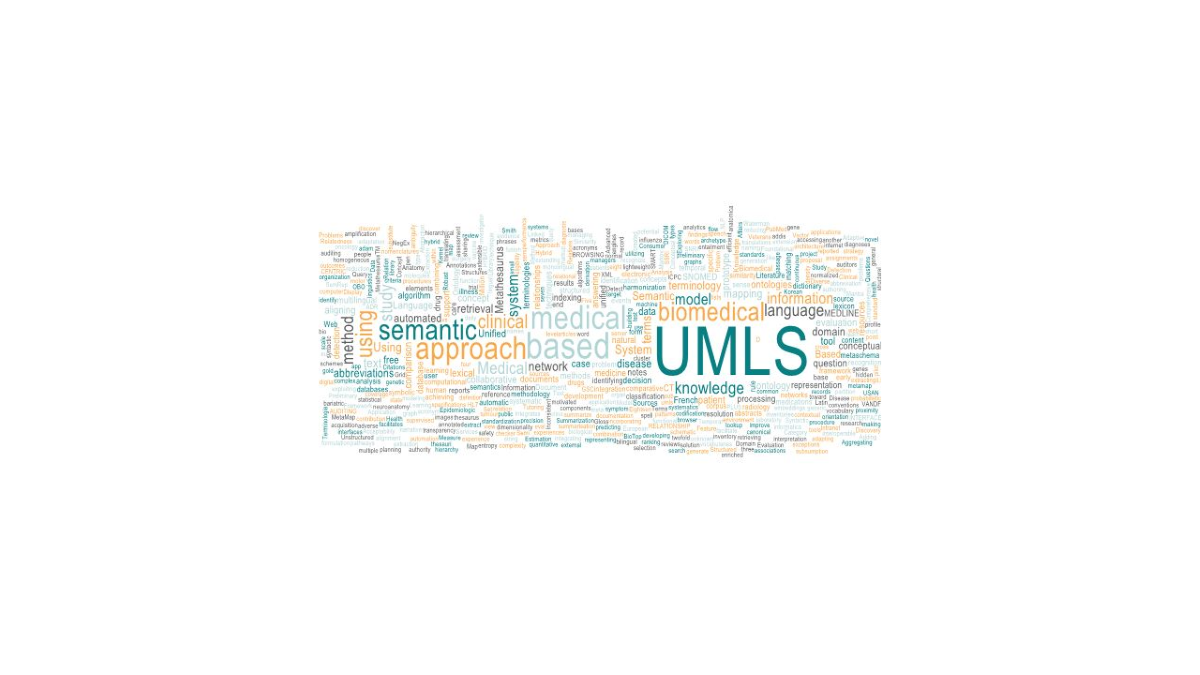

Supplement: Multimedia Appendix 1 [file medinform_v9i8e20675_app1.png]

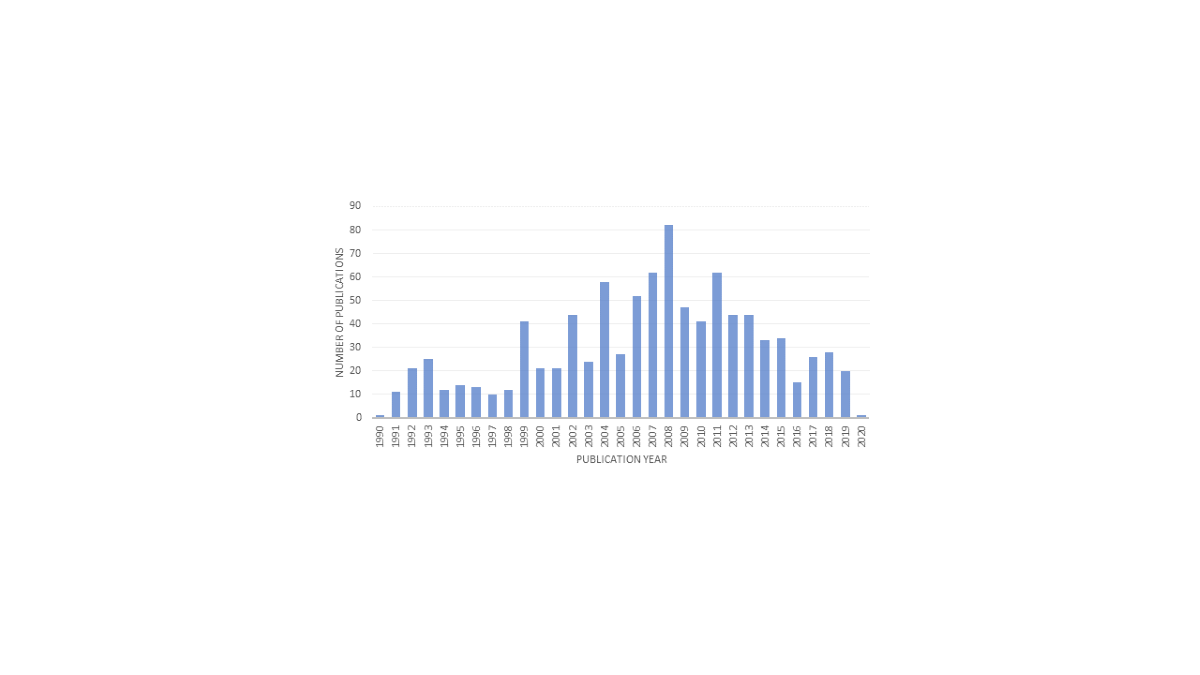

Supplement: Multimedia Appendix 3 [file medinform_v9i8e20675_app3.png]

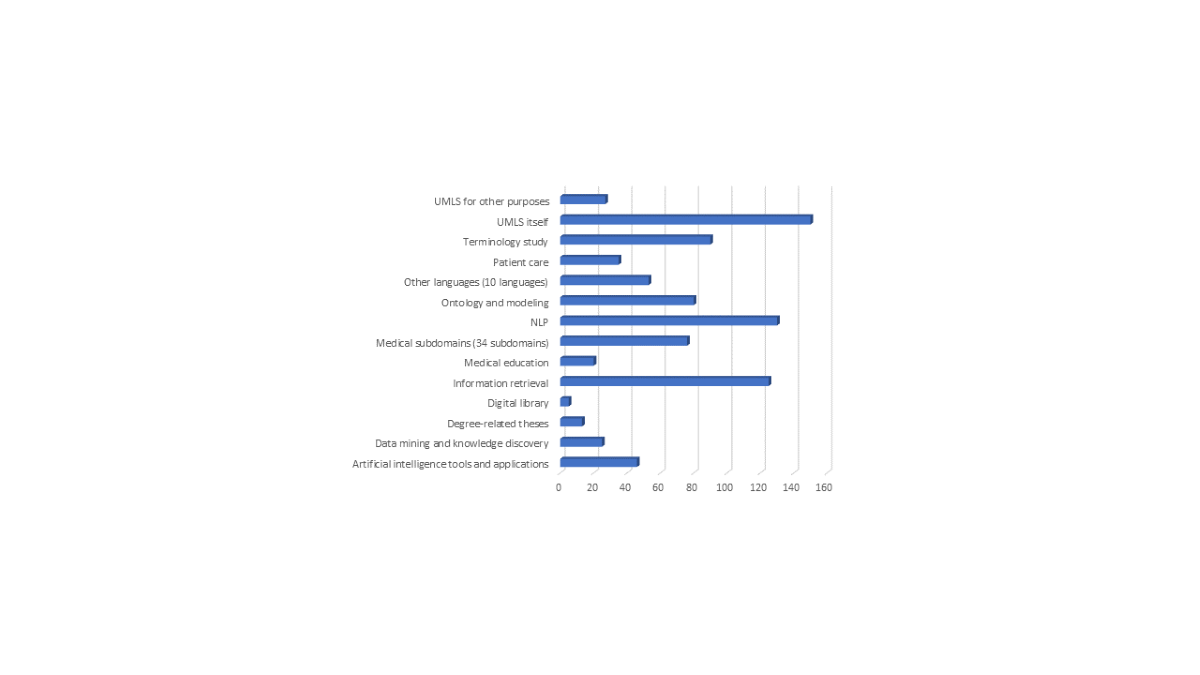

Supplement: Multimedia Appendix 4 [file medinform_v9i8e20675_app4.png]
