# Supplementary material for: The Unified Medical Language System at 30 Years and How It Is Used and Published: Systematic Review and Content Analysis
Source: JMIR Med Inform. 2021 Aug 27;9(8):e20675. doi: 10.2196/20675 (PMC8433943; doi:10.2196/20675)
Supplement: Multimedia Appendix 5 [file medinform_v9i8e20675_app5.pdf]

Multimedia Appendix 5. Unified Medical Language System is used for building artificial intelligence applications and tools.

Author	Publication year	Title	What was UMLS used for?
**Automated annotation/interpretation**			
Murphy, et al[1]	1996	Achieving automated narrative text interpretation using phrases in the electronic medical record	Automatic narrative text interpretation, UMLS, EMR
Eck, et al[2]	2004	Improving statistical machine translation in the medical domain using the unified medical language system	Automatic translation, UMLS, translation of dialogues between doctors and patients
Liu, et al[3]	2005	Automating tissue bank annotation from pathology reports - comparison to a gold standard expert annotation set	Automatic annotation, coding UMLS terms, pathology reports
Matykiewicz, et al[4]	2006	Nonambiguous concept mapping in medical domain	Automatic annotation, mapping medical texts to UMLS concepts
French, et al[5]	2009	Application and evaluation of automated semantic annotation of gene expression experiments	Automated semantic annotation, concept extraction, UMLS,
Gschwandtner, et al[6]	2010	Easing semantically enriched information retrieval-An interactive semi-automatic annotation system for medical documents	Automatic annotation, mapping narrative texts to UMLS concepts
Kim, et al[7]	2010	Unsupervised mapping of sentences to biomedical concepts based on integrated information retrieval model and clustering	Annotation of disease descriptions with UMLS concepts, dictionary-based simple matching methods
**Automatic coding**			
Rosenberg, et al[8]	1994	Acceptability of Unified Medical Language System terms as substitute for natural language general medicine clinic diagnoses	Automatic coding via UMLS concepts
Berman[9]	2004	Resources for comparing the speed and performance of medical autocoders	Medical autocoders, concept indexing
Friedman C, et al[10]	2004	Automated encoding of clinical documents based on natural language processing	Automatic coding, clinical documents, UMLS coding, extraction of relevant clinical information
Erdal, et al[11]	2007	ICD9 Code Assistant: A prototype	Automatic coding, UMLS clinical concept identification, ICD9

Becker, et al[12]	2017	CodeMapper: semiautomatic coding of case definitions. A contribution from the ADVANCE project	Automatic coding, identification of UMLS concepts
Helwe, et al[13]	2017	CCS Coding of Discharge Diagnoses via Deep Neural Networks	CCS coding, mapping discharge diagnoses into UMLS concepts, Deep Neural Networks
Varghese, et al[14]	2018	Web-Based Information Infrastructure Increases the Interrater Reliability of Medical Coders: Quasi-Experimental Study	Automatic coding, code suggestion system, UMLS
**Automatic summarization**			
Whalen, et al[15]	2005	Medical textbook summarization and guided navigation using statistical sentence extraction	Summarization, navigation, UMLS to provide conceptual relationships, clusters of concepts
Chen, et al[16]	2006	A Query-Based Medical Information Summarization System Using Ontology Knowledge	Summarization, UMLS for information retrieval,
Reeve, et al[17]	2006	BioChain: lexical chaining methods for biomedical text summarization	Summarization, UMLS for term identification, text-to-concept mapping
Park, et al[18]	2007	Towards chronological summary of medical records	Summarization, patient's records, UMLS to identify terms
Reeve, et al[19]	2007	Biomedical text summarisation using concept chains	Summarization, link semantically-related concepts
Morales, et al[20]	2008	Concept-graph based biomedical automatic summarization using ontologiesMoreal, et al	Summarization, UMLS to identify accurate semantics
Plaza, et al[21]	2010	Improving summarization of biomedical documents using word sense disambiguation	Summarization, graphs by UMLS concepts and relations
Plaza, et al[22]	2011	A semantic graph-based approach to biomedical summarisation	Summarization, UMLS concepts and relations to index documents
Plaza, et al[23]	2011	Studying the correlation between different word sense disambiguation methods and summarization effectiveness in biomedical texts	Summarization, MetaMap, UMLS mapping between documents and concepts
Plaza, et al[24]	2012	Resolving ambiguity in biomedical text to improve summarization	Summarization, MetaMap, UMLS mapping between documents and concepts
Roy, et al[25]	2012	A method to summarize Disease Based Temporal state of human organ	Disease Based Temporal Score (DT-Score), temporal

		using laboratory test data and UMLS knowledge	state of an organ, lab test results summarization
Mordi, et al[26]	2017	Quantifying the informativeness for biomedical literature summarization: An itemset mining method	Summarization, UMLS for mapping between documents to concepts
Azadani, et al[27]	2018	Graph-based biomedical text summarization: An itemset mining and sentence clustering approach	Summarization, UMLS is used to build a concept-based model
Kim, et al[28]	2018	Personalised health document summarisation exploiting Unified Medical Language System and topic-based clustering for mobile healthcare	Multi-document summarization, UMLS, personal health record
Moradi, et al[29]	2018	Different approaches for identifying important concepts in probabilistic biomedical text summarization	Summarization, UMLS for mapping to input texts, feature selection
**Question-answering systems**			
Delbecque T, et al[30]	2005	Indexing UMLS Semantic Types for Medical Question-Answering	Question answering system, UMLS semantic types, name entity recognition
Wedgwood J[31]	2005	MQAF: a medical question-answering framework	Question answering system, UMLS, MetaMap, question taxonomy
Fushman, et al[32]	2006	Answer extraction, semantic clustering, and extractive summarization for clinical question answering	Question answering system, summarization, frequently-occurring class of questions
Slaughter LA, et al[33]	2006	Semantic representation of consumer questions and physician answers	Question answering system, identification of semantic relationships
Yu, et al[34]	2006	The semantics of a definiendum constrains both the lexical semantics and the lexicosyntactic patterns in the definiens	Question answering system, identification of definitions,
Terol RM, et al[35]	2007	A knowledge based method for the medical question answering problem	Question answering system, domain knowledge source
Wang, et al[36]	2007	Automatic Clinical Question Answering Based on UMLS Relations	Question answering system, UMLS relations, concept identification, MetaMap
Sarrouti M, et al [37]	2017	A passage retrieval method based on probabilistic information retrieval model and UMLS concepts in biomedical question answering	Question answering system, UMLS for concept similarity

Kamath, et al[38]	2018	Verification of the Expected Answer Type for Biomedical Question Answering	Question answering system, answer extraction, NLP, deep learning
Ben Abacha A, et al [39]	2019	A question-entailment approach to question answering	Question answering system, UMLS to identify similar questions
**Other intelligent tools**			
Tolle, et al[40]	1999	UMLS-enhanced semantic parsing and personalized medical agent	UMLS-enhanced semantic parsing, personalized medical agent
Abidi, et al[41]	2000	Transforming XML-based electronic patient records for use in medical case based reasoning systems	Case-based reasoning systems, UMLS, medical knowledge ontologies, decision support
Manickam, et al[42]	2001	Extracting clinical cases from XML-based electronic patient records for use in web-based medical case based reasoning systems	Case-based reasoning, extraction of clinical cases
Jeffery, et al[43]	2009	CodeSlinger: An Interactive Biomedical Ontology Browser	Intelligent search and navigation tool of UMLS
Timsina, et al[44]	2016	Advanced analytics for the automation of medical systematic reviews	SVM as a classifier, automation of medical systematic review
Nawab, et al[45]	2017	An IR-Based Approach Utilizing Query Expansion for Plagiarism Detection in MEDLINE	Plagiarism detection, information retrieval, query expansion
Wang, et al[46]	2019	Automatic Human-like Mining and Constructing Reliable Genetic Association Database with Deep Reinforcement Learning	Text mining, artificial intelligent reader, automatic query

References:

1. Murphy, S.N. and G.O. Barnett, *Achieving automated narrative text interpretation using phrases in the electronic medical record.* Proc AMIA Annu Fall Symp, 1996: p. 532-6.
2. Eck, M., S. Vogel, and A. Waibel, *Improving statistical machine translation in the medical domain using the unified medical language system*, in *Proceedings of the 20th international conference on Computational Linguistics*. 2004, Association for Computational Linguistics: Geneva, Switzerland. p. 792–es.
3. Liu, K., et al., *Automating tissue bank annotation from pathology reports - comparison to a gold standard expert annotation set.* AMIA Annu Symp Proc, 2005: p. 460-4.
4. Matykiewicz, P., W. Duch, and J. Pestian, *Nonambiguous concept mapping in medical domain*, in *Proceedings of the 8th international conference on Artificial Intelligence and Soft Computing*. 2006, Springer-Verlag: Zakopane, Poland. p. 941–950.
5. French, L., et al., *Application and evaluation of automated semantic annotation of gene expression experiments.* Bioinformatics, 2009. **25**(12): p. 1543-9.

6. Gschwandtner, T., et al., *Easing semantically enriched information retrieval-An interactive semi-automatic annotation system for medical documents.* Int. J. Hum.-Comput. Stud., 2010. **68**(6): p. 370–385.
7. Kim, M.-Y., et al., *Unsupervised mapping of sentences to biomedical concepts based on integrated information retrieval model and clustering*, in *Proceedings of the First ACM International Conference on Bioinformatics and Computational Biology*. 2010, Association for Computing Machinery: Niagara Falls, New York. p. 322–329.
8. Rosenberg, K.M. and D.B. Coultas, *Acceptability of Unified Medical Language System terms as substitute for natural language general medicine clinic diagnoses.* Proc Annu Symp Comput Appl Med Care, 1994: p. 193-7.
9. Berman, J.J., *Resources for comparing the speed and performance of medical autocoders.* BMC Med Inform Decis Mak, 2004. **4**: p. 8.
10. Friedman, C., et al., *Automated encoding of clinical documents based on natural language processing.* J Am Med Inform Assoc, 2004. **11**(5): p. 392-402.
11. Erdal, S., et al., *ICD9 Code Assistant: A prototype.* AMIA Annu Symp Proc, 2007: p. 950.
12. Becker, B.F.H., et al., *CodeMapper: semiautomatic coding of case definitions. A contribution from the ADVANCE project.* Pharmacoepidemiol Drug Saf, 2017. **26**(8): p. 998-1005.
13. Helwe, C., et al., *CCS Coding of Discharge Diagnoses via Deep Neural Networks*, in *Proceedings of the 2017 International Conference on Digital Health*. 2017, Association for Computing Machinery: London, United Kingdom. p. 175–179.
14. Varghese, J., S. Sandmann, and M. Dugas, *Web-Based Information Infrastructure Increases the Interrater Reliability of Medical Coders: Quasi-Experimental Study.* J Med Internet Res, 2018. **20**(10): p. e274.
15. Whalen, G., *Medical textbook summarization and guided navigation using statistical sentence extraction.* AMIA Annu Symp Proc, 2005: p. 814-8.
16. Chen, P. and R. Verma, *A Query-Based Medical Information Summarization System Using Ontology Knowledge*, in *Proceedings of the 19th IEEE Symposium on Computer-Based Medical Systems*. 2006, IEEE Computer Society. p. 37–42.
17. Reeve, L., H. Han, and A.D. Brooks, *BioChain: lexical chaining methods for biomedical text summarization*, in *Proceedings of the 2006 ACM symposium on Applied computing*. 2006, Association for Computing Machinery: Dijon, France. p. 180–184.
18. Park, H.K. and J. Choi, *Towards chronological summary of medical records.* AMIA Annu Symp Proc, 2007: p. 911.
19. Reeve, L.H., H. Han, and A.D. Brooks, *Biomedical text summarisation using concept chains.* Int. J. Data Min. Bioinformatics, 2007. **1**(4): p. 389–407.
20. Morales, L.P., A.D. Esteban, and P. Gervás, *Concept-graph based biomedical automatic summarization using ontologies*, in *Proceedings of the 3rd Textgraphs Workshop on Graph-Based Algorithms for Natural Language Processing*. 2008, Association for Computational Linguistics: Manchester, United Kingdom. p. 53–56.
21. Plaza, L., M. Stevenson, and A. Díaz, *Improving summarization of biomedical documents using word sense disambiguation*, in *Proceedings of the 2010 Workshop on Biomedical Natural Language Processing*. 2010, Association for Computational Linguistics: Uppsala, Sweden. p. 55–63.
22. Plaza, L., A. Diaz, and P. Gervas, *A semantic graph-based approach to biomedical summarisation.* Artif Intell Med, 2011. **53**(1): p. 1-14.
23. Plaza, L., et al., *Studying the correlation between different word sense disambiguation methods and summarization effectiveness in biomedical texts.* BMC Bioinformatics, 2011. **12**: p. 355.

24. Plaza, L., M. Stevenson, and A. Díaz, *Resolving ambiguity in biomedical text to improve summarization.* Inf. Process. Manage., 2012. **48**(4): p. 755–766.
25. Roy, R., et al., *A method to summarize Disease Based Temporal state of human organ using laboratory test data and UMLS knowledge*, in *Proceedings of the 2012 IEEE 12th International Conference on Bioinformatics & Bioengineering (BIBE)*. 2012, IEEE Computer Society. p. 13–18.
26. Moradi, M. and N. Ghadiri, *Quantifying the informativeness for biomedical literature summarization: An itemset mining method.* Comput Methods Programs Biomed, 2017. **146**: p. 77-89.
27. Nasr Azadani, M., N. Ghadiri, and E. Davoodijam, *Graph-based biomedical text summarization: An itemset mining and sentence clustering approach.* J Biomed Inform, 2018. **84**: p. 42-58.
28. Kim, G.-W. and D.-H. Lee, *Personalised health document summarisation exploiting Unified Medical Language System and topic-based clustering for mobile healthcare.* J. Inf. Sci., 2018. **44**(5): p. 619–643.
29. Moradi, M. and N. Ghadiri, *Different approaches for identifying important concepts in probabilistic biomedical text summarization.* Artif Intell Med, 2018. **84**: p. 101-116.
30. Delbecque, T., P. Jacquemart, and P. Zweigenbaum, *Indexing UMLS Semantic Types for Medical Question-Answering.* Stud Health Technol Inform, 2005. **116**: p. 805-10.
31. Wedgwood, J., *MQAF: a medical question-answering framework.* AMIA Annu Symp Proc, 2005: p. 1150.
32. Demner-Fushman, D. and J. Lin, *Answer extraction, semantic clustering, and extractive summarization for clinical question answering*, in *Proceedings of the 21st International Conference on Computational Linguistics and the 44th annual meeting of the Association for Computational Linguistics*. 2006, Association for Computational Linguistics: Sydney, Australia. p. 841–848.
33. Slaughter, L.A., D. Soergel, and T.C. Rindflesch, *Semantic representation of consumer questions and physician answers.* Int J Med Inform, 2006. **75**(7): p. 513-29.
34. Yu, H. and Y. Wei, *The semantics of a definiendum constrains both the lexical semantics and the lexicosyntactic patterns in the definiens*, in *Proceedings of the Workshop on Linking Natural Language Processing and Biology: Towards Deeper Biological Literature Analysis*. 2006, Association for Computational Linguistics: New York City, New York. p. 1–8.
35. Terol, R.M., P. Martínez-Barco, and M. Palomar, *A knowledge based method for the medical question answering problem.* Comput. Biol. Med., 2007. **37**(10): p. 1511–1521.
36. Weiming, W., et al., *Automatic Clinical Question Answering Based on UMLS Relations*, in *Proceedings of the Third International Conference on Semantics, Knowledge and Grid*. 2007, IEEE Computer Society. p. 495–498.
37. Sarrouti, M. and S. Ouatik El Alaoui, *A passage retrieval method based on probabilistic information retrieval model and UMLS concepts in biomedical question answering.* J Biomed Inform, 2017. **68**: p. 96-103.
38. Kamath, S., B. Grau, and Y. Ma, *Verification of the Expected Answer Type for Biomedical Question Answering*, in *Companion Proceedings of the The Web Conference 2018*. 2018, International World Wide Web Conferences Steering Committee: Lyon, France. p. 1093–1097.
39. Ben Abacha, A. and D. Demner-Fushman, *A question-entailment approach to question answering.* BMC Bioinformatics, 2019. **20**(1): p. 511.
40. Tolle, K.M., et al., *Medical information access for the new millennium: UMLS-enhanced semantic parsing and personalized medical agent*, in *Proceedings of the fourth ACM conference on Digital libraries*. 1999, Association for Computing Machinery: Berkeley, California, USA. p. 252–253.
41. Abidi, S.S. and S. Manickam, *Transforming XML-based electronic patient records for use in medical case based reasoning systems.* Stud Health Technol Inform, 2000. **77**: p. 709-13.

42. Manickam, S. and S.S. Abidi, *Extracting clinical cases from XML-based electronic patient records for use in web-based medical case based reasoning systems.* Stud Health Technol Inform, 2001. **84**(Pt 1): p. 643-7.
43. Painter, J.L. and N.L. Flowers, *CodeSlinger: An Interactive Biomedical Ontology Browser*, in *Proceedings of the 12th Conference on Artificial Intelligence in Medicine: Artificial Intelligence in Medicine*. 2009, Springer-Verlag: Verona, Italy. p. 260–264.
44. Timsina, P., J. Liu, and O. El-Gayar, *Advanced analytics for the automation of medical systematic reviews.* Information Systems Frontiers, 2016. **18**(2): p. 237–252.
45. Nawab, R.M.A., M. Stevenson, and P. Clough, *An IR-Based Approach Utilizing Query Expansion for Plagiarism Detection in MEDLINE.* IEEE/ACM Trans Comput Biol Bioinform, 2017. **14**(4): p. 796-804.
46. Wang, H., et al., *Automatic Human-like Mining and Constructing Reliable Genetic Association Database with Deep Reinforcement Learning.* Pac Symp Biocomput, 2019. **24**: p. 112-123.
