# Supplementary material for: The Unified Medical Language System at 30 Years and How It Is Used and Published: Systematic Review and Content Analysis
Source: JMIR Med Inform. 2021 Aug 27;9(8):e20675. doi: 10.2196/20675 (PMC8433943; doi:10.2196/20675)
Supplement: Multimedia Appendix 6 [file medinform_v9i8e20675_app6.pdf]

Multimedia Appendix 6. Publications on data mining, knowledge discovery, and text analysis using the Unified Medical Language System.

Author	Publication year	Title	What was UMLS used for?
Fu, et al[1]	1991	Estimating frequency of disease findings from combined hospital databases: a UMLS project	UMLS, frequency of disease findings, merged database, aggregation analysis
Cimino, et [2]al	1992	Analysis of physician questions in an ambulatory care setting	Physicians questions analysis, Metathesaurus, content and relationship
Pietrzyk[3]	1995	Free text analysis	UMLS, SNOMED III, conceptual graph formalism, information retrieval
Wilbur, et al[4]	1999	Analysis of biomedical text for chemical names: a comparison of three methods	Analysis of biomedical text for chemical names, UMLS, chemical nomenclature
Van Mulligen, et al[5]	2002	Research for research: tools for knowledge discovery and visualization	Knowledge discovery, visualization, research tools, UMLS semantic network
Hsieh, et al[6]	2004	Linguistic analysis: terms and phrases used by patients in e-mail messages to nurses	Linguistic analysis, patients' email messages, NLP, MetaMap, mapping,
Hu, et al[7]	2005	Mining undiscovered public knowledge from complementary and non-interactive biomedical literature through semantic pruning	Literature mining, semantic pruning
Liang, et al[8]	2005	Anaphora resolution for biomedical literature by exploiting multiple resources	Verification of semantic association between anaphors and their antecedents, UMLS, WordNet, GENIA
Jang, et al[9]	2006	Text mining for medical documents using a hidden markov model	Semantic tagger, clinical documents, Hidden Markov Model (HMM), UMLS
Torii, et al[10]	2006	A comparison study of biomedical short form definition detection algorithms	Literature mining, detecting definitions for acronyms/abbreviations/symbols
Osborne, et al[11]	2007	Mining biomedical data using MetaMap Transfer (MMtx) and the Unified Medical Language System (UMLS)	MMTx, UMLS, data mining
Chung[12]	2009	Towards identifying intervention arms in randomized controlled trials: extracting coordinating constructions	Automated text mining, intervention identification, RCT, NLP, machine extraction

Sam, et al[13]	2009	PhenoGO: an integrated resource for the multiscale mining of clinical and biological data	PhenoGO, data mining, gene-disease annotation, phenotype, UMLS
Ijaz, et al[14]	2010	MKEM: a Multi-level Knowledge Emergence Model for mining undiscovered public knowledge	MKEM, knowledge discovery, implicit relationship extraction, MetaMap for tagging
Jarman, et al[15]	2010	Throw the bath water out, keep the baby: keeping medically-relevant terms for text mining	Text mining, term extraction, NLP, SNOMED CT, UMLS
Kilicoglu, et al[16]	2011	Constructing a semantic predication gold standard from the biomedical literature	Biomedical text mining, knowledge discovery, the gold standard to measure extraction quality, semantic prediction
Wilkowski, et al[17]	2011	Graph-based methods for discovery browsing with semantic predications	SemRep, literature-based discovery, semantic prediction
Yeganova, et al[18]	2011	Text mining techniques for leveraging positively labeled data	Text mining, labeled document, multiword UMLS phrases
Hong, et al[19]	2012	Analysis of RadLex Coverage and Term Co-occurrence in Radiology Reporting Templates	RadLex Coverage analysis, Term Co-occurrence, Radiology Reporting, reporting elements
Gabetta, et al[20]	2013	A Unified Medical Language System (UMLS) based system for Literature-Based Discovery in medicine	NLP, knowledge discovery from literature, UMLS mapping, dilated cardiomyopathies
Alroobi[21], et al	2014	Discovering dysregulated phenotype-related gene patterns	Discovery of dysregulated phenotype gene patterns, UMLS mapping
Eature Jimeno Yepes, et al[22]	2015	Knowledge based word-concept model estimation and refinement for biomedical text mining	Text mining, NLP, metamap, entity recognition, word sense disambiguation
MacKellar, et al[23]	2017	Conflict Discovery and Analysis for Clinical Trials	Cancer, locating and choosing clinical trials for treatment, shared decision making
Dalianis[24]	2018	Clinical Text Mining: Secondary Use of Electronic Patient Records	Text mining, NLP, machine learning, EMR
Peterson, et al[25]	2018	The Sublanguage of Clinical Problem Lists: A Corpus Analysis	Clinical problem list, corpus analysis, inferencing, reasoning

References:

1. Fu, L.S., et al., *Estimating frequency of disease findings from combined hospital databases: a UMLS project.* Proc Annu Symp Comput Appl Med Care, 1991: p. 373-7.
2. Cimino, C. and G.O. Barnett, *Analysis of physician questions in an ambulatory care setting.* Comput Biomed Res, 1992. **25**(4): p. 366-73.
3. Pietrzyk, P.M., *Free text analysis.* Int J Biomed Comput, 1995. **39**(1): p. 139-44.

4. Wilbur, W.J., et al., *Analysis of biomedical text for chemical names: a comparison of three methods.* Proc AMIA Symp, 1999: p. 176-80.
5. Van Mulligen, E.M., et al., *Research for research: tools for knowledge discovery and visualization.* Proc AMIA Symp, 2002: p. 835-9.
6. Hsieh, Y., G.A. Hardardottir, and P.F. Brennan, *Linguistic analysis: terms and phrases used by patients in e-mail messages to nurses.* Stud Health Technol Inform, 2004. **107**(Pt 1): p. 511-5.
7. Hu, X., et al., *Mining undiscovered public knowledge from complementary and non-interactive biomedical literature through semantic pruning*, in *Proceedings of the 14th ACM international conference on Information and knowledge management*. 2005, Association for Computing Machinery: Bremen, Germany. p. 249–250.
8. Liang, T. and Y.-H. Lin, *Anaphora resolution for biomedical literature by exploiting multiple resources*, in *Proceedings of the Second international joint conference on Natural Language Processing*. 2005, Springer-Verlag: Jeju Island, Korea. p. 742–753.
9. Jang, H., S.K. Song, and S.H. Myaeng, *Text mining for medical documents using a hidden markov model*, in *Proceedings of the Third Asia conference on Information Retrieval Technology*. 2006, Springer-Verlag: Singapore. p. 553–559.
10. Torii, M., et al., *A comparison study of biomedical short form definition detection algorithms*, in *Proceedings of the 1st international workshop on Text mining in bioinformatics*. 2006, Association for Computing Machinery: Arlington, Virginia, USA. p. 52–59.
11. Osborne, J.D., et al., *Mining biomedical data using MetaMap Transfer (MMtx) and the Unified Medical Language System (UMLS).* Methods Mol Biol, 2007. **408**: p. 153-69.
12. Chung, G.Y., *Towards identifying intervention arms in randomized controlled trials: extracting coordinating constructions.* J Biomed Inform, 2009. **42**(5): p. 790-800.
13. Sam, L.T., et al., *PhenoGO: an integrated resource for the multiscale mining of clinical and biological data.* BMC Bioinformatics, 2009. **10 Suppl 2**: p. S8.
14. Ijaz, A.Z., M. Song, and D. Lee, *MKEM: a Multi-level Knowledge Emergence Model for mining undiscovered public knowledge.* BMC Bioinformatics, 2010. **11 Suppl 2**: p. S3.
15. Jarman, J. and D.J. Berndt, *Throw the bath water out, keep the baby: keeping medically-relevant terms for text mining.* AMIA Annu Symp Proc, 2010. **2010**: p. 336-40.
16. Kilicoglu, H., et al., *Constructing a semantic predication gold standard from the biomedical literature.* BMC Bioinformatics, 2011. **12**: p. 486.
17. Wilkowski, B., et al., *Graph-based methods for discovery browsing with semantic predications.* AMIA Annu Symp Proc, 2011. **2011**: p. 1514-23.
18. Yeganova, L., et al., *Text mining techniques for leveraging positively labeled data*, in *Proceedings of BioNLP 2011 Workshop*. 2011, Association for Computational Linguistics: Portland, Oregon. p. 155–163.
19. Hong, Y., et al., *Analysis of RadLex coverage and term co-occurrence in radiology reporting templates.* J Digit Imaging, 2012. **25**(1): p. 56-62.
20. Gabetta, M., C. Larizza, and R. Bellazzi, *A Unified Medical Language System (UMLS) based system for Literature-Based Discovery in medicine.* Stud Health Technol Inform, 2013. **192**: p. 412-6.
21. Alroobi, R. and S. Salem, *Discovering dysregulated phenotype-related gene patterns*, in *Proceedings of the 5th ACM Conference on Bioinformatics, Computational Biology, and Health Informatics*. 2014, Association for Computing Machinery: Newport Beach, California. p. 524–532.
22. Jimeno Yepes, A. and R. Berlanga, *Knowledge based word-concept model estimation and refinement for biomedical text mining.* J Biomed Inform, 2015. **53**: p. 300-7.

23. MacKellar, B. and C. Schweikert, *Conflict Discovery and Analysis for Clinical Trials*, in *Proceedings of the 2017 International Conference on Digital Health*. 2017, Association for Computing Machinery: London, United Kingdom. p. 72–76.
24. Dalianis, H., *Clinical Text Mining: Secondary Use of Electronic Patient Records*. 2018: Springer Publishing Company, Incorporated.
25. Peterson, K.J. and H. Liu, *The Sublanguage of Clinical Problem Lists: A Corpus Analysis.* AMIA Annu Symp Proc, 2018. **2018**: p. 1451-1460.
