# Supplementary material for: The Unified Medical Language System at 30 Years and How It Is Used and Published: Systematic Review and Content Analysis
Source: JMIR Med Inform. 2021 Aug 27;9(8):e20675. doi: 10.2196/20675 (PMC8433943; doi:10.2196/20675)
Supplement: Multimedia Appendix 7 [file medinform_v9i8e20675_app7.pdf]

Multimedia Appendix 7. Unified Medical Language System publications related to information retrieval.

Author	Publication year	Title	What was UMLS used for?
**Image retrieval**			
Lowe, et al[1]	1998	Towards knowledge-based retrieval of medical images. The role of semantic indexing, image content representation and knowledge-based retrieval	Knowledge-based image retrieval, semantic indexing
Lowe, et al[2]	1999	Automated semantic indexing of imaging reports to support retrieval of medical images in the multimedia electronic medical record	Image retrieval, automated semantic indexing, imaging reports
Ruiz, et al[3]	2005	UB at CLEF 2005: bilingual CLIR and medical image retrieval tasks	Medical image retrieval, mapping using UMLS
Lacoste, et al[4]	2006	Inter-media concept-based medical image indexing and retrieval With UMLS at IPAL	Image retrieval, image indexing, concepts indexing
Racoceanu, et al[5]	2006	A semantic fusion approach between medical images and reports using UMLS	Medical image retrieval, semantic information indexing
Ruiz[6]	2006	Combining image features, case descriptions and UMLS concepts to improve retrieval of medical images	Medical image retrieval, MetaMap
Lacoste, et al[7]	2007	Medical-Image Retrieval Based on Knowledge-Assisted Text and Image Indexing	Medical image retrieval, image indexing, text indexing
Díaz-Galiano, et al[8]	2008	Query expansion on medical image retrieval: MeSH vs. UMLS	Medical image retrieval, query expansion
Kalpathy-Cramer, et al[9]	2008	Multimodal medical image retrieval OHSU at ImageCLEF 2008	Medical image retrieval, UMLS based query expansion
Lana-Serrano, et al[10]	2008	MIRACLE at ImageCLEFmed 2008: semantic vs. statistical strategies for topic expansion	MIRACLE research consortium, ImageCLEFmed task, MeSH, UMLS
Lim, et al[11]	2008	Bi-modal conceptual indexing for medical image retrieval	Image retrieval, automatic indexing, medical image databases
Maisonnasse, et al[12]	2008	LIG at ImageCLEF 2008	Image retrieval, UMLS knowledge resource

Miguel, et al[13]	2008	Evaluation of Automatically Assigned MeSH Terms for Retrieval of Medical Images	Medical image retrieval, automatically assign MeSH terms and UMLS concepts
Kahn, et al[14]	2009	Automated semantic indexing of figure captions to improve radiology image retrieval	radiology image retrieval, automatic semantic indexing, figure caption
Kim, et al[15]	2010	Caption-based topical descriptors for microscopic images as published in academic papers	microscopic images, caption-based topical descriptors
Chen, et al[16]	2012	Ontology-Guided Approach to Retrieving Disease Manifestation Images for Health Image Base Construction	Image retrieval, a comprehensive medical image database
Shore, et al[17]	2012	Integration of Imaging Signs into RadLex	Image retrieval, RadLex, Imaging Signs
Azcarate, et al[18]	2013	Improving image retrieval effectiveness via query expansion using MeSH hierarchical structure	image retrieval, UMLS concept identification
Chen, et al[19]	2013	Ontology-guided organ detection to retrieve web images of disease manifestation: towards the construction of a consumer-based health image library	Web images retrieval, consumer health image library, ontology-guided organ detection
Al-Safadi, et al[20]	2016	An overview and evaluation of the radiologists lounge, a semantic content-based radiographic images retrieval	Content-based radiographic images retrieval, image sharing
**Indexing**			
Chute, et al[21]	1991	Latent Semantic Indexing of medical diagnoses using UMLS semantic structures	Latent semantic indexing by UMLS, medical diagnoses
Wagner[22]	1991	An automatic indexing method for medical documents	Automated indexing of medical documents, MetaIndex, UMLS Meta-1
Satomura, et al[23]	1992	Automated diagnostic indexing by natural language processing	Automatic indexing by UMLS, discharge summaries
Wagner, et al[24]	1992	Evaluation of a Meta-1-based automatic indexing method for medical documents	Automatic indexing medical documents by UMLS
Yang, et al[25]	1993	Words or concepts: the features of indexing units and their optimal use in information retrieval	Information retrieval, canonical concepts indexing, UMLS
Berrios, et al[26]	1999	Knowledge requirements for automated inference of medical textbook markup	Automatic indexing, UMLS, automated inference of medical textbook markup

Lawrence, et al[27]	1999	Hierarchical concept indexing of full-text documents in the Unified Medical Language System information sources map	Concept indexing by UMLS, documents index
Ruch, et al[28]	1999	MEDTAG: tag-like semantics for medical document indexing	UMLS, semantic tagset, medical document indexing, MEDTAG
Berrios[29]	2000	Automated indexing for full text information retrieval	Automatic indexing, vector-space model
Hersh, et al[30]	2001	Selective automated indexing of findings and diagnoses in radiology reports	Automated indexing, radiology reports
McDonald, et al[31]	2001	UMLS concept indexing for production databases: a feasibility study	UMLS indexing, concepts identification in medical narrative texts
Mutalik, et al[32]	2001	Use of general-purpose negation detection to augment concept indexing of medical documents: a quantitative study using the UMLS	Negation detection, UMLS indexing, medical documents
Guo, et al[33]	2003	Knowledge-Enhanced Latent Semantic Indexing	Latent Semantic Indexing, vector space model
Huang, et al[34]	2003	A pilot study of contextual UMLS indexing to improve the precision of concept-based representation in XML-structured clinical radiology reports	Context-sensitive indexing by UMLS, clinical radiology reports
Zou, et al[35]	2003	IndexFinder: a method of extracting key concepts from clinical texts for indexing	Indexing clinical texts by UMLS
Aronson, et al[36]	2004	The NLM Indexing Initiative's Medical Text Indexer	Medical Text Indexer (MTI), MeSH indexing recommendations
Hishiki, et al[37]	2004	Indexing anatomical concepts to OMIM Clinical Synopsis using the UMLS Metathesaurus	Indexing anatomical concepts by UMLS, OMIM
Bashyam, et al[38]	2005	Indexing anatomical phrases in neuro-radiology reports to the UMLS 2005AA	Indexing anatomical phrases by UMLS, neuro-radiology reports
Cuggia, et al[39]	2005	Indexing method of digital audiovisual medical resources with semantic Web integration	Indexing digital audiovisual medical resources, MPEG-7, Dublin Core standard
Joubert, et al[40]	2005	Refinement of an automatic method for indexing medical literature--a preliminary study	Automatic indexing, UMLS, frequency of co-occurring
Humphrey, et al[41]	2006	Journal descriptor indexing tool for categorizing text according to discipline or semantic type	Journal descriptor indexing, text categorization

Zhang, et al[42]	2006	Developing a UMLS-based indexing tool for health science repository system	Automatic indexing by UMLS, institutional repository systems
Avillach, et al[43]	2007	A model for indexing medical documents combining statistical and symbolic knowledge	Medical documents indexing by UMLS
Berlanga-Llavori, et al[44]	2008	Conceptual Subtopic Identification in the Medical Domain	Subtopic identification, conceptual indexing of the retrieved documents
Joubert, et al[45]	2008	Using knowledge for indexing health web resources in a quality-controlled gateway	MeSH terms indexing, health web resource indexing
Le, et al[46]	2008	Using Bayesian Network for Conceptual Indexing: Application to Medical Document Indexing with UMLS Metathesaurus	UMLS meta-thesaurus, concept indexing, medical document indexing
Sneiderman, et al[47]	2008	UMLS-based automatic image indexing	Automatic image indexing, UMLS concepts identification
Cohen, et al[48]	2009	Predication-based semantic indexing: permutations as a means to encode predications in semantic space	Semantic indexing, SemRep, vector space model
Lowe, et al[49]	2009	Using a statistical natural language Parser augmented with the UMLS specialist lexicon to assign SNOMED CT codes to anatomic sites and pathologic diagnoses in full text pathology reports	Pathology report, automatic indexing, UMLS, SNOMED CT
Shah, et al[50]	2009	Ontology-driven indexing of public datasets for translational bioinformatics	Text annotation of gene expression, indexing by UMLS
Taylor, et al[51]	2010	Semantic Graph Based Document-Indexing Strategy for MEDLINE	UMLS Metathesaurus, WordNet, document indexing, Semantic Graph
Xiang, et al[52]	2012	k-Neighborhood decentralization: a comprehensive solution to index the UMLS for large scale knowledge discovery	UMLS indexing, knowledge discovery
Chebil, et al[53]	2013	BioDI: A New Approach to Improve Biomedical Documents Indexing	Biomedical documents indexing by UMLS, partial match
**Information retrieval**			
Powsner, et al[54]	1991	From patient reports to bibliographic retrieval: a Meta-1 front-end	Medline queries, patients report retrieval
Joubert, et al[55]	1993	A conceptual model for information retrieval with UMLS	Conceptual model, information retrieval, UMLS

Kingsland, et al[56]	1993	Coach: applying UMLS knowledge sources in an expert searcher environment	UMLS knowledge sources, MEDLINE search
Rindflesch, et al[57]	1993	Semantic processing in information retrieval	Information retrieval, UMLS
Yang, et al[25]	1993	Words or concepts: the features of indexing units and their optimal use in information retrieval	Information retrieval, canonical concepts, UMLS
Robert, et al[58]	1994	A computational model of information retrieval with UMLS	Computational model, information retrieval, UMLS
Aronson[59]	1996	The effect of textual variation on concept based information retrieval	Mapping biomedical texts to UMLS concepts, MetaMap
Suarez, et al[60]	1997	Searching for information on the Internet using the UMLS and Medical World Search	UMLS, information search, Medical World Search
Srinivasan, et al[61]	2001	Vocabulary mining for information retrieval: rough sets and fuzzy sets	Vocabulary mining, information retrieval
Mao, et al[62]	2002	Free-text medical document retrieval via phrase-based vector space model	Vector space model, conceptual similarity
Volk, et al[63]	2002	Semantic annotation for concept-based cross-language medical information retrieval	Semantic annotation, concept-based information retrieval
Zeng, et al[64]	2002	Characteristics of consumer terminology for health information retrieval	Consumer terminology, health information retrieval
Bartling, et al[65]	2003	Retrieval and classification of dental research articles	MeSH indexing for dental and craniofacial terms, UMLS
Auerbuch, et al[66]	2004	Context-sensitive medical information retrieval	Context-sensitive information retrieval
Gomez, et al[67]	2007	Efficient bibliographic searches on allergology using PubMed	Literature search, allergology, PubMed
Liu, et al[68]	2007	Knowledge-based query expansion to support scenario-specific retrieval of medical free text	Medical free text retrieval, knowledge-based query expansion
Mao, et al[69]	2007	The phrase-based vector space model for automatic retrieval of free-text medical documents	Document indexing scheme, phrase-based vector space model
Zhou[70]	2008	Knowledge-intensive conceptual retrieval of biomedical literature	UMLS, concept-based literature retrieval
Bhatt, et al[71]	2009	Ontology driven semantic profiling and retrieval in medical information systems	Ontology, retrieval in medical information systems
Shamdasani, et al[72]	2009	Semantic Matching Using the UMLS	Search Match algorithm, UMLS metathesaurus

Andrés Melgar S., et al[73]	2010	Knowledge retrieval in the anatomical domain	Knowledge retrieval, anatomical, UMLS
Plaza, et al[74]	2010	Retrieval of similar electronic health records using UMLS concept graphs	EHR retrieval, UMLS
Trieschnigg, et al[75]	2010	A cross-lingual framework for monolingual biomedical information retrieval	Biomedical information retrieval, cross-lingual
Andreasen, et al[76]	2011	A semantics-based approach to retrieving biomedical information	Information retrieval, formal ontology
Cohen, et al[77]	2011	CSI-OMIM--Clinical Synopsis Search in OMIM	OMIM phenotype search, tagging by UMLS and MeSH, similarity measures
Kitanovski, et al[78]	2013	Merging words and concepts for medical articles retrieval	Word-space approach, medical articles retrieval, Metamap
Choi, et al[79]	2014	Semantic concept-enriched dependence model for medical information retrieval	MetaMap, information retrieval
Shen, et al[80]	2015	Is Concept Mapping Useful for Biomedical Information Retrieval?	Concept mapping, information retrieval, UMLS
Tran, et al[81]	2015	Integrating association mining into relevance feedback for biomedical literature search	Association mining, relevance feedback, literature search, UMLS
Wang, et al[82]	2015	Concept-Based Relevance Models for Medical and Semantic Information Retrieval	Relevance model, semantic information retrieval, UMLS
Wang, et al[83]	2015	Retrieval of Semantically Similar Healthcare Questions in Healthcare Forums	Healthcare forums, Retrieving similar post, similar threads
Alonso, et al[84]	2016	Evaluation of semantic similarity metrics applied to the automatic retrieval of medical documents	Semantic similarity measures, automatic retrieval system
Ji, et al[85]	2016	Integrating unified medical language system and association mining techniques into relevance feedback for biomedical literature search	Literature retrieval, association mining
Zhu, et al[86]	2018	Expert and Student User Evaluation of Semantic Protocol Search	Intelligent semantic search, relevance feedback and measures
**Information retrieval system & search engine**			
Miller, et al[87]	1992	Prototyping an institutional IAIMS/UMLS information environment for an academic medical center	Link of IAIMS and UMLS, Information Management System
Paton, et al[88]	1992	Online bibliographic information: integration into an emerging IAIMS environment	UMLS, IAIMS, search expansion, literature search

McCray, et al[89]	2000	Design and implementation of a national clinical trials registry	Clinical trial registry
Boulos, et al[90]	2002	Towards a semantic medical Web: HealthCyberMap's tool for building an RDF metadata base of health information resources based on the Qualified Dublin Core Metadata Set	HealthCyberMap, RDF metadata base of health information resources, qualified Dublin Core Metadata, UMLS
Gaudinat, et al[91]	2004	WRAPIN: new generation health search engine using UMLS knowledge sources for MeSH term extraction from health documentation	Health search engine, MeSH term extraction, WRAPIN
Joubert, et al[92]	2005	Designing and implementing health data and information providers	Model of web portals, implement web portals, UMLS
Can, et al[93]	2007	MedicoPort: A medical search engine for all	Medical search engine, MedicoPort, UMLS provides domain knowledge,
Qashri, et al[94]	2017	Medical Specialists Retrieval System Using Unified Medical Language System	UMLS, medical specialists retrieval system
**Performance**			
Jachna, et al[95]	1993	Augmenting GRATEFUL MED with the UMLS Metathesaurus: an initial evaluation	UMLS, GRATEFUL MED, translation of clinical questions into effective searches
Divita, et al[96]	1998	Evaluating lexical variant generation to improve information retrieval	Lexical variant, information retrieval
Houston, et al[97]	2000	Exploring the use of concept spaces to improve medical information retrieval	Concept spaces, medical information retrieval
Rosebaum, et al[98]	2006	Analysis of medical student content searches that resulted in unidentified UMLS concepts	Failure analysis, UMLS, unmatched search string
Zhou, et al[99]	2006	Using concept-based indexing to improve language modeling approach to genomic IR	Genomic information retrieval, synonym and polysemy
Ding, et al[100]	2007	Augmenting Oracle Text with the UMLS for enhanced searching of free-text medical reports	UMLS, search of medical reports, information retrieval
Wen, et al[101]	2007	Ontology Based Clustering for Improving Genomic IR	Genomic information retrieval based on UMLS
Hong, et al[102]	2008	Use of consumer health vocabularies in online physician directory to improve physician search	Consumer health vocabulary, UMLS, physician directory search
Darmoni, et al[103]	2012	Improving information retrieval using Medical Subject Headings Concepts: a test case on rare and chronic diseases	Information retrieval, MeSH, rare and chronic diseases

Griffon, et al[104]	2012	Performance evaluation of Unified Medical Language SystemÂ®'s synonyms expansion to query PubMed	Synonyms expansion, UMLS, PubMed
Sondhi, et al[105]	2012	Leveraging medical thesauri and physician feedback for improving medical literature retrieval for case queries	Medical case-based literature retrieval
Babashzadeh, et al[106]	2013	Using semantic-based association rule mining for improving clinical text retrieval	Association rule (AR) mining, EMR, clincial text retrieval
Martinez, et al[107]	2014	Improving search over Electronic Health Records using UMLS-based query expansion through random walks	EHR search, automatic query expansion, UMLS
**Query**			
Cimino, et al[108]	1992	Interactive query workstation: a demonstration of the practical use of UMLS knowledge sources	Interactive queries, information retrieval, UMLS Knowledge Sources
Cimino, et al[109]	1993	Generic queries for meeting clinical information needs	Generic queries, clinical information needs
Johnson, et al[110]	1993	Interpreting natural language queries using the UMLS	UMLS, natural language queries
Peng, et al[111]	1993	Generating MEDLINE search strategies using a librarian knowledge-based system	Search formulation, MEDLINE, conceptual map, knowledge base
Joubert, et al[112]	1996	The project ARIANE: conceptual queries to information databases	ARIANE, conceptual queries
Aronson, et al[113]	1997	Query expansion using the UMLS Metathesaurus	UMLS-based query expansion, MetaMap
Joubert, et al[114]	1998	UMLS-based conceptual queries to biomedical information databases: an overview of the project ARIANE. Unified Medical Language System	UMLS-based conceptual queries, ARIANE
Hersh, et al[115]	2000	Assessing thesaurus-based query expansion using the UMLS Metathesaurus	Query expansion, UMLS Metathesaurus
Cucina, et al[116]	2001	Empirical formulation of a generic query set for clinical information retrieval systems	Generic query set, UMLS, real-world queries, clinical information retrieval
Chen, et al[117]	2003	HelpfulMed: intelligent searching for medical information over the internet	The automatically generated thesaurus, search for medical information
Plovnick, et al[118]	2004	Reformulation of consumer health queries with professional terminology: a pilot study	Query reformulation, UMLS mapping, consumer

Wollersheim, et al[119]	2005	Using Medical Test Collection Relevance Judgements to Identify Ontological Relationships Useful for Query Expansion	Query expansion evaluation framework (QEEF), UMLS
Radhouani, et al[120]	2006	Using External Knowledge to Solve Multi-Dimensional Queries	Multi-Dimensional Queries, indexing scheme
Leroy, et al[121]	2007	An end user evaluation of query formulation and results review tools in three medical meta-search engines	Query formulation, evaluation, medical meta-search engines
Thirion, et al[122]	2009	Optimization of the PubMed Automatic Term Mapping	PubMed query optimization, MeSH, automatic term mapping
Mu, et al[123]	2010	Towards effective genomic information retrieval: The impact of query complexity and expansion strategies	Query expansion strategies, UMLS, genomic information retrieval
Edwards, et al[124]	2010	MeSH represented MEDLINE query results	Functional Semantic Web, MeSH, UMLS

References

1. Lowe, H.J., et al., *Towards knowledge-based retrieval of medical images. The role of semantic indexing, image content representation and knowledge-based retrieval.* Proc AMIA Symp, 1998: p. 882-6.
2. Lowe, H.J., et al., *Automated semantic indexing of imaging reports to support retrieval of medical images in the multimedia electronic medical record.* Methods Inf Med, 1999. **38**(4-5): p. 303-7.
3. Ruiz, M.E. and S.B. Southwick, *UB at CLEF 2005: bilingual CLIR and medical image retrieval tasks*, in *Proceedings of the 6th international conference on Cross-Language Evalution Forum: accessing Multilingual Information Repositories*. 2005, Springer-Verlag: Vienna, Austria. p. 737–743.
4. Lacoste, C., et al., *Inter-media concept-based medical image indexing and retrieval With UMLS at IPAL*, in *Proceedings of the 7th international conference on Cross-Language Evaluation Forum: evaluation of multilingual and multi-modal information retrieval*. 2006, Springer-Verlag: Alicante, Spain. p. 694–701.
5. Racoceanu, D., et al., *A semantic fusion approach between medical images and reports using UMLS*, in *Proceedings of the Third Asia conference on Information Retrieval Technology*. 2006, Springer-Verlag: Singapore. p. 460–475.
6. Ruiz, M.E., *Combining image features, case descriptions and UMLS concepts to improve retrieval of medical images.* AMIA Annu Symp Proc, 2006: p. 674-8.
7. Lacoste, C., et al., *Medical-Image Retrieval Based on Knowledge-Assisted Text and Image Indexing.* IEEE Trans. Cir. and Sys. for Video Technol., 2007. **17**(7): p. 889–900.
8. Díaz-Galiano, M.C., et al., *Query expansion on medical image retrieval: MeSH vs. UMLS*, in *Proceedings of the 9th Cross-language evaluation forum conference on Evaluating systems for multilingual and multimodal information access*. 2008, Springer-Verlag: Aarhus, Denmark. p. 732–735.
9. Kalpathy-Cramer, J., et al., *Multimodal medical image retrieval OHSU at ImageCLEF 2008*, in *Proceedings of the 9th Cross-language evaluation forum conference on Evaluating systems for*

multilingual and multimodal information access. 2008, Springer-Verlag: Aarhus, Denmark. p. 744–751.

10. Lana-Serrano, S., J. Villena-Román, and J.C. González-Cristóbal, *MIRACLE at ImageCLEFmed 2008: semantic vs. statistical strategies for topic expansion*, in *Proceedings of the 9th Cross-language evaluation forum conference on Evaluating systems for multilingual and multimodal information access*. 2008, Springer-Verlag: Aarhus, Denmark. p. 719–723.
11. Lim, J.-H., et al., *Bi-modal conceptual indexing for medical image retrieval*, in *Proceedings of the 14th international conference on Advances in multimedia modeling*. 2008, Springer-Verlag: Kyoto, Japan. p. 456–465.
12. Maisonnasse, L., et al., *LIG at ImageCLEF 2008*, in *Proceedings of the 9th Cross-language evaluation forum conference on Evaluating systems for multilingual and multimodal information access*. 2008, Springer-Verlag: Aarhus, Denmark. p. 704–711.
13. Ruiz, M.E. and A. Névéol, *Evaluation of Automatically Assigned MeSH Terms for Retrieval of Medical Images*, in *Advances in Multilingual and Multimodal Information Retrieval: 8th Workshop of the Cross-Language Evaluation Forum, CLEF 2007, Budapest, Hungary, September 19-21, 2007, Revised Selected Papers*. 2008, Springer-Verlag. p. 641–648.
14. Kahn, C.E., Jr. and D.L. Rubin, *Automated semantic indexing of figure captions to improve radiology image retrieval.* J Am Med Inform Assoc, 2009. **16**(3): p. 380-6.
15. Kim, S., S. Lamkin, and P. Duncan, *Caption-based topical descriptors for microscopic images as published in academic papers.* Health Info Libr J, 2010. **27**(3): p. 235-43.
16. Chen, Y., et al., *Ontology-Guided Approach to Retrieving Disease Manifestation Images for Health Image Base Construction*, in *Proceedings of the 2012 IEEE Second International Conference on Healthcare Informatics, Imaging and Systems Biology*. 2012, IEEE Computer Society. p. 107.
17. Shore, M.W., D.L. Rubin, and C.E. Kahn, Jr., *Integration of imaging signs into RadLex.* J Digit Imaging, 2012. **25**(1): p. 50-5.
18. Crespo Azcarate, M., J. Mata Vazquez, and M. Mana Lopez, *Improving image retrieval effectiveness via query expansion using MeSH hierarchical structure.* J Am Med Inform Assoc, 2013. **20**(6): p. 1014-20.
19. Chen, Y., et al., *Ontology-guided organ detection to retrieve web images of disease manifestation: towards the construction of a consumer-based health image library.* J Am Med Inform Assoc, 2013. **20**(6): p. 1076-81.
20. Al-Safadi, L., R. Alomran, and F. Almutairi, *An overview and evaluation of the radiologists lounge, a semantic content-based radiographic images retrieval.* Multimedia Tools Appl., 2016. **75**(1): p. 607–625.
21. Chute, C.G., Y. Yang, and D.A. Evans, *Latent Semantic Indexing of medical diagnoses using UMLS semantic structures.* Proc Annu Symp Comput Appl Med Care, 1991: p. 185-9.
22. Wagner, M.M., *An automatic indexing method for medical documents.* Proc Annu Symp Comput Appl Med Care, 1991: p. 1011-7.
23. Satomura, Y. and M.B. do Amaral, *Automated diagnostic indexing by natural language processing.* Med Inform (Lond), 1992. **17**(3): p. 149-63.
24. Wagner, M.M. and G.F. Cooper, *Evaluation of a Meta-1-based automatic indexing method for medical documents.* Comput Biomed Res, 1992. **25**(4): p. 336-50.
25. Yang, Y. and C.G. Chute, *Words or concepts: the features of indexing units and their optimal use in information retrieval.* Proc Annu Symp Comput Appl Med Care, 1993: p. 685-9.
26. Berrios, D.C., A. Kehler, and L.M. Fagan, *Knowledge requirements for automated inference of medical textbook markup.* Proc AMIA Symp, 1999: p. 676-80.

27. Wright, L.W., et al., *Hierarchical concept indexing of full-text documents in the Unified Medical Language System information sources map.* J. Am. Soc. Inf. Sci., 1999. **50**(6): p. 514–523.
28. Ruch, P., et al., *MEDTAG: tag-like semantics for medical document indexing.* Proc AMIA Symp, 1999: p. 137-41.
29. Berrios, D.C., *Automated indexing for full text information retrieval.* Proc AMIA Symp, 2000: p. 71-5.
30. Hersh, W., et al., *Selective automated indexing of findings and diagnoses in radiology reports.* Comput. Biomed. Res., 2001. **34**(4): p. 262–273.
31. McDonald, F.S. and P.L. Elkin, *UMLS concept indexing for production databases: a feasibility study.* J Am Med Inform Assoc, 2001. **8**(5): p. 512-5.
32. Mutalik, P.G., A. Deshpande, and P.M. Nadkarni, *Use of general-purpose negation detection to augment concept indexing of medical documents: a quantitative study using the UMLS.* J Am Med Inform Assoc, 2001. **8**(6): p. 598-609.
33. Guo, D., et al., *Knowledge-Enhanced Latent Semantic Indexing.* Inf. Retr., 2003. **6**(2): p. 225–250.
34. Huang, Y., H.J. Lowe, and W.R. Hersh, *A pilot study of contextual UMLS indexing to improve the precision of concept-based representation in XML-structured clinical radiology reports.* J Am Med Inform Assoc, 2003. **10**(6): p. 580-7.
35. Zou, Q., et al., *IndexFinder: a method of extracting key concepts from clinical texts for indexing.* AMIA Annu Symp Proc, 2003: p. 763-7.
36. Aronson, A.R., et al., *The NLM Indexing Initiative's Medical Text Indexer.* Stud Health Technol Inform, 2004. **107**(Pt 1): p. 268-72.
37. Hishiki, T., et al., *Indexing anatomical concepts to OMIM Clinical Synopsis using the UMLS Metathesaurus.* In Silico Biol, 2004. **4**(1): p. 31-54.
38. Bashyam, V. and R.K. Taira, *Indexing anatomical phrases in neuro-radiology reports to the UMLS 2005AA.* AMIA Annu Symp Proc, 2005: p. 26-30.
39. Cuggia, M., F. Mougin, and P. Le Beux, *Indexing method of digital audiovisual medical resources with semantic Web integration.* Int J Med Inform, 2005. **74**(2-4): p. 169-77.
40. Joubert, M., et al., *Refinement of an automatic method for indexing medical literature--a preliminary study.* Stud Health Technol Inform, 2005. **116**: p. 683-8.
41. Humphrey, S.M., et al., *Journal descriptor indexing tool for categorizing text according to discipline or semantic type.* AMIA Annu Symp Proc, 2006: p. 960.
42. Zhang, D., et al., *Developing a UMLS-based indexing tool for health science repository system.* AMIA Annu Symp Proc, 2006: p. 1157.
43. Avillach, P., M. Joubert, and M. Fieschi, *A model for indexing medical documents combining statistical and symbolic knowledge.* AMIA Annu Symp Proc, 2007: p. 31-5.
44. Berlanga-Llavori, R., et al., *Conceptual Subtopic Identification in the Medical Domain*, in *Proceedings of the 11th Ibero-American conference on AI: Advances in Artificial Intelligence*. 2008, Springer-Verlag: Lisbon, Portugal. p. 312–321.
45. Joubert, M., et al., *Using knowledge for indexing health web resources in a quality-controlled gateway.* Stud Health Technol Inform, 2008. **136**: p. 205-10.
46. Le, T.H., J.-P. Chevallet, and J.H. Lim, *Using Bayesian Network for Conceptual Indexing: Application to Medical Document Indexing with UMLS Metathesaurus*, in *Advances in Multilingual and Multimodal Information Retrieval: 8th Workshop of the Cross-Language Evaluation Forum, CLEF 2007, Budapest, Hungary, September 19-21, 2007, Revised Selected Papers*. 2008, Springer-Verlag. p. 631–636.
47. Sneiderman, C., et al., *UMLS-based automatic image indexing.* AMIA Annu Symp Proc, 2008: p. 1141.

48. Cohen, T., R.W. Schvaneveldt, and T.C. Rindflesch, *Predication-based semantic indexing: permutations as a means to encode predications in semantic space.* AMIA Annu Symp Proc, 2009. **2009**: p. 114-8.
49. Lowe, H.J., Y. Huang, and D.P. Regula, *Using a statistical natural language Parser augmented with the UMLS specialist lexicon to assign SNOMED CT codes to anatomic sites and pathologic diagnoses in full text pathology reports.* AMIA Annu Symp Proc, 2009. **2009**: p. 386-90.
50. Shah, N.H., et al., *Ontology-driven indexing of public datasets for translational bioinformatics.* BMC Bioinformatics, 2009. **10 Suppl 2**: p. S1.
51. Taylor, W.P. and J.Z. Wang, *Semantic Graph Based Document-Indexing Strategy for MEDLINE*, in *Proceedings of the 2010 IEEE/WIC/ACM International Conference on Web Intelligence and Intelligent Agent Technology - Volume 01*. 2010, IEEE Computer Society. p. 287–290.
52. Xiang, Y., et al., *k-Neighborhood decentralization: a comprehensive solution to index the UMLS for large scale knowledge discovery.* J Biomed Inform, 2012. **45**(2): p. 323-36.
53. Chebil, W., L.F. Soualmia, and S.J. Darmoni, *BioDI: A New Approach to Improve Biomedical Documents Indexing*, in *Proceedings of the 24th International Conference on Database and Expert Systems Applications - Volume 8055*. 2013, Springer-Verlag: Prague, Czech Republic. p. 78–87.
54. Powsner, S.M. and P.L. Miller, *From patient reports to bibliographic retrieval: a Meta-1 front-end.* Proc Annu Symp Comput Appl Med Care, 1991: p. 526-30.
55. Joubert, M., M. Fieschi, and J.J. Robert, *A conceptual model for information retrieval with UMLS.* Proc Annu Symp Comput Appl Med Care, 1993: p. 715-9.
56. Kingsland, L.C., 3rd, et al., *Coach: applying UMLS knowledge sources in an expert searcher environment.* Bull Med Libr Assoc, 1993. **81**(2): p. 178-83.
57. Rindflesch, T.C. and A.R. Aronson, *Semantic processing in information retrieval.* Proc Annu Symp Comput Appl Med Care, 1993: p. 611-5.
58. Robert, J.J., et al., *A computational model of information retrieval with UMLS.* Proc Annu Symp Comput Appl Med Care, 1994: p. 167-71.
59. Aronson, A.R., *The effect of textual variation on concept based information retrieval.* Proc AMIA Annu Fall Symp, 1996: p. 373-7.
60. Suarez, H.H., X. Hao, and I.F. Chang, *Searching for information on the Internet using the UMLS and Medical World Search.* Proc AMIA Annu Fall Symp, 1997: p. 824-8.
61. Srinivasan, P., et al., *Vocabulary mining for information retrieval: rough sets and fuzzy sets.* Inf. Process. Manage., 2001. **37**(1): p. 15–38.
62. Mao, W. and W.W. Chu, *Free-text medical document retrieval via phrase-based vector space model.* Proc AMIA Symp, 2002: p. 489-93.
63. Volk, M., et al., *Semantic annotation for concept-based cross-language medical information retrieval.* Int J Med Inform, 2002. **67**(1-3): p. 97-112.
64. Zeng, Q., et al., *Characteristics of consumer terminology for health information retrieval.* Methods Inf Med, 2002. **41**(4): p. 289-98.
65. Bartling, W.C., T.K. Schleyer, and S. Visweswaran, *Retrieval and classification of dental research articles.* Adv Dent Res, 2003. **17**: p. 115-20.
66. Auerbuch, M., et al., *Context-sensitive medical information retrieval.* Stud Health Technol Inform, 2004. **107**(Pt 1): p. 282-6.
67. Saez Gomez, J.M., et al., *Efficient bibliographic searches on allergology using PubMed.* Allergol Immunopathol (Madr), 2007. **35**(6): p. 264-75.
68. Liu, Z. and W.W. Chu, *Knowledge-based query expansion to support scenario-specific retrieval of medical free text.* Inf. Retr., 2007. **10**(2): p. 173–202.

69. Mao, W. and W.W. Chu, *The phrase-based vector space model for automatic retrieval of free-text medical documents.* Data Knowl. Eng., 2007. **61**(1): p. 76–92.
70. Zhou, W., *Knowledge-intensive conceptual retrieval of biomedical literature*. 2008, University of Illinois at Chicago.
71. Bhatt, M., et al., *Ontology driven semantic profiling and retrieval in medical information systems.* Web Semant., 2009. **7**(4): p. 317–331.
72. Shamdasani, J., et al., *Semantic Matching Using the UMLS*, in *Proceedings of the 6th European Semantic Web Conference on The Semantic Web: Research and Applications*. 2009, Springer-Verlag: Heraklion, Crete, Greece. p. 203–217.
73. S., H.A.M., F.D. Beppler, and R.C.S. Pacheco, *Knowledge retrieval in the anatomical domain*, in *Proceedings of the 1st ACM International Health Informatics Symposium*. 2010, Association for Computing Machinery: Arlington, Virginia, USA. p. 684–693.
74. Plaza, L. and A. Díaz, *Retrieval of similar electronic health records using UMLS concept graphs*, in *Proceedings of the Natural language processing and information systems, and 15th international conference on Applications of natural language to information systems*. 2010, Springer-Verlag: Cardiff, UK. p. 296–303.
75. Trieschnigg, D., et al., *A cross-lingual framework for monolingual biomedical information retrieval*, in *Proceedings of the 19th ACM international conference on Information and knowledge management*. 2010, Association for Computing Machinery: Toronto, ON, Canada. p. 169–178.
76. Andreasen, T., et al., *A semantics-based approach to retrieving biomedical information*, in *Proceedings of the 9th international conference on Flexible Query Answering Systems*. 2011, Springer-Verlag: Ghent, Belgium. p. 108–118.
77. Cohen, R., et al., *CSI-OMIM--Clinical Synopsis Search in OMIM.* BMC Bioinformatics, 2011. **12**: p. 65.
78. Kitanovski, I., et al., *Merging words and concepts for medical articles retrieval*, in *Proceedings of the 10th Conference on Open Research Areas in Information Retrieval*. 2013, LE CENTRE DE HAUTES ETUDES INTERNATIONALES D'INFORMATIQUE DOCUMENTAIRE: Lisbon, Portugal. p. 25–28.
79. Choi, S., et al., *Semantic concept-enriched dependence model for medical information retrieval.* J Biomed Inform, 2014. **47**: p. 18-27.
80. Shen, W. and J.-Y. Nie, *Is Concept Mapping Useful for Biomedical Information Retrieval?*, in *Proceedings of the 6th International Conference on Experimental IR Meets Multilinguality, Multimodality, and Interaction - Volume 9283*. 2015, Springer-Verlag: Toulouse, France. p. 281–286.
81. Tran, J., P. Dews, and R.M. Massanari, *Integrating association mining into relevance feedback for biomedical literature search*, in *Proceedings of the 2015 IEEE International Conference on Bioinformatics and Biomedicine (BIBM)*. 2015, IEEE Computer Society Yanqing Ji Hao Ying. p. 531–536.
82. Wang, C. and R. Akella, *Concept-Based Relevance Models for Medical and Semantic Information Retrieval*, in *Proceedings of the 24th ACM International on Conference on Information and Knowledge Management*. 2015, Association for Computing Machinery: Melbourne, Australia. p. 173–182.
83. Wang, Y., et al., *Retrieval of Semantically Similar Healthcare Questions in Healthcare Forums*, in *Proceedings of the 2015 International Conference on Healthcare Informatics*. 2015, IEEE Computer Society. p. 517–518.
84. Alonso, I. and D. Contreras, *Evaluation of semantic similarity metrics applied to the automatic retrieval of medical documents.* Expert Syst. Appl., 2016. **44**(C): p. 386–399.

85. Ji, Y., et al., *Integrating unified medical language system and association mining techniques into relevance feedback for biomedical literature search.* BMC Bioinformatics, 2016. **17 Suppl 9**: p. 264.
86. Zhu, W., et al., *Expert and Student User Evaluation of Semantic Protocol Search*, in *Proceedings of the 2018 ACM International Conference on Bioinformatics, Computational Biology, and Health Informatics*. 2018, Association for Computing Machinery: Washington, DC, USA. p. 511–512.
87. Miller, P.L., et al., *Prototyping an institutional IAIMS/UMLS information environment for an academic medical center.* Bull Med Libr Assoc, 1992. **80**(3): p. 281-7.
88. Paton, J.A., et al., *Online bibliographic information: integration into an emerging IAIMS environment.* Proc Annu Symp Comput Appl Med Care, 1992: p. 605-9.
89. McCray, A.T. and N.C. Ide, *Design and implementation of a national clinical trials registry.* J Am Med Inform Assoc, 2000. **7**(3): p. 313-23.
90. Boulos, M.N., A.V. Roudsari, and E.R. Carson, *Towards a semantic medical Web: HealthCyberMap's tool for building an RDF metadata base of health information resources based on the Qualified Dublin Core Metadata Set.* Med Sci Monit, 2002. **8**(7): p. Mt124-36.
91. Gaudinat, A., et al., *WRAPIN: new generation health search engine using UMLS knowledge sources for MeSH term extraction from health documentation.* Stud Health Technol Inform, 2004. **107**(Pt 1): p. 356-60.
92. Joubert, M., et al., *Designing and implementing health data and information providers.* Int J Med Inform, 2005. **74**(2-4): p. 133-40.
93. Can, A.B. and N. Baykal, *MedicoPort: A medical search engine for all.* Comput. Methods Prog. Biomed., 2007. **86**(1): p. 73–86.
94. Qashri, A.Z., O. Karnalim, and H. Toba, *Medical Specialists Retrieval System Using Unified Medical Language System*, in *Proceedings of the 1st International Conference on Medical and Health Informatics 2017*. 2017, Association for Computing Machinery: Taichung City, Taiwan. p. 28–34.
95. Jachna, J.S., S.M. Powsner, and P.L. Miller, *Augmenting GRATEFUL MED with the UMLS Metathesaurus: an initial evaluation.* Bull Med Libr Assoc, 1993. **81**(1): p. 20-8.
96. Divita, G., A.C. Browne, and T.C. Rindflesch, *Evaluating lexical variant generation to improve information retrieval.* Proc AMIA Symp, 1998: p. 775-9.
97. Houston, A.L., et al., *Exploring the use of concept spaces to improve medical information retrieval.* Decis. Support Syst., 2000. **30**(2): p. 171–186.
98. Rosenbaum, B.P., J.C. Denny, and A. Spickard, 3rd, *Analysis of medical student content searches that resulted in unidentified UMLS concepts.* AMIA Annu Symp Proc, 2006: p. 1079.
99. Zhou, X., X. Zhang, and X. Hu, *Using concept-based indexing to improve language modeling approach to genomic IR*, in *Proceedings of the 28th European conference on Advances in Information Retrieval*. 2006, Springer-Verlag: London, UK. p. 444–455.
100. Ding, J., et al., *Augmenting Oracle Text with the UMLS for enhanced searching of free-text medical reports.* AMIA Annu Symp Proc, 2007: p. 940.
101. Wen, J., Z. Li, and X. Hu, *Ontology Based Clustering for Improving Genomic IR*, in *Proceedings of the Twentieth IEEE International Symposium on Computer-Based Medical Systems*. 2007, IEEE Computer Society. p. 225–230.
102. Hong, Y., R.D. Gillis, and R.F. Donnell, *Use of consumer health vocabularies in online physician directory to improve physician search.* AMIA Annu Symp Proc, 2008: p. 974.
103. Darmoni, S.J.M.D.P., et al., *Improving information retrieval using Medical Subject Headings Concepts: a test case on rare and chronic diseases.* Journal of the Medical Library Association, 2012. **100**(3): p. 176-83.

104. Griffon, N., et al., *Performance evaluation of Unified Medical Language System(R)'s synonyms expansion to query PubMed.* BMC Med Inform Decis Mak, 2012. **12**: p. 12.
105. Sondhi, P., et al., *Leveraging medical thesauri and physician feedback for improving medical literature retrieval for case queries.* J Am Med Inform Assoc, 2012. **19**(5): p. 851-8.
106. Babashzadeh, A., M. Daoud, and J. Huang, *Using semantic-based association rule mining for improving clinical text retrieval*, in *Proceedings of the second international conference on Health Information Science*. 2013, Springer-Verlag: London, UK. p. 186–197.
107. Martinez, D., et al., *Improving search over Electronic Health Records using UMLS-based query expansion through random walks.* J Biomed Inform, 2014. **51**: p. 100-6.
108. Cimino, C., et al., *Interactive query workstation: a demonstration of the practical use of UMLS knowledge sources.* Proc Annu Symp Comput Appl Med Care, 1992: p. 823-4.
109. Cimino, J.J., et al., *Generic queries for meeting clinical information needs.* Bull Med Libr Assoc, 1993. **81**(2): p. 195-206.
110. Johnson, S.B., et al., *Interpreting natural language queries using the UMLS.* Proc Annu Symp Comput Appl Med Care, 1993: p. 294-8.
111. Peng, P., et al., *Generating MEDLINE search strategies using a librarian knowledge-based system.* Proc Annu Symp Comput Appl Med Care, 1993: p. 596-600.
112. Joubert, M., et al., *The project ARIANE: conceptual queries to information databases.* Proc AMIA Annu Fall Symp, 1996: p. 378-82.
113. Aronson, A.R. and T.C. Rindflesch, *Query expansion using the UMLS Metathesaurus.* Proc AMIA Annu Fall Symp, 1997: p. 485-9.
114. Joubert, M., et al., *UMLS-based conceptual queries to biomedical information databases: an overview of the project ARIANE. Unified Medical Language System.* J Am Med Inform Assoc, 1998. **5**(1): p. 52-61.
115. Hersh, W., S. Price, and L. Donohoe, *Assessing thesaurus-based query expansion using the UMLS Metathesaurus.* Proc AMIA Symp, 2000: p. 344-8.
116. Cucina, R.J., et al., *Empirical formulation of a generic query set for clinical information retrieval systems.* Stud Health Technol Inform, 2001. **84**(Pt 1): p. 181-5.
117. Chen, H., et al., *HelpfulMed: intelligent searching for medical information over the internet.* J. Am. Soc. Inf. Sci. Technol., 2003. **54**(7): p. 683–694.
118. Plovnick, R.M. and Q.T. Zeng, *Reformulation of consumer health queries with professional terminology: a pilot study.* J Med Internet Res, 2004. **6**(3): p. e27.
119. Wollersheim, D. and W.J. Rahayu, *Using Medical Test Collection Relevance Judgements to Identify Ontological Relationships Useful for Query Expansion*, in *Proceedings of the 21st International Conference on Data Engineering Workshops*. 2005, IEEE Computer Society. p. 1160.
120. Radhouani, S. and G. Falquet, *Using External Knowledge to Solve Multi-Dimensional Queries*, in *Proceedings of the 2006 conference on Leading the Web in Concurrent Engineering: Next Generation Concurrent Engineering*. 2006, IOS Press. p. 426–437.
121. Leroy, G., et al., *An end user evaluation of query formulation and results review tools in three medical meta-search engines.* Int J Med Inform, 2007. **76**(11-12): p. 780-9.
122. Thirion, B., I. Robu, and S.J. Darmoni, *Optimization of the PubMed Automatic Term Mapping.* Stud Health Technol Inform, 2009. **150**: p. 238-42.
123. MU XM, L.K., *Towards effective genomic information retrieval: The impact of query complexity and expansion strategies.* J. Inf. Sci., 2010. **36**(2): p. 194–208.
124. Edwards, P. and V. Kešelj, *MeSH represented MEDLINE query results*, in *Proceedings of the 23rd Canadian conference on Advances in Artificial Intelligence*. 2010, Springer-Verlag: Ottawa, Canada. p. 75–86.
