# Supplementary material for: The Unified Medical Language System at 30 Years and How It Is Used and Published: Systematic Review and Content Analysis
Source: JMIR Med Inform. 2021 Aug 27;9(8):e20675. doi: 10.2196/20675 (PMC8433943; doi:10.2196/20675)
Supplement: Multimedia Appendix 8 [file medinform_v9i8e20675_app8.pdf]

Multimedia Appendix 8. Unified Medical Language System in publications related to natural language processing.

Author	Publication year	Title	What was UMLS used for?
**Abbreviation**			
Liu, et al[1]	2001	A study of abbreviations in the UMLS	UMLS, abbreviations
Liu, et al[2]	2002	A study of abbreviations in MEDLINE abstracts	Abbreviation ambiguity, textual variants of sense
Berman[3]	2004	Pathology Abbreviated: A Long Review of Short Terms	Pathology report, abbreviation, mapping to UMLS
Zhou, et al[4]	2006	ADAM: another database of abbreviations in MEDLINE	Abbreviation recognition in MEDLINE
Torii, et al[5]	2007	Enhancing acronym/abbreviation knowledge bases with semantic information	Acronym/abbreviation, automatic extraction from MEDLINE
Xu, et al[6]	2007	A study of abbreviations in clinical notes	Abbreviations in clinical notes, NLP, abbreviation database, sense inventory
Kim, et al[7]	2011	Using UMLS lexical resources to disambiguate abbreviations in clinical text	Clinical abbreviation disambiguation, acronyms disambiguation
McInnes, et al[8]	2011	Using second-order vectors in a knowledge-based method for acronym disambiguation	Disambiguate biomedical acronyms using second-order co-occurrence vectors
Kim, et al[9]	2013	Using Candidate Exploration and Ranking for Abbreviation Resolution in Clinical Document	Recognition of abbreviations, automatic text processing,
Moon, et al[10]	2014	A sense inventory for clinical abbreviations and acronyms created using clinical notes and medical dictionary resources	Clinical abbreviations, acronyms, UMLS mapping
Grossman, et al[11]	2018	A method for harmonization of clinical abbreviation and acronym sense inventories	Clinical abbreviation and acronym corpus, UMLS for evaluation
**Feature identification/extraction or phenotyping**			
Cheng, et al[12]	2011	Automated feature generation from structured knowledge	Features extraction, general learning tasks, YAGO, DBpedia, Freebase, UMLS
Adamusiak, et al[13]	2014	Next Generation Phenotyping Using the Unified Medical Language System	Phenotyping Using UMLS, extraction of features, EHR

Kauchak, et al[14]	2014	Text Simplification Tools: Using Machine Learning to Discover Features that Identify Difficult Text	Predicting the difficulty of health texts, medical concept density, text simplification
Ghosh, et al[15]	2019	Identifying Multi-dimensional Information from Microblogs During Epidemics	Feature identification, features from UMLS, syntactic and lexical structure of tweets, Weibo
**Lexicon/inventory**			
McCray, et al[16]	1994	Lexical methods for managing variation in biomedical terminologies	Variations in biomedical terminologies, UMLS
Tuttle, et al[17]	1996	Toward reusable software components at the point of care	UMLS based components, create lexical closure
Johnson, et al[18]	1999	A semantic lexicon for medical language processing	Automatic generation of a semantic lexicon, UMLS
Friedman, et al[19]	2001	Evaluating the UMLS as a source of lexical knowledge for medical language processing	UMLS evaluation, NLP, lexical knowledge
Verspoor[20]	2005	Towards a semantic lexicon for biological language processing: Conference Papers	UMLS, a lexicon for molecular biology
Hsiao, et al[21]	2009	Using UMLS to construct a generalized hierarchical concept-based dictionary of brain functions for information extraction from the fMRI literature	Dictionary, fMRI literature extraction, brain function
Liu, et al[22]	2012	Towards a semantic lexicon for clinical natural language processing	UMLS captures semantics, semantic lexicon
**Semantics**			
**Concept recognition/extraction**			
Nelson, et al[23]	1995	Identifying concepts in medical knowledge	Identification of concepts, mapping UMLS concepts
Srinivasan, et al[24]	2002	Finding UMLS Metathesaurus concepts in MEDLINE	UMLS concepts detection in MEDLINE, flexible matching
Brennan, et al[25]	2003	Towards linking patients and clinical information: detecting UMLS concepts in e-mail	UMLS concepts detection in emails, NLP, linking patients and clinical information
Happe, et al[26]	2003	Automatic concept extraction from spoken medical reports	Concept extraction, speech recognition, automated indexing
Shadow, et al[27]	2003	Extracting structured information from free text pathology reports	Structured information extraction, pathology reports, regular expressions
Brandt, et al[28]	2005	Automating identification of adverse events related to abnormal lab results using standard vocabularies	Mapping abnormal laboratory values to adverse events, UMLS, HL7/LOINC

Chung, et al[29]	2005	Concept-value pair extraction from semi-structured clinical narrative: a case study using echocardiogram reports	Concept extraction, echocardiogram reports,
Denny, et al[30]	2005	Identifying UMLS concepts from ECG Impressions using KnowledgeMap	UMLS concepts identification, ECG, UMLS mapping
Lieberman, et al[31]	2005	Using NLP to extract concepts from chief complaints	NLP, concept extraction, chief complaint
Long[32]	2005	Extracting diagnoses from discharge summaries	Diagnosis extraction, procedure extraction, discharge summary
Meystre, et al[33]	2005	Comparing natural language processing tools to extract medical problems from narrative text	NLP, MMTx, medical problem extraction
Meystre, et al[34]	2005	Evaluation of Medical Problem Extraction from Electronic Clinical Documents Using MetaMap Transfer (MMTx)	Medical problem extraction, MMTx, negation recognition
Slaughter, et al[35]	2005	Mapping cancer patients' symptoms to UMLS concepts	UMLS concept mapping, cancer, symptoms, physician coded problem list
Bashyam, et al[36]	2007	A normalized lexical lookup approach to identifying UMLS concepts in free text	UMLS concepts identification, MetaMap, radiology reports
Denny, et al[37]	2007	Identifying QT prolongation from ECG impressions using natural language processing and negation detection	NLP, negation detection, identifying QT prolongation
Lawrence, et al[38]	2007	CONANN: an online biomedical concept annotator	Biomedical concept annotator, CONANN, mapping source phrase to UMLS concepts
Cheng, et al[39]	2008	Extracting Alternative Splicing Information from Captions and Abstracts Using Natural Language Processing	MeSH to tag, captions and abstracts, Alternative splicing mechanisms, UMLS, NLP
Avillach, et al[40]	2009	A semantic approach for the homogeneous identification of events in eight patient databases: a contribution to the European eu-ADR project	Adverse drug events detection, queries across databases/languages
Bodenreider[41]	2009	Using SNOMED CT in combination with MedDRA for reporting signal detection and adverse drug reactions reporting	Adverse drug reactions detection, mapping between SNOMED CT and MedDRA via UMLS

Avillach, et al[42]	2010	Design and evaluation of a semantic approach for the homogeneous identification of events in eight patient databases: a contribution to the European EU-ADR project	Event identification across databases, UMLS concepts
Kaiser, et al[43]	2010	Identifying treatment activities for modeling computer-interpretable clinical practice guidelines	Treatment activities identification, clinical practice guidelines
Schritter, et al[44]	2010	Using domain knowledge about medications to correct recognition errors in medical report creation	Medication recognition, error recognition
Hazen, et al[45]	2011	Automatic Extraction of Concepts to Extend RadLex	Automatic Extraction of Concepts, Extend RadLex
Nebot, et al[46]	2011	Semantics-aware open information extraction in the biomedical domain	Information extraction, semantic annotation, UMLS as a knowledge source
Song, et al[47]	2011	Extracting biomedical concepts from fulltext by relative importance in a graph model	Concept extraction, named entity recognition and UMLS Semantic Network
Weng, et al[48]	2011	EliXR: an approach to eligibility criteria extraction and representation	Eligibility criteria extraction, clinical research, UMLS, EliXR
Wu, et al[49]	2011	Semantic characteristics of NLP-extracted concepts in clinical notes vs. biomedical literature	Concept extraction, MetaMap, domain similarity, qualitative measures, quantitative measures
Popescu, et al[50]	2012	Predicting health patterns using sensor sequence similarity and NLP	UMLS concepts extraction, long-term care, nursing notes, EHR
Avillach, et al[51]	2013	Harmonization process for the identification of medical events in eight European healthcare databases: the experience from the EU-ADR project	Harmonization and identification of medical events, extraction of potential adverse events
Kang, et al[52]	2013	Using rule-based natural language processing to improve disease normalization in biomedical text	NLP, disease normalization, MetaMap
Divita, et al[53]	2014	Sophia: A Expedient UMLS Concept Extraction Annotator	UMLS, concept extraction
Kang, et al[54]	2014	Knowledge-based extraction of adverse drug events from biomedical text	Adverse drug events extraction, relation extraction, concept recognition
Lopez-Garcia, et al[55]	2014	Cross-domain targeted ontology subsets for annotation: the case of SNOMED CORE and RxNorm	Drug extraction, problem list extraction, subset for annotation

Kors, et al[56]	2015	A multilingual gold-standard corpus for biomedical concept recognition: the Mantra GSC	Concept recognition corpus, , UMLS annotation, multilingual
Kate[57]	2016	Normalizing clinical terms using learned edit distance patterns	Normalize clinical terms to UMLS concepts
Lassoued, et al[58]	2016	Thesaurus-Based Hierarchical Semantic Grouping of Medical Terms in Information Extraction	Hierarchical semantic grouping, automatic extraction of risk information
Emadzadeh, et al[59]	2017	Hybrid Semantic Analysis for Mapping Adverse Drug Reaction Mentions in Tweets to Medical Terminology	Adverse drug reactions identification, UMLS is used as standard concepts, semantic mapping
Smith, et al[60]	2018	Methods to Compare Adverse Events in Twitter to FAERS, Drug Information Databases, and Systematic Reviews: Proof of Concept with Adalimumab	Compare Adverse Events in Twitter to FAERS, Adverse drug reactions
Torii, et al[61]	2018	A Preliminary Study of Clinical Concept Detection Using Syntactic Relations	Clinical concept detection, NLP
Tutubalina, et al[62]	2018	Medical concept normalization in social media posts with recurrent neural networks	Medical concept normalization, UMLS used as a standard thesaurus
Abbas, et al[63]	2019	Medical Concept Extraction using Smartphone and Natural Language Processing Techniques	Concept extraction, NLP, UMLS
Slovis, et al[64]	2019	Identifying Emergency Department Symptom-Based Diagnoses with the Unified Medical Language System	Symptom-Based Diagnoses identification, UMLS, emergency department
**Name entity recognition/extraction**			
Kirsch, et al[65]	2004	Distributed modules for text annotation and IE applied to the biomedical domain	Information extraction, GO, identification of gene and protein names, UniProt, UMLS,
Huang, et al[66]	2005	Improved identification of noun phrases in clinical radiology reports using a high-performance statistical natural language parser augmented with the UMLS specialist lexicon	Identification of noun phrases, radiology reports, sentence boundary detector,
Zeng, et al[67]	2005	Identifying consumer-friendly display (CFD) names for health concepts	Name entity recognition, consumer health terms
South, et al[68]	2007	Adaptation of the NegEx algorithm to Veterans Affairs electronic text notes for detection of influenza-like illness (ILI)	NegEx, detection of influenza-like illness

Taboada, et al[69]	2007	Using lexical, terminological and ontological resources for entity recognition tasks in the medical domain	Entity recognition, lexical, terminological and ontological resources
Jimeno, et al[70]	2008	Assessment of disease named entity recognition on a corpus of annotated sentences	Disease entity recognition, identification of association of genes to diseases
Segura-Bedmar, et al[71]	2008	A preliminary approach to recognize generic drug names by combining UMLS resources and USAN naming conventions	Drug name identification and classification, UMLS
Segura-Bedmar, et al[72]	2008	Drug name recognition and classification in biomedical texts. A case study outlining approaches underpinning automated systems	Drug name recognition, MMTx, automatic detection of drug interaction
Hettne, et al[73]	2009	A dictionary to identify small molecules and drugs in free text	Small molecules identification, drug identification, dictionary
Torri, et al[74]	2009	BioTagger-GM: a gene/protein name recognition system	BioTagger-GM, gene/protein name recognition system, UMLS
Khordad, et al[75]	2011	Improving phenotype name recognition	Named Entity Recognition, MetaMap, UMLS, Human Phenotype Ontology
Hajihashemi, et al[76]	2013	An early illness recognition framework using a temporal Smith Waterman algorithm and NLP	Illness recognition, MetaMap, health concepts extraction
Ghiasvand, et al[77]	2015	Biomedical Named Entity Recognition with less Supervision	Name entity recognition, clinical notes annotation
Kim, et al[78]	2015	Identifying named entities from PubMed for enriching semantic categories	Name entity recognition, NLP, semantic categories
Pradhan, et al[79]	2015	Evaluating the state of the art in disorder recognition and normalization of the clinical narrative	Disorder recognition, disorder normalization,
Reategui, et al[80]	2018	Comparison of MetaMap and cTAKES for entity extraction in clinical notes	Entity extraction, metaMap, cTakes, UMLS concepts
Wu, et al[81]	2018	Combine Factual Medical Knowledge and Distributed Word Representation to Improve Clinical Named Entity Recognition	Name entity recognition, word embeddings, unlabeled clinical corpus
Batbataar, et al[82]	2019	Ontology-Based Healthcare Named Entity Recognition from Twitter Messages Using a Recurrent Neural Network Approach	Name entity recognition, Twitter, Recurrent Neural Network,

**Natural language, vocabulary, question generation**			
Miller, et al[83]	2006	Dynamic generation of a table of contents with consumer-friendly labels	Generation of a table of contents, consumer health information, UMLS
Yu, et al[84]	2007	An automatic method to generate domain-specific investigator networks using PubMed abstracts	Investigator research profile, automatic generation, UMLS indexing
Cui, et al[85]	2014	A Semantic-based Approach for Exploring Consumer Health Questions Using UMLS	Consumer health questions, UMLS concepts tagging
**Natural language understanding**			
Mirhaji, et al[86]	2006	Semantic approach for text understanding of chief complaints data	Text understanding, chief complaint, concepts extraction, contextual information extraction
Sibanda, et al[87]	2006	Syntactically-informed semantic category recognition in discharge summaries	Semantic category recognition, discharge summaries, document understanding
Ruiz-Martinez, et al[88]	2011	Ontology learning from biomedical natural language documents using UMLS	Understanding of medical literature, UMLS, ontology
**Relationship recognition/extraction**			
Zeng, et al[89]	1998	Automated knowledge extraction from the UMLS	UMLS, disease-chemical relationship extraction, co-occurrence, disease-drug relationships
Brown, et al[90]	1999	Empirical derivation of an electronic clinically useful problem statement system	Relationship identification, problem list, evaluation against UMLS
Burgun, et al[91]	2001	Methods for exploring the semantics of the relationships between co-occurring UMLS concepts	UMLS, co-occurring, semantic categorization, semantic of the relationships
Hristoviski, et al[92]	2001	Supporting discovery in medicine by association rule mining in Medline and UMLS	Association rule mining, UMLS, interactive discovery support system
Charles, et al[93]	2004	A complex bio-networks of the function profile of genes	UMLS, GO, the association among co-occurring terms
Hu, et al[94]	2005	Mining novel connections from online biomedical text databases using semantic query expansion and semantic-relationship pruning	Novel connections identification from biomedical literature, query expansion
Bichindaritz[95]	2006	Named relationship mining from medical literature	Relationship identification, ConceptMiner, RelationshipMine

Butte, et al[96]	2006	Creation and implications of a phenome-genome network	Genotype-phenotype relationship identification, environmental, experimental contexts, UMLS
Hu, et al[97]	2006	A semi-supervised efficient learning approach to extract biological relationships from web-based biomedical digital library	Biological relationship extraction, semi-supervised efficient learning approach, UMLS
Ahlers, et al[98]	2007	Extracting semantic predications from Medline citations for pharmacogenomics	Pharmacogenomics, NLP, semantic prediction, entity co-occurrence
Hristovski, et al[99]	2007	Literature Based Discovery Support System and Its Application to Disease Gene Identification	Disease gene relationship identification, literature-based discovery system
Lin, et al[100]	2007	Methodologies for extracting functional pharmacogenomic experiments from international repository	Extraction of relationships between drugs and genes, pharmacogenomic
Chen, et al[101]	2008	Automated acquisition of disease drug knowledge from biomedical and clinical documents: an initial study	Drug-disease association identification, disease drug knowledge
Miller, et al[102]	2008	Visualization of health information with predications extracted using natural language processing and filtered using the UMLS	Prediction identification, phrase extraction, term identification visualization the propositions, UMLS
Ramakrishnan, et al[103]	2008	Unsupervised Discovery of Compound Entities for Relationship Extraction	Relationship extraction, compound entities identification
Yetisgen-Yildiz, et al[104]	2008	Finding the meaning of medical concept correlations	Semantic identification, relationship identification
Burgun, et al[105]	2009	Two approaches to integrating phenotype and clinical information	Identification of phenotype and clincial information, UMLS
Hristovski, et al[106]	2009	Semantic relations for interpreting DNA microarray data	SemRep, semantic relation extraction, computerized text analysis
Patel, et al[107]	2009	Using semantic and structural properties of the Unified Medical Language System to discover potential terminological relationships	UMLS, potential relationship discovery, semantic properties, structural properties
Segura-Bedmar, et al[108]	2009	DrugNerAR: linguistic rule-based anaphora resolver for drug-drug interaction extraction in pharmacological documents	Automatic drug-drug interaction extraction, DrugNerAR, drug anaphora resolution system

Segura-Bedmar, et al[109]	2009	Score-Based approach for anaphora resolution in drug-drug interactions documents	Drug-drug interactions extraction, parser
Segura-Bedmar, et al[110]	2010	Resolving anaphoras for the extraction of drug-drug interactions in pharmacological documents	Automatic drug-drug interaction extraction
Sebastian, et al[111]	2011	Domain-driven KDD for mining functionally novel rules and linking disjoint medical hypotheses	Association identification, semantic-based filtering based on UMLS
Okumura, et al[112]	2012	A lightweight approach for extracting disease-symptom relation with metamap toward automated generation of disease knowledge base	Disease-symptom relation extraction, automated generation of a knowledge base, MetaMap
Raghavan, et al[113]	2012	Medical event coreference resolution using the UMLS metathesaurus and temporal reasoning	Relation identification between medical events in the UMLS, the temporal relation between the medical events
Tymoshenko, et al[114]	2012	Relation mining in the biomedical domain using entity-level semantics	UMLS, relation mining between medical entities
Wojtusiak[115]	2012	Semantic Data Types in Machine Learning from Healthcare Data	Machine learning, extraction of semantics, UMLS, rule learning system
Abate, et al[116]	2013	Gelsius: A Literature-Based Workflow for Determining Quantitative Associations between Genes and Biological Processes	Quantitative association between genes and biological processes, UMLS
Abate, et al[117]	2013	Integration of Literature with Heterogeneous Information for Genes Correlation Scoring	Determining the correlation between biomedical terms, gene correlation
Camilo, et al[118]	2013	Process Fragment Recognition in Clinical Documents	Entity recognition, relationship recognition, clinical practice guidelines
Khare, et al[119]	2013	Toward Creating a Gold Standard of Drug Indications from FDA Drug Labels	Drug-disease relationships identification, candidate indications identification
Wenzina, et al[120]	2013	Identifying Condition-Action Sentences Using a Heuristic-Based Information Extraction Method	Identifying Condition-Action Sentences, computable clinical practice guidelines
Hanauer, et al[121]	2014	Applying MetaMap to Medline for identifying novel associations in a large clinical dataset: a feasibility analysis	Association identification, string similarity, UMLS-based query expansion
Vidal, et al[122]	2014	An authority-flow based ranking approach to discover potential	Semantic Web, Linking Open Data projects, association discovery

		novel associations between Linked Data	
Bakal, et al[123]	2015	Predicting Treatment Relations with Semantic Patterns over Biomedical Knowledge Graphs	Treatment relation identification, dataset of treatment relation from UMLS
Bejan, et al[124]	2015	Assessing the role of a medication-indication resource in the treatment relation extraction from clinical text	Identification of treatment relations, UMLS, medication-indication pair
Bornea, et al[125]	2015	Relational Path Mining in Structured Knowledge	Relation detection, corpus-based relation extractors
Chen, et al[126]	2015	Comparative analysis of a novel disease phenotype network based on clinical manifestations	UMLS, disease-manifestation identification
Ji, et al[127]	2015	High-Performance Biomedical Association Mining with MapReduce	MapReduce, biomedical association mining
Muzaffar, et al[128]	2015	A Relation Extraction Framework for Biomedical Text Using Hybrid Feature Set	Relation extraction, verb phrases ranking
Roberts, et al[129]	2015	Automatic Extraction and Post-coordination of Spatial Relations in Consumer Language	Concept recognition, spatial relationship extraction
Ji, et al[130]	2016	Leveraging MapReduce to efficiently extract associations between biomedical concepts from large text data	Association extraction, mapping texts to UMLS concepts, MapReduce
Cirincione, et al[131]	2018	Pathway networks generated from human disease phenome	Human disease phenome, genetic mutations and diseases relationship
Kumar, et al[132]	2018	Identifying Associations between Somatic Mutations and Clinicopathologic Findings in Lung Cancer Pathology Reports	Identification of associations between Somatic Mutations and Clinicopathologic Findings, lung cancer
Hua, et al[133]	2020	Health Effects Associated With Electronic Cigarette Use: Automated Mining of Online Forums	Text mining, health-effect data associated with e cigarettes, online forums, UMLS is used to annotate the posts, MataMap
**Semantic similarity/relatedness/distance**			
Caviedes, et al[134]	2004	Towards the development of a conceptual distance metric for the UMLS	Conceptual distance metric, UMLS, conceptual matching
Al-Mubaid, et al[135]	2006	A cluster-based approach for semantic similarity in the biomedical domain	Semantic similarity measures, distance measures

Al-Mubaid, et al[136]	2006	Using MEDLINE as Standard Corpus for Measuring Semantic Similarity in the Biomedical Domain	Semantic similarity, MEDLINE as standard corpus
Lavindrasana, et al[137]	2006	Knowledge acquisition for computation of semantic distance between WHO-ART terms	WHO-ART, semantic distance, knowledge acquisition
Fan, et al[138]	2007	Semantic classification of biomedical concepts using distributional similarity	Classification, validation, UMLS concepts, NLP, similarity measures
Al-Mubaid, et al[139]	2009	Measuring semantic similarity between biomedical concepts within multiple ontologies	Semantic similarity measures, MeSH, SNOMED CT, UMLS
McInnes, et al[140]	2009	UMLS-Interface and UMLS-Similarity : open source software for measuring paths and semantic similarity	Semantic similarity measures, path similarity, semantic similarity
Sanchez, et al[141]	2009	Computing Knowledge-Based Semantic Similarity from the Web: An Application to the Biomedical Domain	Semantic similarity computation, UMLS, knowledge-based
Genevieve, et al[142]	2010	Automated identification of synonyms in biomedical acronym sense inventories	Acronym disambiguation, semantic similarity algorithm
Allones, et al[143]	2012	A study of semantic proximity between archetype terms based on SNOMED CT relationships	Semantic proximity, OpenEHR archetypes, data model, interoperability, UMLS, mapping
Garla, et al[144]	2012	Semantic similarity in the biomedical domain: an evaluation across knowledge sources	Semantic similarity measures
Liu, et al[145]	2012	Semantic relatedness study using second order co-occurrence vectors computed from biomedical corpora, UMLS and WordNet	Measures of semantic relatedness, information retrieval
Sanchez, et al[146]	2012	Enabling semantic similarity estimation across multiple ontologies: an evaluation in the biomedical domain	Semantic similarity, MeSH, knowledge resources integration
Pasaranghader, et al[147]	2013	Adapting Gloss Vector Semantic Relatedness Measure for Semantic Similarity Estimation: An Evaluation in the Biomedical Domain	Automatic methods of ontology alignment, semantic similarity measures
Pesaranghader, et al[148]	2013	Applying Latent Semantic Analysis to Optimize Second-order Co-occurrence Vectors for Semantic Relatedness Measurement	Second-order Co-occurrence Vector semantic relatedness measure, UMLS

Pesaranghader, et al[149]	2013	Improving Gloss Vector Semantic Relatedness Measure by Integrating Pointwise Mutual Information: Optimizing Second-Order Co-occurrence Vectors Computed from Biomedical Corpus and UMLS	Gloss Vector semantic relatedness measure, information retrieval
Castro, et al[150]	2015	In the pursuit of a semantic similarity metric based on UMLS annotations for articles in PubMed Central Open Access	Semantic similarity metric, entity recognition, UMLS annotation
Ji, et al[151]	2017	Using ontology-based semantic similarity to facilitate the article screening process for systematic reviews	Article relationships (similarities) identification, concepts and concept relationships from UMLS
Lu, et al[152]	2017	Enhanced LexSynonym Acquisition for Effective UMLS Concept Mapping	Query expansion, semantic similarity, subterm substitutions, synonym thesaurus, concept mapping
Yu, et al[153]	2017	Retrofitting Concept Vector Representations of Medical Concepts to Improve Estimates of Semantic Similarity and Relatedness	UMLS, semantic similarity, semantic relatedness
**Word sense disambiguation**			
Liu, et al[154]	2001	Disambiguating ambiguous biomedical terms in biomedical narrative text: an unsupervised method	Disambiguation, biomedical narrative text, unsupervised method
Weeber, et al[155]	2001	Developing a test collection for biomedical word sense disambiguation	Word sense disambiguation, NLP, UMLS
Liu, et al[156]	2002	Automatic resolution of ambiguous terms based on machine learning and conceptual relations in the UMLS	Term disambiguation, machine learning, UMLS, sense disambiguation classifier
Liu, et al[157]	2002	Corpus-based ambiguity resolution of biomedical terms using knowledge bases and machine learning	NLP, word sense disambiguation, machine learning
Leroy, et al[158]	2004	Using symbolic knowledge in the UMLS to disambiguate words in small datasets with a naÃ¯ve Bayes classifier	Word sense disambiguation, symbolic knowledge, naïve byes classifier
Leroy, et al[159]	2005	Effects of information and machine learning algorithms on word sense disambiguation with small datasets	Word sense disambiguation, small datasets, symbolic knowledge
Humphrey, et al[160]	2006	Word sense disambiguation by selecting the best semantic type	Word sense disambiguation, Journal Descriptor Indexing,

		based on Journal Descriptor Indexing: Preliminary experiment	
McInnes, et al[161]	2007	Using UMLS Concept Unique Identifiers (CUIs) for word sense disambiguation in the biomedical domain	Word sense disambiguation, MetaMap, supervised learning approach
Tran, et al[162]	2007	Mapping terms to UMLS concepts of the same semantic type	Mapping, MetaMap, UMLS, word sense disambiguation
McInnes[163]	2008	An unsupervised vector approach to biomedical term disambiguation: integrating UMLS and Medline	Unsupervised vector approach, word sense disambiguation
Stevenson, et al[164]	2008	Disambiguation of biomedical text using diverse sources of information	MeSH, disambiguation
Stevenson, et al[165]	2008	Knowledge sources for word sense disambiguation of biomedical text	Word sense disambiguation, UMLS, NLP
Tan[166]	2008	Knowledge-based gene symbol disambiguation	Gene symbol disambiguation (GSD), UMLS
McInnes[167]	2009	Supervised and knowledge-based methods for disambiguating terms in biomedical text using the umls and metamap	Word sense disambiguation, automatic identification, UMLS, MetaMap
Agirre, et al[168]	2010	Graph-based word sense disambiguation of biomedical documents	Word sense disambiguation, automatic identification, biomedical documents
Patterson, et al[169]	2010	Automatic acquisition of sublanguage semantic schema: towards the word sense disambiguation of clinical narratives	Semantic schema, word sense disambiguation, MataMap, co-occurrence
Stevenson, et al[170]	2010	Disambiguation in the biomedical domain: the role of ambiguity type	Word sense disambiguation
Stevenson, et al[171]	2010	Disambiguation of ambiguous biomedical terms using examples generated from the UMLS Metathesaurus	Disambiguation, lexical ambiguity, UMLS concepts
Yepes, et al[172]	2010	Query Expansion for UMLS Metathesaurus Disambiguation Based on Automatic Corpus Extraction	Query expansion, UMLS, word sense disambiguation
Jimeno-Yepes, et al[173]	2011	Collocation analysis for UMLS knowledge-based word sense disambiguation	Word sense disambiguation, collocation analysis, UMLS
Jimeno-Yepes, et al[174]	2011	Exploiting MeSH indexing in MEDLINE to generate a data set for word sense disambiguation	Word sense disambiguation test collection, MEDLINE, MeSH
McInnes, et al[175]	2011	Knowledge-based method for determining the meaning of	Word sense disambiguation, semantic similarity measures,

		ambiguous biomedical terms using information content measures of similarity	semantic relatedness measures
Cheng, et al[176]	2012	Scaling up WSD with automatically generated examples	Word sense disambiguation (WSD), automatically labeled examples
Jimeno-Yepes, et al[177]	2012	Knowledge-based and knowledge-lean methods combined in unsupervised word sense disambiguation	Word sense disambiguation (WSD), information retrieval, information extraction
Stevenson, et al[178]	2012	Exploiting domain information for Word Sense Disambiguation of medical documents	Word sense disambiguation, topics of medical documents
El-Rab, et al[179]	2013	Biomedical text disambiguation using UMLS	Word sense disambiguation, UMLS, polysemous words
Garla, et al[180]	2013	Knowledge-based biomedical word sense disambiguation: an evaluation and application to clinical document classification	Word sense disambiguation, semantic similarity measures, clinical document classification
McInnes, et al[181]	2013	Evaluating measures of semantic similarity and relatedness to disambiguate terms in biomedical text	Word sense disambiguation, semantic similarity, semantic relatedness
McInnes, et al[182]	2014	Determining the difficulty of Word Sense Disambiguation	Word sense disambiguation, labeled training dataset
Rumshisky, et al[183]	2014	Word sense disambiguation in the clinical domain: a comparison of knowledge-rich and knowledge-poor unsupervised methods	Word sense disambiguation, similarity metrics
Festag, et al[184]	2017	Word Sense Disambiguation of Medical Terms via Recurrent Convolutional Neural Networks	Recurrent convolutional neural networks, word sense disambiguation,
Jimeno Yepes[185]	2017	Word embeddings and recurrent neural networks based on Long-Short Term Memory nodes in supervised biomedical word sense disambiguation	Word embeddings, supervised biomedical word sense disambiguation
Duque, et al[186]	2018	Co-occurrence graphs for word sense disambiguation in the biomedical domain	Word sense disambiguation, NLP, graph-based unsupervised technique
Pesaranghader, et al[187]	2019	deepBioWSD: effective deep neural word sense disambiguation of biomedical text data	Word sense disambiguation, deepBioWSD model
**Syntax**			
**Parsing**			
McCray[188]	1991	Extending a natural language parser with UMLS knowledge	UMLS, natural language parser, SPECIALIST,

Nelson et al[189]	2002	A semantic normal form for clinical drugs in the UMLS: early experiences with the VANDF	Clinical drugs, UMLS, drug vocabulary parse,
Nishimoto, et al[190]	2008	Development of a medical-text parsing algorithm based on character adjacent probability distribution for Japanese radiology reports	Japanese, radiology report, medical text parsing, MeSH, CT reports
Fan, et al[191]	2011	Deriving a probabilistic syntacto-semantic grammar for biomedicine based on domain-specific terminologies	Subset terminologies, syntactic parsing, syntactic structure
Taboada, et al[192]	2013	Combining open-source natural language processing tools to parse clinical practice guidelines	clinical practice guideline, UMLS, parsing
**Tagging**			
Mitchell, et al[193]	2004	Implementation and evaluation of a negation tagger in a pipeline-based system for information extract from pathology reports	Negation, tagging, information extraction, pathology report
Dozier, et al[194]	2007	Fast tagging of medical terms in legal text	Tagging medical terms in legal texts, UMLS
Liu, et al[195]	2012	Using SemRep to label semantic relations extracted from clinical text	SemRep, label semantic relations, clinical text
Chen, et al[196]	2017	The User Knows What to Call It: Incorporating Patient Voice Through User-Contributed Tags on a Participatory Platform About Health Management	Content analysis, tagging, automatic identification of UMLS concepts
Veytsman[197]	2019	How to measure the consistency of the tagging of scientific papers?	Consistency, tagging of scientific papers, indexing
**Terminology extraction**			
Hersh, et al[198]	1996	Empirical, automated vocabulary discovery using large text corpora and advanced natural language processing tools	Automated vocabulary discovery, coverage of UMLS
McCray, et al[199]	1998	Discovering the modifiers in a terminology data set	Large Scale Vocabulary Test, UMLS mapping, modifiers identification
Kim, et al[200]	2000	Corpus-based statistical screening for phrase identification	Phrase identification, statistical scoring methods
Travers, et al[201]	2004	Evaluation of emergency medical text processor, a system for cleaning chief complaint text data	Emergency department texts, chief complaints, UMLS, matching, terminology extraction

Wermter, et al[202]	2005	Effective grading of termhood in biomedical literature	Automatic terminology extraction, term identification measures
Ngomo[203]	2008	SIGNUM: a graph algorithm for terminology extraction	Terminology extraction, graph algorithm, UMLS
Luo, et al[204]	2013	A human-computer collaborative approach to identifying common data elements in clinical trial eligibility criteria	Common data elements identification, clinical trial eligibility criteria, UMLS term identification
Bentounsi, et al[205]	2015	Disambiguation of semantic types in complex noun phrases for extracting candidate terms	Term extraction, automatic document processing
**Text classification**			
Ruiz, et al[206]	2001	Combining machine learning and hierarchical structures for text categorization	Text categorization, hierarchical classification structures, UMLS
Alecu, et al[207]	2006	Mapping of the WHO-ART terminology on Snomed CT to improve grouping of related adverse drug reactions	Adverse drug reactions terms, SNOMED CT, UMLS, automatic classification, grouping
Aseervatham, et al[208]	2007	A Semantic Kernel for Semi-structured Document	SVM classifier, machine learning, text categorization task, medical documents
Aseervatham, et al[209]	2009	Semi-structured document categorization with a semantic kernel	Document categorization, machine learning, UMLS
Yi, et al[210]	2009	A hidden Markov model-based text classification of medical documents	Hidden Markov model, medical text classification, UMLS
Luo, et al[211]	2010	Semi-Automatically Inducing Semantic Classes of Clinical Research Eligibility Criteria Using UMLS and Hierarchical Clustering	Clinical research eligibility criteria extraction, hierarchical clustering
Luo, et al[212]	2011	Dynamic categorization of clinical research eligibility criteria by hierarchical clustering	Automatic categorization, clinical trial eligibility criteria extraction
Albitar, et al[213]	2012	Conceptualization Effects on MEDLINE Documents Classification Using Rocchio Method	Supervised text classification method, a traditional text classification method
Albitar, et al[214]	2012	The impact of conceptualization on text classification	Text classification techniques, conceptualization
Garla, et al[215]	2012	Ontology-guided feature engineering for clinical text classification	Feature identification, text classification, semantic similarity measure
**Other NLP related publications**			

McCray, et al[216]	1993	UMLS knowledge for biomedical language processing	UMLS, SPECIALIST, UMLS Metathesaurus, Semantic Network, mapping
Klavans, et al[217]	2001	Evaluation of DEFINDER: a system to mine definitions from consumer-oriented medical text	DEFINDER, a rule-based NLP system, consumer-oriented full-text articles
McCray, et al[218]	2001	Evaluating UMLS strings for natural language processing	UMLS, statistical NLP
Rindflesch, et al[219]	2003	The interaction of domain knowledge and linguistic structure in natural language processing: interpreting hypernymic propositions in biomedical text	NLP, hypernymic proposition, syntactic analysis, linguistic structure, UMLS
Bernhardt, et al[220]	2005	Determining prominent subdomains in medicine	Automatic identification of subdomains in medicine, statistical NLP
Wellner, et al[221]	2005	Adaptive string similarity metrics for biomedical reference resolution	String similarity metrics, mapping, string to UMLS
Torii, et al[222]	2006	Headwords and suffixes in biomedical names	headwords and suffixes in UMLS, stemming
Danger, et al[223]	2010	A comparison of machine learning techniques for detection of drug target articles	Drug target classifier, machine learning methods, UMLS as resources
Lingren, et al[224]	2012	Pre-annotating Clinical Notes and Clinical Trial Announcements for Gold Standard Corpus Development: Evaluating the Impact on Annotation Speed and Potential Bias	Gold standards corpus development, clinicaltrials.gov, UMLS, clinical notes annotation
Wu, et al[225]	2012	Unified Medical Language System term occurrences in clinical notes: a large-scale corpus analysis	UMLS terms in clinical notes, corpus analysis
Rudniy, et al[226]	2014	Mapping biological entities using the longest approximately common prefix method	String matching, string similarity, mapping
Pandey, et al[227]	2017	Improving RNN with Attention and Embedding for Adverse Drug Reactions	EHR, narrative texts, adverse drug reactions, MIMIC II, UMLS, Word2Vec, GloVe, RNN
Chen, et al[228]	2018	Evaluating semantic relations in neural word embeddings with biomedical and general domain knowledge bases	Neural word embedding, semantic relations
Wang, et al[229]	2018	A comparison of word embeddings for the biomedical natural language processing	Word bedding, NLP
Zolnoori, et al[230]	2019	A systematic approach for developing a corpus of patient	Patient-reported adverse drug events, corpus development,

		reported adverse drug events: A case study for SSRI and SNRI medications	entity normalization, mapping to UMLS

References:

1. Liu, H., Y.A. Lussier, and C. Friedman, *A study of abbreviations in the UMLS.* Proc AMIA Symp, 2001: p. 393-7.
2. Liu, H., A.R. Aronson, and C. Friedman, *A study of abbreviations in MEDLINE abstracts.* Proc AMIA Symp, 2002: p. 464-8.
3. Berman, J.J., *Pathology Abbreviated: A Long Review of Short Terms.* Archives of Pathology & Laboratory Medicine, 2004. **128**(3): p. 347-52.
4. Zhou, W., V.I. Torvik, and N.R. Smalheiser, *ADAM: another database of abbreviations in MEDLINE.* Bioinformatics, 2006. **22**(22): p. 2813-8.
5. Torii, M. and H. Liu, *Enhancing acronym/abbreviation knowledge bases with semantic information.* AMIA Annu Symp Proc, 2007: p. 731-5.
6. Xu, H., P.D. Stetson, and C. Friedman, *A study of abbreviations in clinical notes.* AMIA Annu Symp Proc, 2007: p. 821-5.
7. Kim, Y., J. Hurdle, and S.M. Meystre, *Using UMLS lexical resources to disambiguate abbreviations in clinical text.* AMIA Annu Symp Proc, 2011. **2011**: p. 715-22.
8. McInnes, B.T., et al., *Using second-order vectors in a knowledge-based method for acronym disambiguation*, in *Proceedings of the Fifteenth Conference on Computational Natural Language Learning*. 2011, Association for Computational Linguistics: Portland, Oregon. p. 145–153.
9. Kim, J.-B., et al., *Using Candidate Exploration and Ranking for Abbreviation Resolution in Clinical Document*, in *Proceedings of the 2013 IEEE International Conference on Healthcare Informatics*. 2013, IEEE Computer Society. p. 317–326.
10. Moon, S., et al., *A sense inventory for clinical abbreviations and acronyms created using clinical notes and medical dictionary resources.* J Am Med Inform Assoc, 2014. **21**(2): p. 299-307.
11. Grossman, L.V., et al., *A method for harmonization of clinical abbreviation and acronym sense inventories.* J Biomed Inform, 2018. **88**: p. 62-69.
12. Cheng, W., et al., *Automated feature generation from structured knowledge*, in *Proceedings of the 20th ACM international conference on Information and knowledge management*. 2011, Association for Computing Machinery: Glasgow, Scotland, UK. p. 1395–1404.
13. Adamusiak, T., N. Shimoyama, and M. Shimoyama, *Next Generation Phenotyping Using the Unified Medical Language System.* Journal of Medical Internet Research, 2014. **16**(3).
14. Kauchak, D., et al., *Text Simplification Tools: Using Machine Learning to Discover Features that Identify Difficult Text*, in *Proceedings of the 2014 47th Hawaii International Conference on System Sciences*. 2014, IEEE Computer Society. p. 2616–2625.
15. Ghosh, S., et al., *Identifying Multi-dimensional Information from Microblogs During Epidemics*, in *Proceedings of the ACM India Joint International Conference on Data Science and Management of Data*. 2019, Association for Computing Machinery: Kolkata, India. p. 224–230.
16. McCray, A.T., S. Srinivasan, and A.C. Browne, *Lexical methods for managing variation in biomedical terminologies.* Proc Annu Symp Comput Appl Med Care, 1994: p. 235-9.
17. Tuttle, M.S., et al., *Toward reusable software components at the point of care.* Proc AMIA Annu Fall Symp, 1996: p. 150-4.
18. Johnson, S.B., *A semantic lexicon for medical language processing.* J Am Med Inform Assoc, 1999. **6**(3): p. 205-18.

19. Friedman, C., et al., *Evaluating the UMLS as a source of lexical knowledge for medical language processing.* Proc AMIA Symp, 2001: p. 189-93.
20. Verspoor, K., *Towards a semantic lexicon for biological language processing: Conference Papers.* Comp. Funct. Genomics, 2005. **6**(1–2): p. 61–66.
21. Hsiao, M.Y., C.C. Chen, and J.H. Chen, *Using UMLS to construct a generalized hierarchical concept-based dictionary of brain functions for information extraction from the fMRI literature.* J Biomed Inform, 2009. **42**(5): p. 912-22.
22. Liu, H., et al., *Towards a semantic lexicon for clinical natural language processing.* AMIA Annu Symp Proc, 2012. **2012**: p. 568-76.
23. Nelson, S.J., et al., *Identifying concepts in medical knowledge.* Medinfo, 1995. **8 Pt 1**: p. 33-6.
24. Srinivasan, S., et al., *Finding UMLS Metathesaurus concepts in MEDLINE.* Proc AMIA Symp, 2002: p. 727-31.
25. Brennan, P.F. and A.R. Aronson, *Towards linking patients and clinical information: detecting UMLS concepts in e-mail.* J Biomed Inform, 2003. **36**(4-5): p. 334-41.
26. Happe, A., et al., *Automatic concept extraction from spoken medical reports.* Int J Med Inform, 2003. **70**(2-3): p. 255-63.
27. Schadow, G. and C.J. McDonald, *Extracting structured information from free text pathology reports.* AMIA Annu Symp Proc, 2003: p. 584-8.
28. Brandt, C.A., C.C. Lu, and P.M. Nadkarni, *Automating identification of adverse events related to abnormal lab results using standard vocabularies.* AMIA Annu Symp Proc, 2005: p. 903.
29. Chung, J. and S. Murphy, *Concept-value pair extraction from semi-structured clinical narrative: a case study using echocardiogram reports.* AMIA Annu Symp Proc, 2005: p. 131-5.
30. Denny, J.C., et al., *Identifying UMLS concepts from ECG Impressions using KnowledgeMap.* AMIA Annu Symp Proc, 2005: p. 196-200.
31. Lieberman, M.I. and T.N. Ricciardi, *Using NLP to extract concepts from chief complaints.* AMIA Annu Symp Proc, 2005: p. 1029.
32. Long, W., *Extracting diagnoses from discharge summaries.* AMIA Annu Symp Proc, 2005: p. 470-4.
33. Meystre, S.M. and P.J. Haug, *Comparing natural language processing tools to extract medical problems from narrative text.* AMIA Annu Symp Proc, 2005: p. 525-9.
34. Meystre, S. and P.J. Haug, *Evaluation of Medical Problem Extraction from Electronic Clinical Documents Using MetaMap Transfer (MMTx).* Stud Health Technol Inform, 2005. **116**: p. 823-8.
35. Slaughter, L., C. Ruland, and A.K. Rotegard, *Mapping cancer patients' symptoms to UMLS concepts.* AMIA Annu Symp Proc, 2005: p. 699-703.
36. Bashyam, V., et al., *A normalized lexical lookup approach to identifying UMLS concepts in free text.* Stud Health Technol Inform, 2007. **129**(Pt 1): p. 545-9.
37. Denny, J.C. and J.F. Peterson, *Identifying QT prolongation from ECG impressions using natural language processing and negation detection.* Stud Health Technol Inform, 2007. **129**(Pt 2): p. 1283-8.
38. Reeve, L.H. and H. Han, *CONANN: an online biomedical concept annotator*, in *Proceedings of the 4th international conference on Data integration in the life sciences*. 2007, Springer-Verlag: Philadelphia, PA, USA. p. 264–279.
39. Cheng, C.Y., F.R. Hsu, and C.Y. Tang, *Extracting Alternative Splicing Information from Captions and Abstracts Using Natural Language Processing*, in *Proceedings of the 2008 IEEE International Conference on Sensor Networks, Ubiquitous, and Trustworthy Computing (sutc 2008)*. 2008, IEEE Computer Society. p. 436–438.

40. Avillach, P., et al., *A semantic approach for the homogeneous identification of events in eight patient databases: a contribution to the European eu-ADR project.* Stud Health Technol Inform, 2009. **150**: p. 190-4.
41. Bodenreider, O., *Using SNOMED CT in combination with MedDRA for reporting signal detection and adverse drug reactions reporting.* AMIA Annu Symp Proc, 2009. **2009**: p. 45-9.
42. Avillach, P., et al., *Design and evaluation of a semantic approach for the homogeneous identification of events in eight patient databases: a contribution to the European EU-ADR project.* Stud Health Technol Inform, 2010. **160**(Pt 2): p. 1085-9.
43. Kaiser, K., A. Seyfang, and S. Miksch, *Identifying treatment activities for modeling computer-interpretable clinical practice guidelines*, in *Proceedings of the ECAI 2010 conference on Knowledge representation for health-care*. 2010, Springer-Verlag: Lisbon, Portugal. p. 114–125.
44. Schreitter, S., et al., *Using domain knowledge about medications to correct recognition errors in medical report creation*, in *Proceedings of the NAACL HLT 2010 Second Louhi Workshop on Text and Data Mining of Health Documents*. 2010, Association for Computational Linguistics: Los Angeles, California. p. 22–28.
45. Hazen, R., et al., *Automatic Extraction of Concepts to Extend RadLex.* Journal of Digital Imaging, 2011. **24**(1): p. 165-9.
46. Nebot, V. and R. Berlanga, *Semantics-aware open information extraction in the biomedical domain*, in *Proceedings of the 4th International Workshop on Semantic Web Applications and Tools for the Life Sciences*. 2011, Association for Computing Machinery: London, United Kingdom. p. 84–91.
47. Song, M., et al., *Extracting biomedical concepts from fulltext by relative importance in a graph model*, in *Proceedings of the 2011 IEEE International Conference on Bioinformatics and Biomedicine Workshops*. 2011, IEEE Computer Society. p. 586–593.
48. Weng, C., et al., *EliXR: an approach to eligibility criteria extraction and representation.* J Am Med Inform Assoc, 2011. **18 Suppl 1**: p. i116-24.
49. Wu, S. and H. Liu, *Semantic characteristics of NLP-extracted concepts in clinical notes vs. biomedical literature.* AMIA Annu Symp Proc, 2011. **2011**: p. 1550-8.
50. Popescu, M. and Z. Hajihashemi, *Predicting health patterns using sensor sequence similarity and NLP*, in *Proceedings of the 2012 IEEE International Conference on Bioinformatics and Biomedicine Workshops (BIBMW)*. 2012, IEEE Computer Society. p. 948–950.
51. Avillach, P., et al., *Harmonization process for the identification of medical events in eight European healthcare databases: the experience from the EU-ADR project.* J Am Med Inform Assoc, 2013. **20**(1): p. 184-92.
52. Kang, N., et al., *Using rule-based natural language processing to improve disease normalization in biomedical text.* J Am Med Inform Assoc, 2013. **20**(5): p. 876-81.
53. Divita, G., et al., *Sophia: A Expedient UMLS Concept Extraction Annotator.* AMIA Annu Symp Proc, 2014. **2014**: p. 467-76.
54. Kang, N., et al., *Knowledge-based extraction of adverse drug events from biomedical text.* BMC Bioinformatics, 2014. **15**: p. 64.
55. Lopez-Garcia, P., et al., *Cross-domain targeted ontology subsets for annotation: the case of SNOMED CORE and RxNorm.* J Biomed Inform, 2014. **47**: p. 105-11.
56. Kors, J.A., et al., *A multilingual gold-standard corpus for biomedical concept recognition: the Mantra GSC.* J Am Med Inform Assoc, 2015. **22**(5): p. 948-56.
57. Kate, R.J., *Normalizing clinical terms using learned edit distance patterns.* J Am Med Inform Assoc, 2016. **23**(2): p. 380-6.
58. Lassoued, Y. and L. Deleris, *Thesaurus-Based Hierarchical Semantic Grouping of Medical Terms in Information Extraction.* Stud Health Technol Inform, 2016. **228**: p. 446-50.

59. Emadzadeh, E., et al., *Hybrid Semantic Analysis for Mapping Adverse Drug Reaction Mentions in Tweets to Medical Terminology.* AMIA Annu Symp Proc, 2017. **2017**: p. 679-688.
60. Smith, K., et al., *Methods to Compare Adverse Events in Twitter to FAERS, Drug Information Databases, and Systematic Reviews: Proof of Concept with Adalimumab: An International Journal of Medical Toxicology and Drug Experience An International Journal of Medical Toxicology and Drug Experience.* Drug Safety, 2018. **41**(12): p. 1397-1410.
61. Torii, M., E.W. Yang, and S. Doan, *A Preliminary Study of Clinical Concept Detection Using Syntactic Relations.* AMIA Annu Symp Proc, 2018. **2018**: p. 1028-1035.
62. Tutubalina, E., et al., *Medical concept normalization in social media posts with recurrent neural networks.* J Biomed Inform, 2018. **84**: p. 93-102.
63. Abbas, A., M.Z. Ansaar, and S. Lee, *Medical Concept Extraction using Smartphone and Natural Language Processing Techniques (poster)*, in *Proceedings of the 17th Annual International Conference on Mobile Systems, Applications, and Services*. 2019, Association for Computing Machinery: Seoul, Republic of Korea. p. 630–631.
64. Slovis, B.H., et al., *Identifying Emergency Department Symptom-Based Diagnoses with the Unified Medical Language System.* West J Emerg Med, 2019. **20**(6): p. 910-917.
65. Kirsch, H. and D. Rebholz-Schuhmann, *Distributed modules for text annotation and IE applied to the biomedical domain*, in *Proceedings of the International Joint Workshop on Natural Language Processing in Biomedicine and its Applications*. 2004, Association for Computational Linguistics: Geneva, Switzerland. p. 50–53.
66. Huang, Y., et al., *Improved identification of noun phrases in clinical radiology reports using a high-performance statistical natural language parser augmented with the UMLS specialist lexicon.* J Am Med Inform Assoc, 2005. **12**(3): p. 275-85.
67. Zeng, Q.T., et al., *Identifying consumer-friendly display (CFD) names for health concepts.* AMIA Annu Symp Proc, 2005: p. 859-63.
68. South, B.R., et al., *Adaptation of the NegEx algorithm to Veterans Affairs electronic text notes for detection of influenza-like illness (ILI).* AMIA Annu Symp Proc, 2007: p. 1118.
69. Taboada, M., et al., *Using lexical, terminological and ontological resources for entity recognition tasks in the medical domain*, in *Proceedings of the 2007 conference on Knowledge management for health care procedures*. 2007, Springer-Verlag: Amsterdam, The Netherlands. p. 21–31.
70. Jimeno, A., et al., *Assessment of disease named entity recognition on a corpus of annotated sentences.* BMC Bioinformatics, 2008. **9 Suppl 3**: p. S3.
71. Segura-Bedmar, I., P. Martínez, and D. Samy, *A preliminary approach to recognize generic drug names by combining UMLS resources and USAN naming conventions*, in *Proceedings of the Workshop on Current Trends in Biomedical Natural Language Processing*. 2008, Association for Computational Linguistics: Columbus, Ohio. p. 100–101.
72. Segura-Bedmar, I., P. Martinez, and M. Segura-Bedmar, *Drug name recognition and classification in biomedical texts. A case study outlining approaches underpinning automated systems.* Drug Discov Today, 2008. **13**(17-18): p. 816-23.
73. Hettne, K.M., et al., *A dictionary to identify small molecules and drugs in free text.* Bioinformatics, 2009. **25**(22): p. 2983-91.
74. Torii, M., et al., *BioTagger-GM: a gene/protein name recognition system.* J Am Med Inform Assoc, 2009. **16**(2): p. 247-55.
75. Khordad, M., R.E. Mercer, and P. Rogan, *Improving phenotype name recognition*, in *Proceedings of the 24th Canadian conference on Advances in artificial intelligence*. 2011, Springer-Verlag: St. John's, Canada. p. 246–257.
76. Hajihashemi, Z. and M. Popescu, *An early illness recognition framework using a temporal Smith Waterman algorithm and NLP.* AMIA Annu Symp Proc, 2013. **2013**: p. 548-57.

77. Ghiasvand, O. and R.J. Kate, *Biomedical Named Entity Recognition with less Supervision*, in *Proceedings of the 2015 International Conference on Healthcare Informatics*. 2015, IEEE Computer Society. p. 495.
78. Kim, S., Z. Lu, and W.J. Wilbur, *Identifying named entities from PubMed for enriching semantic categories.* BMC Bioinformatics, 2015. **16**: p. 57.
79. Pradhan, S., et al., *Evaluating the state of the art in disorder recognition and normalization of the clinical narrative.* J Am Med Inform Assoc, 2015. **22**(1): p. 143-54.
80. Reategui, R. and S. Ratte, *Comparison of MetaMap and cTAKES for entity extraction in clinical notes.* BMC Med Inform Decis Mak, 2018. **18**(Suppl 3): p. 74.
81. Wu, Y., et al., *Combine Factual Medical Knowledge and Distributed Word Representation to Improve Clinical Named Entity Recognition.* AMIA Annu Symp Proc, 2018. **2018**: p. 1110-1117.
82. Batbaatar, E. and K.H. Ryu, *Ontology-Based Healthcare Named Entity Recognition from Twitter Messages Using a Recurrent Neural Network Approach.* Int J Environ Res Public Health, 2019. **16**(19).
83. Miller, T., G. Leroy, and E. Wood, *Dynamic generation of a table of contents with consumer-friendly labels.* AMIA Annu Symp Proc, 2006: p. 559-63.
84. Yu, W., et al., *An automatic method to generate domain-specific investigator networks using PubMed abstracts.* BMC Med Inform Decis Mak, 2007. **7**: p. 17.
85. Cui, L., S. Tao, and G.Q. Zhang, *A Semantic-based Approach for Exploring Consumer Health Questions Using UMLS.* AMIA Annu Symp Proc, 2014. **2014**: p. 432-41.
86. Mirhaji, P., et al., *Semantic approach for text understanding of chief complaints data.* AMIA Annu Symp Proc, 2006: p. 1033.
87. Sibanda, T., et al., *Syntactically-informed semantic category recognition in discharge summaries.* AMIA Annu Symp Proc, 2006: p. 714-8.
88. Ruiz-Martínez, J.M., et al., *Ontology learning from biomedical natural language documents using UMLS.* Expert Syst. Appl., 2011. **38**(10): p. 12365–12378.
89. Zeng, Q. and J.J. Cimino, *Automated knowledge extraction from the UMLS.* Proc AMIA Symp, 1998: p. 568-72.
90. Brown, S.H., et al., *Empirical derivation of an electronic clinically useful problem statement system.* Ann Intern Med, 1999. **131**(2): p. 117-26.
91. Burgun, A. and O. Bodenreider, *Methods for exploring the semantics of the relationships between co-occurring UMLS concepts.* Stud Health Technol Inform, 2001. **84**(Pt 1): p. 171-5.
92. Hristovski, D., et al., *Supporting discovery in medicine by association rule mining in Medline and UMLS.* Stud Health Technol Inform, 2001. **84**(Pt 2): p. 1344-8.
93. Liu, C.C.H., et al., *A complex bio-networks of the function profile of genes*, in *Transactions on Computational Systems Biology V*. 2006, Springer-Verlag. p. 9–24.
94. Hu, X. and X. Xu, *Mining novel connections from online biomedical text databases using semantic query expansion and semantic-relationship pruning.* Int. J. Web Grid Serv., 2005. **1**(2): p. 222–239.
95. Bichindaritz, I., *Named relationship mining from medical literature*, in *Proceedings of the 6th Industrial Conference on Data Mining conference on Advances in Data Mining: applications in Medicine, Web Mining, Marketing, Image and Signal Mining*. 2006, Springer-Verlag: Leipzig, Germany. p. 64–75.
96. Butte, A.J. and I.S. Kohane, *Creation and implications of a phenome-genome network.* Nat Biotechnol, 2006. **24**(1): p. 55-62.
97. Hu, X., et al., *A semi-supervised efficient learning approach to extract biological relationships from web-based biomedical digital library.* Web Intelli. and Agent Sys., 2006. **4**(3): p. 327–339.

98. Ahlers, C.B., et al., *Extracting semantic predications from Medline citations for pharmacogenomics.* Pac Symp Biocomput, 2007: p. 209-20.
99. Hristovski, D., et al., *Literature Based Discovery Support System and Its Application to Disease Gene Identification*, in *Computational Discovery of Scientific Knowledge: Introduction, Techniques, and Applications in Environmental and Life Sciences*. 2007, Springer-Verlag. p. 307–326.
100. Lin, Y.A., et al., *Methodologies for extracting functional pharmacogenomic experiments from international repository.* AMIA Annu Symp Proc, 2007: p. 463-7.
101. Chen, E.S., et al., *Automated acquisition of disease drug knowledge from biomedical and clinical documents: an initial study.* J Am Med Inform Assoc, 2008. **15**(1): p. 87-98.
102. Miller, T. and G. Leroy, *Visualization of health information with predications extracted using natural language processing and filtered using the UMLS.* AMIA Annu Symp Proc, 2008: p. 1057.
103. Ramakrishnan, C., et al., *Unsupervised Discovery of Compound Entities for Relationship Extraction*, in *Proceedings of the 16th international conference on Knowledge Engineering: Practice and Patterns*. 2008, Springer-Verlag: Acitrezza, Italy. p. 146–155.
104. Yetisgen-Yildiz, M. and W. Pratt, *Finding the meaning of medical concept correlations.* AMIA Annu Symp Proc, 2008: p. 830-4.
105. Burgun, A., F. Mougin, and O. Bodenreider, *Two approaches to integrating phenotype and clinical information.* AMIA Annu Symp Proc, 2009. **2009**: p. 75-9.
106. Hristovski, D., et al., *Semantic relations for interpreting DNA microarray data.* AMIA Annu Symp Proc, 2009. **2009**: p. 255-9.
107. Patel, C.O. and J.J. Cimino, *Using semantic and structural properties of the Unified Medical Language System to discover potential terminological relationships.* J Am Med Inform Assoc, 2009. **16**(3): p. 346-53.
108. Segura-Bedmar, I., et al., *DrugNerAR: linguistic rule-based anaphora resolver for drug-drug interaction extraction in pharmacological documents*, in *Proceedings of the third international workshop on Data and text mining in bioinformatics*. 2009, Association for Computing Machinery: Hong Kong, China. p. 19–26.
109. Segura-Bedmar, I., M. Crespo, and C.d. Pablo-Sánchez, *Score-Based approach for anaphora resolution in drug-drug interactions documents*, in *Proceedings of the 14th international conference on Applications of Natural Language to Information Systems*. 2009, Springer-Verlag: Saarbrücken, Germany. p. 91–102.
110. Segura-Bedmar, I., et al., *Resolving anaphoras for the extraction of drug-drug interactions in pharmacological documents.* BMC Bioinformatics, 2010. **11 Suppl 2**: p. S1.
111. Sebastian, Y. and P.H.H. Then, *Domain-driven KDD for mining functionally novel rules and linking disjoint medical hypotheses.* Know.-Based Syst., 2011. **24**(5): p. 609–620.
112. Okumura, T. and Y. Tateisi, *A lightweight approach for extracting disease-symptom relation with metamap toward automated generation of disease knowledge base*, in *Proceedings of the First international conference on Health Information Science*. 2012, Springer-Verlag: Beijing, China. p. 164–172.
113. Raghavan, P., et al., *Medical event coreference resolution using the UMLS metathesaurus and temporal reasoning*, in *Proceedings of the 2nd ACM SIGHIT International Health Informatics Symposium*. 2012, Association for Computing Machinery: Miami, Florida, USA. p. 465–472.
114. Tymoshenko, K., et al., *Relation mining in the biomedical domain using entity-level semantics*, in *Proceedings of the 20th European Conference on Artificial Intelligence*. 2012, IOS Press: Montpellier, France. p. 780–785.

115. Wojtusiak, J., *Semantic Data Types in Machine Learning from Healthcare Data*, in *Proceedings of the 2012 11th International Conference on Machine Learning and Applications - Volume 01*. 2012, IEEE Computer Society. p. 197–202.
116. Abate, F., et al., *Gelsius: A Literature-Based Workflow for Determining Quantitative Associations between Genes and Biological Processes.* IEEE/ACM Trans. Comput. Biol. Bioinformatics, 2013. **10**(3): p. 619–631.
117. Abate, F., et al., *Integration of Literature with Heterogeneous Information for Genes Correlation Scoring.* J. Emerg. Technol. Comput. Syst., 2013. **9**(4): p. Article 28.
118. Thorne, C., et al., *Process Fragment Recognition in Clinical Documents*, in *Proceeding of the XIIIth International Conference on AI*IA 2013: Advances in Artificial Intelligence - Volume 8249*. 2013, Springer-Verlag. p. 227–238.
119. Khare, R., J. Li, and Z. Lu, *Toward Creating a Gold Standard of Drug Indications from FDA Drug Labels*, in *Proceedings of the 2013 IEEE International Conference on Healthcare Informatics*. 2013, IEEE Computer Society. p. 30–35.
120. Wenzina, R. and K. Kaiser, *Identifying Condition-Action Sentences Using a Heuristic-Based Information Extraction Method*, in *Revised Selected Papers of the AIME 2013 Joint Workshop on Process Support and Knowledge Representation in Health Care - Volume 8268*. 2013, Springer-Verlag. p. 26–38.
121. Hanauer, D.A., et al., *Applying MetaMap to Medline for identifying novel associations in a large clinical dataset: a feasibility analysis.* J Am Med Inform Assoc, 2014. **21**(5): p. 925-37.
122. Vidal, M.-E., et al., *An authority-flow based ranking approach to discover potential novel associations between Linked Data.* Semant. web, 2014. **5**(1): p. 23–46.
123. Bakal, G. and R. Kavuluru, *Predicting Treatment Relations with Semantic Patterns over Biomedical Knowledge Graphs*, in *Proceedings of the Third International Conference on Mining Intelligence and Knowledge Exploration - Volume 9468*. 2015, Springer-Verlag: Hyderabad, India. p. 586–596.
124. Bejan, C.A., W.Q. Wei, and J.C. Denny, *Assessing the role of a medication-indication resource in the treatment relation extraction from clinical text.* J Am Med Inform Assoc, 2015. **22**(e1): p. e162-76.
125. Bornea, M. and K. Barker, *Relational Path Mining in Structured Knowledge*, in *Proceedings of the 8th International Conference on Knowledge Capture*. 2015, Association for Computing Machinery: Palisades, NY, USA. p. Article 14.
126. Chen, Y., et al., *Comparative analysis of a novel disease phenotype network based on clinical manifestations.* J Biomed Inform, 2015. **53**: p. 113-20.
127. Ji, Y., et al., *High-Performance Biomedical Association Mining with MapReduce*, in *Proceedings of the 2015 12th International Conference on Information Technology - New Generations*. 2015, IEEE Computer Society. p. 465–470.
128. Muzaffar, A.W., F. Azam, and U. Qamar, *A Relation Extraction Framework for Biomedical Text Using Hybrid Feature Set.* Comput Math Methods Med, 2015. **2015**: p. 910423.
129. Roberts, K., et al., *Automatic Extraction and Post-coordination of Spatial Relations in Consumer Language.* AMIA Annu Symp Proc, 2015. **2015**: p. 1083-92.
130. Ji, Y., et al., *Leveraging MapReduce to efficiently extract associations between biomedical concepts from large text data.* Microprocess. Microsyst., 2016. **46**(PB): p. 202–210.
131. Cirincione, A.G., K.L. Clark, and M.G. Kann, *Pathway networks generated from human disease phenome.* BMC Med Genomics, 2018. **11**(Suppl 3): p. 75.
132. Kumar, N., et al., *Identifying Associations between Somatic Mutations and Clinicopathologic Findings in Lung Cancer Pathology Reports.* Methods Inf Med, 2018. **57**(1): p. 63-73.

133. Hua, M.B.A.B., et al., *Health Effects Associated With Electronic Cigarette Use: Automated Mining of Online Forums.* Journal of Medical Internet Research, 2020. **22**(1): p. 1.
134. Caviedes, J.E. and J.J. Cimino, *Towards the development of a conceptual distance metric for the UMLS.* J Biomed Inform, 2004. **37**(2): p. 77-85.
135. Al-Mubaid, H. and H.A. Nguyen, *A cluster-based approach for semantic similarity in the biomedical domain.* Conf Proc IEEE Eng Med Biol Soc, 2006. **2006**: p. 2713-7.
136. Al-Mubaid, H. and H.A. Nguyen, *Using MEDLINE as Standard Corpus for Measuring Semantic Similarity in the Biomedical Domain*, in *Proceedings of the Sixth IEEE Symposium on BionInformatics and BioEngineering*. 2006, IEEE Computer Society. p. 315–318.
137. Iavindrasana, J., C. Bousquet, and M.C. Jaulent, *Knowledge acquisition for computation of semantic distance between WHO-ART terms.* Stud Health Technol Inform, 2006. **124**: p. 839-44.
138. Fan, J.W. and C. Friedman, *Semantic classification of biomedical concepts using distributional similarity.* J Am Med Inform Assoc, 2007. **14**(4): p. 467-77.
139. Al-Mubaid, H. and H.A. Nguyen, *Measuring semantic similarity between biomedical concepts within multiple ontologies.* Trans. Sys. Man Cyber Part C, 2009. **39**(4): p. 389–398.
140. McInnes, B.T., T. Pedersen, and S.V. Pakhomov, *UMLS-Interface and UMLS-Similarity : open source software for measuring paths and semantic similarity.* AMIA Annu Symp Proc, 2009. **2009**: p. 431-5.
141. Sánchez, D., M. Batet, and A. Valls, *Computing Knowledge-Based Semantic Similarity from the Web: An Application to the Biomedical Domain*, in *Proceedings of the 3rd International Conference on Knowledge Science, Engineering and Management*. 2009, Springer-Verlag: Vienna, Austria. p. 17–28.
142. Melton, G.B., et al., *Automated identification of synonyms in biomedical acronym sense inventories*, in *Proceedings of the NAACL HLT 2010 Second Louhi Workshop on Text and Data Mining of Health Documents*. 2010, Association for Computational Linguistics: Los Angeles, California. p. 46–52.
143. Allones, J.L., et al., *A study of semantic proximity between archetype terms based on SNOMED CT relationships*, in *Proceedings of the 2012 international conference on Process Support and Knowledge Representation in Health Care*. 2012, Springer-Verlag: Tallinn, Estonia. p. 98–112.
144. Garla, V.N. and C. Brandt, *Semantic similarity in the biomedical domain: an evaluation across knowledge sources.* BMC Bioinformatics, 2012. **13**: p. 261.
145. Liu, Y., et al., *Semantic relatedness study using second order co-occurrence vectors computed from biomedical corpora, UMLS and WordNet*, in *Proceedings of the 2nd ACM SIGHIT International Health Informatics Symposium*. 2012, Association for Computing Machinery: Miami, Florida, USA. p. 363–372.
146. Sanchez, D., et al., *Enabling semantic similarity estimation across multiple ontologies: an evaluation in the biomedical domain.* J Biomed Inform, 2012. **45**(1): p. 141-55.
147. Pesaranghader, A., A. Rezaei, and A. Pesaranghader, *Adapting Gloss Vector Semantic Relatedness Measure for Semantic Similarity Estimation: An Evaluation in the Biomedical Domain*, in *Revised Selected Papers of the Third Joint International Conference on Semantic Technology - Volume 8388*. 2013, Springer-Verlag: Seoul, South Korea. p. 129–145.
148. Pesaranghader, A., A. Pesaranghader, and A. Rezaei, *Applying Latent Semantic Analysis to Optimize Second-order Co-occurrence Vectors for Semantic Relatedness Measurement*, in *Proceedings of the First International Conference on Mining Intelligence and Knowledge Exploration - Volume 8284*. 2013, Springer-Verlag: Tamil Nadu, India. p. 588–599.
149. Pesaranghader, A., S. Muthaiyah, and A. Pesaranghader, *Improving Gloss Vector Semantic Relatedness Measure by Integrating Pointwise Mutual Information: Optimizing Second-Order Co-occurrence Vectors Computed from Biomedical Corpus and UMLS*, in *Proceedings of the 2013*

International Conference on Informatics and Creative Multimedia. 2013, IEEE Computer Society. p. 196–201.
150. Garcia Castro, L.J., R. Berlanga, and A. Garcia, *In the pursuit of a semantic similarity metric based on UMLS annotations for articles in PubMed Central Open Access.* J Biomed Inform, 2015. **57**: p. 204-18.
151. Ji, X., A. Ritter, and P.Y. Yen, *Using ontology-based semantic similarity to facilitate the article screening process for systematic reviews.* J Biomed Inform, 2017. **69**: p. 33-42.
152. Lu, C.J., et al., *Enhanced LexSynonym Acquisition for Effective UMLS Concept Mapping.* Stud Health Technol Inform, 2017. **245**: p. 501-505.
153. Yu, Z., et al., *Retrofitting Concept Vector Representations of Medical Concepts to Improve Estimates of Semantic Similarity and Relatedness.* Stud Health Technol Inform, 2017. **245**: p. 657-661.
154. Liu, H., Y.A. Lussier, and C. Friedman, *Disambiguating ambiguous biomedical terms in biomedical narrative text: an unsupervised method.* Comput. Biomed. Res., 2001. **34**(4): p. 249–261.
155. Weeber, M., J.G. Mork, and A.R. Aronson, *Developing a test collection for biomedical word sense disambiguation.* Proc AMIA Symp, 2001: p. 746-50.
156. Liu, H., S.B. Johnson, and C. Friedman, *Automatic resolution of ambiguous terms based on machine learning and conceptual relations in the UMLS.* J Am Med Inform Assoc, 2002. **9**(6): p. 621-36.
157. Liu, H., *Corpus-based ambiguity resolution of biomedical terms using knowledge bases and machine learning*. 2002, City University of New York.
158. Leroy, G. and T.C. Rindflesch, *Using symbolic knowledge in the UMLS to disambiguate words in small datasets with a naive Bayes classifier.* Stud Health Technol Inform, 2004. **107**(Pt 1): p. 381-5.
159. Leroy, G. and T.C. Rindflesch, *Effects of information and machine learning algorithms on word sense disambiguation with small datasets.* Int J Med Inform, 2005. **74**(7-8): p. 573-85.
160. Humphrey, S.M., et al., *Word sense disambiguation by selecting the best semantic type based on Journal Descriptor Indexing: Preliminary experiment.* J. Am. Soc. Inf. Sci. Technol., 2006. **57**(1): p. 96–113.
161. McInnes, B.T., T. Pedersen, and J. Carlis, *Using UMLS Concept Unique Identifiers (CUIs) for word sense disambiguation in the biomedical domain.* AMIA Annu Symp Proc, 2007: p. 533-7.
162. Tran, N., T. Luong, and M. Krauthammer, *Mapping terms to UMLS concepts of the same semantic type.* AMIA Annu Symp Proc, 2007: p. 1136.
163. McInnes, B.T., *An unsupervised vector approach to biomedical term disambiguation: integrating UMLS and Medline*, in *Proceedings of the 46th Annual Meeting of the Association for Computational Linguistics on Human Language Technologies: Student Research Workshop*. 2008, Association for Computational Linguistics: Columbus, Ohio. p. 49–54.
164. Stevenson, M., et al., *Disambiguation of biomedical text using diverse sources of information.* BMC Bioinformatics, 2008. **9 Suppl 11**: p. S7.
165. Stevenson, M., et al., *Knowledge sources for word sense disambiguation of biomedical text*, in *Proceedings of the Workshop on Current Trends in Biomedical Natural Language Processing*. 2008, Association for Computational Linguistics: Columbus, Ohio. p. 80–87.
166. Tan, H., *Knowledge-based gene symbol disambiguation*, in *Proceedings of the 2nd international workshop on Data and text mining in bioinformatics*. 2008, Association for Computing Machinery: Napa Valley, California, USA. p. 73–76.
167. Mcinnes, B.T., *Supervised and knowledge-based methods for disambiguating terms in biomedical text using the umls and metamap*. 2009, University of Minnesota.

168. Agirre, E., A. Soroa, and M. Stevenson, *Graph-based word sense disambiguation of biomedical documents.* Bioinformatics, 2010. **26**(22): p. 2889-96.
169. Patterson, O., S. Igo, and J.F. Hurdle, *Automatic acquisition of sublanguage semantic schema: towards the word sense disambiguation of clinical narratives.* AMIA Annu Symp Proc, 2010. **2010**: p. 612-6.
170. Stevenson, M. and Y. Guo, *Disambiguation in the biomedical domain: the role of ambiguity type.* J Biomed Inform, 2010. **43**(6): p. 972-81.
171. Stevenson, M. and Y. Guo, *Disambiguation of ambiguous biomedical terms using examples generated from the UMLS Metathesaurus.* J Biomed Inform, 2010. **43**(5): p. 762-73.
172. Yepes, A.J. and A.R. Aronson, *Query Expansion for UMLS Metathesaurus Disambiguation Based on Automatic Corpus Extraction*, in *Proceedings of the 2010 Ninth International Conference on Machine Learning and Applications*. 2010, IEEE Computer Society. p. 965–968.
173. Jimeno-Yepes, A., B.T. McInnes, and A.R. Aronson, *Collocation analysis for UMLS knowledge-based word sense disambiguation.* BMC Bioinformatics, 2011. **12 Suppl 3**: p. S4.
174. Jimeno-Yepes, A.J., B.T. McInnes, and A.R. Aronson, *Exploiting MeSH indexing in MEDLINE to generate a data set for word sense disambiguation.* BMC Bioinformatics, 2011. **12**: p. 223.
175. McInnes, B.T., et al., *Knowledge-based method for determining the meaning of ambiguous biomedical terms using information content measures of similarity.* AMIA Annu Symp Proc, 2011. **2011**: p. 895-904.
176. Cheng, W., J. Preiss, and M. Stevenson, *Scaling up WSD with automatically generated examples*, in *Proceedings of the 2012 Workshop on Biomedical Natural Language Processing*. 2012, Association for Computational Linguistics: Montreal, Canada. p. 231–239.
177. Yepes, A.J. and A.R. Aronson, *Knowledge-based and knowledge-lean methods combined in unsupervised word sense disambiguation*, in *Proceedings of the 2nd ACM SIGHIT International Health Informatics Symposium*. 2012, Association for Computing Machinery: Miami, Florida, USA. p. 733–736.
178. Stevenson, M., E. Agirre, and A. Soroa, *Exploiting domain information for Word Sense Disambiguation of medical documents.* J Am Med Inform Assoc, 2012. **19**(2): p. 235-40.
179. El-Rab, W.G., O.R. Zaïane, and M. El-Hajj, *Biomedical text disambiguation using UMLS*, in *Proceedings of the 2013 IEEE/ACM International Conference on Advances in Social Networks Analysis and Mining*. 2013, Association for Computing Machinery: Niagara, Ontario, Canada. p. 943–947.
180. Garla, V.N. and C. Brandt, *Knowledge-based biomedical word sense disambiguation: an evaluation and application to clinical document classification.* J Am Med Inform Assoc, 2013. **20**(5): p. 882-6.
181. McInnes, B.T. and T. Pedersen, *Evaluating measures of semantic similarity and relatedness to disambiguate terms in biomedical text.* J Biomed Inform, 2013. **46**(6): p. 1116-24.
182. McInnes, B.T. and M. Stevenson, *Determining the difficulty of Word Sense Disambiguation.* J Biomed Inform, 2014. **47**: p. 83-90.
183. Chasin, R., et al., *Word sense disambiguation in the clinical domain: a comparison of knowledge-rich and knowledge-poor unsupervised methods.* J Am Med Inform Assoc, 2014. **21**(5): p. 842-9.
184. Festag, S. and C. Spreckelsen, *Word Sense Disambiguation of Medical Terms via Recurrent Convolutional Neural Networks.* Stud Health Technol Inform, 2017. **236**: p. 8-15.
185. Jimeno Yepes, A., *Word embeddings and recurrent neural networks based on Long-Short Term Memory nodes in supervised biomedical word sense disambiguation.* J Biomed Inform, 2017. **73**: p. 137-147.
186. Duque, A., et al., *Co-occurrence graphs for word sense disambiguation in the biomedical domain.* Artif Intell Med, 2018. **87**: p. 9-19.

187. Pesaranghader, A., et al., *deepBioWSD: effective deep neural word sense disambiguation of biomedical text data.* J Am Med Inform Assoc, 2019. **26**(5): p. 438-446.
188. McCray, A.T., *Extending a natural language parser with UMLS knowledge.* Proc Annu Symp Comput Appl Med Care, 1991: p. 194-8.
189. Nelson, S.J., et al., *A semantic normal form for clinical drugs in the UMLS: early experiences with the VANDF.* Proc AMIA Symp, 2002: p. 557-61.
190. Nishimoto, N., et al., *Development of a medical-text parsing algorithm based on character adjacent probability distribution for Japanese radiology reports.* Methods Inf Med, 2008. **47**(6): p. 513-21.
191. Fan, J.W. and C. Friedman, *Deriving a probabilistic syntacto-semantic grammar for biomedicine based on domain-specific terminologies.* J Biomed Inform, 2011. **44**(5): p. 805-14.
192. Taboada, M., et al., *Combining open-source natural language processing tools to parse clinical practice guidelines.* Expert Sys: J. Knowl. Eng., 2013. **30**(1): p. 3–11.
193. Mitchell, K.J., et al., *Implementation and evaluation of a negation tagger in a pipeline-based system for information extract from pathology reports.* Stud Health Technol Inform, 2004. **107**(Pt 1): p. 663-7.
194. Dozier, C., et al., *Fast tagging of medical terms in legal text*, in *Proceedings of the 11th international conference on Artificial intelligence and law*. 2007, Association for Computing Machinery: Stanford, California. p. 253–260.
195. Liu, Y., et al., *Using SemRep to label semantic relations extracted from clinical text.* AMIA Annu Symp Proc, 2012. **2012**: p. 587-95.
196. Chen, A.T.M.P., R.M.M.L.S. Carriere, and S.J.M. Kaplan, *The User Knows What to Call It: Incorporating Patient Voice Through User-Contributed Tags on a Participatory Platform About Health Management.* Journal of Medical Internet Research, 2017. **19**(9).
197. Veytsman, B., *How to measure the consistency of the tagging of scientific papers?*, in *Proceedings of the 18th Joint Conference on Digital Libraries*. 2019, IEEE Press: Champaign, Illinois. p. 372–373.
198. Hersh, W.R., et al., *Empirical, automated vocabulary discovery using large text corpora and advanced natural language processing tools.* Proc AMIA Annu Fall Symp, 1996: p. 159-63.
199. McCray, A.T. and A.C. Browne, *Discovering the modifiers in a terminology data set.* Proc AMIA Symp, 1998: p. 780-4.
200. Kim, W. and W.J. Wilbur, *Corpus-based statistical screening for phrase identification.* J Am Med Inform Assoc, 2000. **7**(5): p. 499-511.
201. Travers, D.A. and S.W. Haas, *Evaluation of emergency medical text processor, a system for cleaning chief complaint text data.* Acad Emerg Med, 2004. **11**(11): p. 1170-6.
202. Wermter, J. and U. Hahn, *Effective grading of termhood in biomedical literature.* AMIA Annu Symp Proc, 2005: p. 809-13.
203. Ngomo, A.-C.N., *SIGNUM: a graph algorithm for terminology extraction*, in *Proceedings of the 9th international conference on Computational linguistics and intelligent text processing*. 2008, Springer-Verlag: Haifa, Israel. p. 85–95.
204. Luo, Z., R. Miotto, and C. Weng, *A human-computer collaborative approach to identifying common data elements in clinical trial eligibility criteria.* J Biomed Inform, 2013. **46**(1): p. 33-9.
205. Bentounsi, I. and Z. Boufaïda, *Disambiguation of semantic types in complex noun phrases for extracting candidate terms.* Int. J. Metadata Semant. Ontologies, 2015. **10**(2): p. 112–122.
206. Ruiz, M.E.R. and P. Srinivasan, *Combining machine learning and hierarchical structures for text categorization*. 2001, The University of Iowa.
207. Alecu, I., et al., *Mapping of the WHO-ART terminology on Snomed CT to improve grouping of related adverse drug reactions.* Stud Health Technol Inform, 2006. **124**: p. 833-8.

208. Aseervatham, S., E. Viennet, and Y. Bennani, *A Semantic Kernel for Semi-structured DocumentS*, in *Proceedings of the 2007 Seventh IEEE International Conference on Data Mining*. 2007, IEEE Computer Society. p. 403–408.
209. Aseervatham, S. and Y. Bennani, *Semi-structured document categorization with a semantic kernel.* Pattern Recogn., 2009. **42**(9): p. 2067–2076.
210. Yi, K. and J. Beheshti, *A hidden Markov model-based text classification of medical documents.* J. Inf. Sci., 2009. **35**(1): p. 67–81.
211. Luo, Z., S.B. Johnson, and C. Weng, *Semi-Automatically Inducing Semantic Classes of Clinical Research Eligibility Criteria Using UMLS and Hierarchical Clustering.* AMIA Annu Symp Proc, 2010. **2010**: p. 487-91.
212. Luo, Z., M. Yetisgen-Yildiz, and C. Weng, *Dynamic categorization of clinical research eligibility criteria by hierarchical clustering.* J Biomed Inform, 2011. **44**(6): p. 927-35.
213. Albitar, S., S. Fournier, and B. Espinasse, *Conceptualization Effects on MEDLINE Documents Classification Using Rocchio Method*, in *Proceedings of the The 2012 IEEE/WIC/ACM International Joint Conferences on Web Intelligence and Intelligent Agent Technology - Volume 01*. 2012, IEEE Computer Society. p. 462–466.
214. Albitar, S., S. Fournier, and B. Espinasse, *The impact of conceptualization on text classification*, in *Proceedings of the 13th international conference on Web Information Systems Engineering*. 2012, Springer-Verlag: Paphos, Cyprus. p. 326–339.
215. Garla, V.N. and C. Brandt, *Ontology-guided feature engineering for clinical text classification.* J Biomed Inform, 2012. **45**(5): p. 992-8.
216. McCray, A.T., et al., *UMLS knowledge for biomedical language processing.* Bull Med Libr Assoc, 1993. **81**(2): p. 184-94.
217. Klavans, J.L. and S. Muresan, *Evaluation of DEFINDER: a system to mine definitions from consumer-oriented medical text*, in *Proceedings of the 1st ACM/IEEE-CS joint conference on Digital libraries*. 2001, Association for Computing Machinery: Roanoke, Virginia, USA. p. 201–202.
218. McCray, A.T., et al., *Evaluating UMLS strings for natural language processing.* Proc AMIA Symp, 2001: p. 448-52.
219. Rindflesch, T.C. and M. Fiszman, *The interaction of domain knowledge and linguistic structure in natural language processing: interpreting hypernymic propositions in biomedical text.* J Biomed Inform, 2003. **36**(6): p. 462-77.
220. Bernhardt, P.J., S.M. Humphrey, and T.C. Rindflesch, *Determining prominent subdomains in medicine.* AMIA Annu Symp Proc, 2005: p. 46-50.
221. Wellner, B., J. Castaño, and J. Pustejovsky, *Adaptive string similarity metrics for biomedical reference resolution*, in *Proceedings of the ACL-ISMB Workshop on Linking Biological Literature, Ontologies and Databases: Mining Biological Semantics*. 2005, Association for Computational Linguistics: Detroit, Michigan. p. 9–16.
222. Torii, M. and H. Liu, *Headwords and suffixes in biomedical names*, in *Proceedings of the 2006 international conference on Knowledge Discovery in Life Science Literature*. 2006, Springer-Verlag: Singapore. p. 29–41.
223. Danger, R., et al., *A comparison of machine learning techniques for detection of drug target articles.* J Biomed Inform, 2010. **43**(6): p. 902-13.
224. Lingren, T., et al., *Pre-annotating Clinical Notes and Clinical Trial Announcements for Gold Standard Corpus Development: Evaluating the Impact on Annotation Speed and Potential Bias*, in *Proceedings of the 2012 IEEE Second International Conference on Healthcare Informatics, Imaging and Systems Biology*. 2012, IEEE Computer Society. p. 108.

225. Wu, S.T., et al., *Unified Medical Language System term occurrences in clinical notes: a large-scale corpus analysis.* J Am Med Inform Assoc, 2012. **19**(e1): p. e149-56.
226. Rudniy, A., M. Song, and J. Geller, *Mapping biological entities using the longest approximately common prefix method.* BMC Bioinformatics, 2014. **15**: p. 187.
227. Pandey, C., et al., *Improving RNN with Attention and Embedding for Adverse Drug Reactions*, in *Proceedings of the 2017 International Conference on Digital Health*. 2017, Association for Computing Machinery: London, United Kingdom. p. 67–71.
228. Chen, Z., et al., *Evaluating semantic relations in neural word embeddings with biomedical and general domain knowledge bases.* BMC Med Inform Decis Mak, 2018. **18**(Suppl 2): p. 65.
229. Wang, Y., et al., *A comparison of word embeddings for the biomedical natural language processing.* J Biomed Inform, 2018. **87**: p. 12-20.
230. Zolnoori, M., et al., *A systematic approach for developing a corpus of patient reported adverse drug events: A case study for SSRI and SNRI medications.* J Biomed Inform, 2019. **90**: p. 103091.
