# Supplementary material for: The Unified Medical Language System at 30 Years and How It Is Used and Published: Systematic Review and Content Analysis
Source: JMIR Med Inform. 2021 Aug 27;9(8):e20675. doi: 10.2196/20675 (PMC8433943; doi:10.2196/20675)
Supplement: Multimedia Appendix 9 [file medinform_v9i8e20675_app9.pdf]

Multimedia Appendix 9. Unified Medical Language System publications in ontology and modeling.

Author	Publication year	Title	What was UMLS used for?
**Classification/Taxonomy**			
Chute, et al[1]	1996	The content coverage of clinical classifications. For The Computer-Based Patient Record Institute's Work Group on Codes & Structures	UMLS coverage, content coverage of clinical classifications
Pratt[2]	1997	Dynamic organization of search results using the UMLS	Clustering technique, classification technique, grouping
Campbell, et al[3]	1999	A technique for semantic classification of unknown words using UMLS resources	Semantic classification, unknown word, UMLS
Bodenreider[4]	2000	Using UMLS semantics for classification purposes	UMLS, semantic classification, automatic classification, mapping
Burgun, et al[5]	2001	Aspects of the taxonomic relation in the biomedical domain	Taxonomy of living organisms, classification of diseases, UMLS
Niu, et al[6]	2005	Analysis of polarity information in medical text	Polarity information analysis, classification, UMLS
Hou, et al[7]	2006	Classifying biological full-text articles for multi-database curation	Article classification, UMLS as a domain-specific resource
Kobayashi, et al[8]	2006	Representing clinical questions by semantic type for better classification	Classification, generic taxonomic categories, UMLS
McCowan, et al[9]	2006	Classification of cancer stage from free-text histology reports	Classification of cancer stage, histology reports, UMLS concept identification
Fan, et al[10]	2007	Combining contextual and lexical features to classify UMLS concepts	UMLS classifier, contextual features, lexical features
Fan, et al[11]	2007	Using contextual and lexical features to restructure and validate the classification of biomedical concepts	Concept classification, contextual features, lexical features, validation
Fan, et al[12]	2007	Using distributional analysis to semantically classify UMLS concepts	NLP, UMLS, semantic classification
Wen, et al[13]	2007	Semantic Smoothing the Multinomial Naive Bayes for Biomedical Literature Classification	Biomedical documents indexing by UMLS, text classification
Fan, et al[14]	2008	Semantic reclassification of the UMLS concepts	Semantic classification of UMLS concepts, text mining
Liu, et al[15]	2009	Integrative disease classification based on cross-platform microarray data	Disease classification, cross-platform microarray data, text annotation
Riedl, et al[16]	2010	Using the UMLS and Simple Statistical Methods to Semantically	Categorization of death causes, NLP, UMLS

		Categorize Causes of Death on Death Certificates	
Lakiotaki, et al[17]	2013	Towards personalized medical document classification by leveraging UMLS semantic network	Automated information classification, medical document indexing
Rahman, et al[18]	2013	Comparative study of classification techniques on biomedical data from hypertext documents	Mining data from web contents, classification techniques, biomedical entities from UMLS
Luo, et al[19]	2014	Automatic lymphoma classification with sentence subgraph mining from pathology reports	Automatic classification, lymphoma classification, pathology report, relation identification
Morid, et al[20]	2016	Classification of clinically useful sentences in clinical evidence resources	Classification of sentences, extraction of UMLS concept
Weng, et al[21]	2017	Medical subdomain classification of clinical notes using a machine learning-based natural language processing approach	UMLS for feature extraction from clinical notes, medical subdomain classifiers
**Modeling**			
Altmann, et al[22]	1999	A model for integration and continuous development of standards for tumour documentation using relational database techniques and extensible markup language	Tumor documentation model, integration of various sources, common dictionary, UMLS
Aymard, et al[23]	2003	Modeling and implementing a health information provider on the Internet	Modeling, health information provider, UMLS
Meng, et al[24]	2005	Generating models of surgical procedures using UMLS concepts and multiple sequence alignment	Modeling, surgical procedure, UMLS
Sadeghi, et al[25]	2005	Ontology Driven Construction of a Knowledgebase for Bayesian Decision Models Based on UMLS	Bayesian decision model, ontology, knowledge base
Lee, et al[26]	2006	The Tissue Microarray Object Model: A Data Model for Storage, Analysis, and Exchange of Tissue Microarray Experimental Data	The clinical and histopathologic information model, tissue microarray experimental data
Kamal, et al[27]	2007	Development of an ontology-anchored data warehouse meta-model	Meta-model, abstration layer, UMLS, ontology
Chen, et al[28]	2008	Comparing and consolidating two heuristic metaschema	Metaschema, UMLS's semantic network, consolidation
Maisonnasse, et al[29]	2008	Multiplying Concept Sources for Graph Modeling	Language modeling, graph modeling, UMLS as domain knowledge

Chen, et al[30]	2009	Modeling multi-typed structurally viewed chemicals with the UMLS Refined Semantic Network	Modeling, UMLS, disambiguation chemicals
Taboada, et al[31]	2009	From natural language descriptions in clinical guidelines to relationships in an ontology	Clinical practice guideline modeling, entity recognition, relation extraction
Kaiser, et al[32]	2010	Supporting the abstraction of clinical practice guidelines using information extraction	Modeling clinical practice guidelines, information extraction, UMLS Semantic Network
Painter[33]	2010	Toward automating an inference model on unstructured terminologies: OXMIS case study	Inference model. Automatic classification, automatic categorization, UMLS concepts
Min, et al[34]	2012	Clinical data analysis using ontology-guided rule learning	Modeling, UMLS as a domain knowledge source
Minard, et al[35]	2013	Supporting Computer-interpretable Guidelines' Modeling by Automatically Classifying Clinical Actions	Modeling computer-interpretable clinical practice guidelines, Semantic Types of the UMLS
Saripalle[36]	2015	Need for a Specialized Metamodel for Biomedical and Health Informatics Domain	Minimal metamodel, MOF and UMLS Semantic Network
Fan, et al[37]	2017	Semantic Modeling for Exposomics with Exploratory Evaluation in Clinical Context	Exposome modeling, UMLS for identifying exposome concepts, discharge summaries
Zhao, et al[38]	2018	Using data-driven sublanguage pattern mining to induce knowledge models: application in medical image reports knowledge representation.	pattern mining, tagging by UMLS, image report, knowledge representation
Riepenhausen, et al[39]	2019	Portal of Medical Data Models: Status 2018	Medical data model, UMLS for annotation
**Ontology**			
Li, et al[40]	2000	Ontology acquisition from on-line knowledge sources	Ontology acquisition, online knowledge sources, UMLS as a resource
Hahn, et al[41]	2002	Massive bio-ontology engineering for NLP	Ontology, description logic system, knowledge extraction
McCray, et al[42]	2002	The lexical properties of the gene ontology	UMLS, GO, properties
Hahn, et al[43]	2003	Towards a broad-coverage biomedical ontology based on description logics	UMLS, automatic concept definition generation, integrity check
Hahn, et al[44]	2004	Mereological semantics for bio-ontologies	UMLS, GO, taxonomic and a mereological order
Deitzer, et al[45]	2006	Coverage of clinical trials tasks in existing ontologies	Coverage of clinical trial tasks, UMLS, SNOMED CT, NCI thesaurus

Lee, et al[46]	2006	Semantic enrichment for medical ontologies	Semantic enrichment, two-level ontology, UMLS
Marquet, et al[47]	2006	Aligning biomedical ontologies using lexical methods and the UMLS: the case of disease ontologies	Ontology alignments, mapping, UMLS
Srinivasan, et al[48]	2006	Semantic web representation of LOINC: an ontological perspective	UMLS, semantic web representation of LOINC
Vizenor, et al[49]	2006	Enhancing biomedical ontologies through alignment of semantic relationships: exploratory approaches	Ontology, semantic alignment improvement, alignment of relationships
Marquet, et al[50]	2007	A method exploiting syntactic patterns and the UMLS semantics for aligning biomedical ontologies: the case of OBO disease ontologies	Biological ontology alignment, syntactic patterns, UMLS
Ghazvinian, et al[51]	2009	What Four Million Mappings Can Tell You about Two Hundred Ontologies	BioPortal, UMLS, ontologies, mapping
Jean-Mary, et al[52]	2009	Ontology matching with semantic verification	Automated Semantic Matching of Ontologies, lexical and structural characteristics
Osborne, et al[53]	2009	Annotating the human genome with Disease Ontology	Gene disease relationship discovery, disease annotation for the human genome
Jimeneze-Ruiz, et al[54]	2010	Towards a UMLS-based silver standard for matching biomedical ontologies	Alignment of NCI, FMA and SNOMED CT
Ernesto, et al[55]	2011	Towards more challenging problems for ontology matching tools	Evaluation of ontology matching tools, mapping sets, UMLS
Silachan, et al[56]	2011	Domain Ontology Health Informatics Service from Text Medical Data Classification	Domain Ontology, UMLS, vocabulary based system
Slabbekoorn, et al[57]	2012	Domain-Aware ontology matching	The inherent heterogeneity of datasets, evaluation against UMLS
Flahive, et al[58]	2013	Ontology as a Service (OaaS): a case for sub-ontology merging on the cloud	Cloud computing, UMLS meta-thesaurus ontology
Flahive, et al[59]	2013	Ontology as a Service (OaaS): extracting and replacing sub-ontologies on the cloud	UMLS meta-thesaurus ontology, Cloud computing
Pesquita, et al[60]	2013	To repair or not to repair: reconciling correctness and coherence in ontology reference alignments	Ontology matching, the alignment repair process, UMLS metathesaurus
Flahive, et al[61]	2014	Merging sub-ontologies	UMLS, WordNet, merging, subontologies
Hajagos, et al[62]	2014	Linking Clinicians to Biomedical Researchers: An Application of the	Clinical experience represented by ISF ontology, ICD9CM, MeSH, UMLS

		ISF Ontology at Stony Brook Medicine	
Uchibayashi, et al[63]	2014	A domain specific sub-ontology derivation end-user tool for the Semantic Grid	Sub-ontology, evaluation using the UMLS Semantic Network
Cui[64]	2015	COHeRE: Cross-Ontology Hierarchical Relation Examination for Ontology Quality Assurance	Ontology quality assurance, inconsistency detection, error detection
Saripalle[65]	2015	UMLS Semantic Network as a UML Metamodel for Improving Biomedical Ontology and Application Modeling	Meta-modeling, ontology, UMLS Semantic Network
Hempelmann, et al[66]	2016	An entropy-based evaluation method for knowledge bases of medical information systems	Ontology evaluation, ontological-semantic knowledge base
Iqtidar, et al[67]	2017	A biomedical ontology on genetic disease	Knowledge mining between disease and gene, UMLS, ontology,
Wei, et al[68]	2019	Construction of Disease Similarity Networks Using Concept Embedding and Ontology	Mental disease similarity network; concept embedding, ontology
**Representation**			
Campbell, et al[69]	1992	Representation of clinical data using SNOMED III and conceptual graphs	SNOMED III, conceptual graphs, data representation, UMLS
Kahn[70]	1999	Standard Generalized Markup Language for self-defining structured reports	Markup language, self-defining structured reports
Michael, et al[71]	2001	The role of definitions in biomedical concept representation	Foundational Model of Anatomy, UMLS, concept representation
Burgun, et al[72]	2002	Representation of roles in biomedical ontologies: a case study in functional genomics	Functional genomics ontology, UMLS, GO, iron metabolism, mapping
Lee, et al[73]	2004	Toward cooperative genomic knowledge inference	Distributed genomic knowledge reasoning, network complying with the UMLS
Bertaud, et al[74]	2005	Toward a unified representation of findings in clinical radiology	Unified representation, clinical radiology, UMLS, DCMR
Bertaud, et al[75]	2008	A unified representation of findings in clinical radiology using the UMLS and DICOM	Radiology representation, UMLS, DICOM, generic template
Denecke[76]	2008	Enhancing knowledge representations by ontological relations	Knowledge representation
Denecke[77]	2008	Semantic structuring of and information extraction from medical documents using the UMLS	Automatic generation of knowledge representation, UMLS, SeReMeD

Patel, et al[78]	2008	ECRL: an eligibility criteria representation language based on the UMLS Semantic Network	Computable representation, eligibility criteria representation language
Friedlin, et al[79]	2011	An evaluation of the UMLS in representing corpus derived clinical concepts	UMLS representation evaluation, chest x-ray reports, discharge summary
Becker, et al[80]	2017	Semi-Automatic Mark-Up and UMLS Annotation of Clinical Guidelines	Clinical pathway, UMLS for mark up and annotation for clinical guidelines

References:

1. Chute, C.G., et al., *The content coverage of clinical classifications. For The Computer-Based Patient Record Institute's Work Group on Codes & Structures.* J Am Med Inform Assoc, 1996. **3**(3): p. 224-33.
2. Pratt, W., *Dynamic organization of search results using the UMLS.* Proc AMIA Annu Fall Symp, 1997: p. 480-4.
3. Campbell, D.A. and S.B. Johnson, *A technique for semantic classification of unknown words using UMLS resources.* Proc AMIA Symp, 1999: p. 716-20.
4. Bodenreider, O., *Using UMLS semantics for classification purposes.* Proc AMIA Symp, 2000: p. 86-90.
5. Burgun, A. and O. Bodenreider, *Aspects of the taxonomic relation in the biomedical domain*, in *Proceedings of the international conference on Formal Ontology in Information Systems - Volume 2001*. 2001, Association for Computing Machinery: Ogunquit, Maine, USA. p. 222–233.
6. Niu, Y., et al., *Analysis of polarity information in medical text.* AMIA Annu Symp Proc, 2005: p. 570-4.
7. Hou, W.-J., C. Lee, and H.-H. Chen, *Classifying biological full-text articles for multi-database curation*, in *Proceedings of the Eleventh Conference of the European Chapter of the Association for Computational Linguistics: Posters & Demonstrations*. 2006, Association for Computational Linguistics: Trento, Italy. p. 159–162.
8. Kobayashi, T. and C.R. Shyu, *Representing clinical questions by semantic type for better classification.* AMIA Annu Symp Proc, 2006: p. 987.
9. McCowan, I., D. Moore, and M.J. Fry, *Classification of cancer stage from free-text histology reports.* Conf Proc IEEE Eng Med Biol Soc, 2006. **2006**: p. 5153-6.
10. Fan, J.W. and C. Friedman, *Combining contextual and lexical features to classify UMLS concepts.* AMIA Annu Symp Proc, 2007: p. 231-5.
11. Fan, J.W., H. Xu, and C. Friedman, *Using contextual and lexical features to restructure and validate the classification of biomedical concepts.* BMC Bioinformatics, 2007. **8**: p. 264.
12. Fan, J.W., H. Xu, and C. Friedman, *Using distributional analysis to semantically classify UMLS concepts.* Stud Health Technol Inform, 2007. **129**(Pt 1): p. 519-23.
13. Wen, J. and Z. Li, *Semantic Smoothing the Multinomial Naive Bayes for Biomedical Literature Classification*, in *Proceedings of the 2007 IEEE International Conference on Granular Computing*. 2007, IEEE Computer Society. p. 648.
14. Fan, J.W. and C. Friedman, *Semantic reclassification of the UMLS concepts.* Bioinformatics, 2008. **24**(17): p. 1971-3.
15. Liu, C.C., et al., *Integrative disease classification based on cross-platform microarray data.* BMC Bioinformatics, 2009. **10 Suppl 1**: p. S25.

16. Riedl, B., N. Than, and M. Hogarth, *Using the UMLS and Simple Statistical Methods to Semantically Categorize Causes of Death on Death Certificates.* AMIA Annu Symp Proc, 2010. **2010**: p. 677-81.
17. Lakiotaki, K., et al., *Towards personalized medical document classification by leveraging UMLS semantic network*, in *Proceedings of the second international conference on Health Information Science*. 2013, Springer-Verlag: London, UK. p. 93–104.
18. Rahman, R.M. and S. Salahuddin, *Comparative study of classification techniques on biomedical data from hypertext documents.* Int. J. Knowl. Eng. Soft Data Paradigm., 2013. **4**(1): p. 21–41.
19. Luo, Y., et al., *Automatic lymphoma classification with sentence subgraph mining from pathology reports.* J Am Med Inform Assoc, 2014. **21**(5): p. 824-32.
20. Morid, M.A., et al., *Classification of clinically useful sentences in clinical evidence resources.* J Biomed Inform, 2016. **60**: p. 14-22.
21. Weng, W.H., et al., *Medical subdomain classification of clinical notes using a machine learning-based natural language processing approach.* BMC Med Inform Decis Mak, 2017. **17**(1): p. 155.
22. Altmann, U., et al., *A model for integration and continuous development of standards for tumour documentation using relational database techniques and extensible markup language.* Stud Health Technol Inform, 1999. **68**: p. 895-8.
23. Aymard, S., et al., *Modeling and implementing a health information provider on the Internet.* Stud Health Technol Inform, 2003. **95**: p. 89-94.
24. Meng, F., et al., *Generating models of surgical procedures using UMLS concepts and multiple sequence alignment.* AMIA Annu Symp Proc, 2005: p. 520-4.
25. Sadeghi, S., A. Barzi, and J.W. Smith, *Ontology Driven Construction of a Knowledgebase for Bayesian Decision Models Based on UMLS.* Stud Health Technol Inform, 2005. **116**: p. 223-8.
26. Lee, H.W., et al., *The Tissue Microarray Object Model: A Data Model for Storage, Analysis, and Exchange of Tissue Microarray Experimental Data.* Archives of Pathology & Laboratory Medicine, 2006. **130**(7): p. 1004-13.
27. Kamal, J., T. Borlawsky, and P.R. Payne, *Development of an ontology-anchored data warehouse meta-model.* AMIA Annu Symp Proc, 2007: p. 1001.
28. Chen, Y., et al., *Comparing and consolidating two heuristic metaschemas.* J Biomed Inform, 2008. **41**(2): p. 293-317.
29. Maisonnasse, L., E. Gaussier, and J.P. Chevallet, *Multiplying Concept Sources for Graph Modeling*, in *Advances in Multilingual and Multimodal Information Retrieval: 8th Workshop of the Cross-Language Evaluation Forum, CLEF 2007, Budapest, Hungary, September 19-21, 2007, Revised Selected Papers*. 2008, Springer-Verlag. p. 585–592.
30. Chen, L., et al., *Modeling multi-typed structurally viewed chemicals with the UMLS Refined Semantic Network.* J Am Med Inform Assoc, 2009. **16**(1): p. 116-31.
31. Taboada, M., et al., *From natural language descriptions in clinical guidelines to relationships in an ontology*, in *Proceedings of the 2009 AIME international conference on Knowledge Representation for Health-Care: data, Processes and Guidelines*. 2009, Springer-Verlag: Verona, Italy. p. 26–37.
32. Kaiser, K. and S. Miksch, *Supporting the abstraction of clinical practice guidelines using information extraction*, in *Proceedings of the Natural language processing and information systems, and 15th international conference on Applications of natural language to information systems*. 2010, Springer-Verlag: Cardiff, UK. p. 304–311.
33. Painter, J.L., *Toward automating an inference model on unstructured terminologies: OXMIS case study.* Adv Exp Med Biol, 2010. **680**: p. 645-51.

34. Min, H. and J. Wojtusiak, *Clinical data analysis using ontology-guided rule learning*, in *Proceedings of the 2nd international workshop on Managing interoperability and compleXity in health systems*. 2012, Association for Computing Machinery: Maui, Hawaii, USA. p. 17–22.
35. Minard, A.-L. and K. Kaiser, *Supporting Computer-interpretable Guidelines' Modeling by Automatically Classifying Clinical Actions*, in *Revised Selected Papers of the AIME 2013 Joint Workshop on Process Support and Knowledge Representation in Health Care - Volume 8268*. 2013, Springer-Verlag. p. 39–52.
36. Saripalle, R.K., *Need for a Specialized Metamodel for Biomedical and Health Informatics Domain*, in *Revised Selected Papers of the International Conference on Smart Health - Volume 9545*. 2015, Springer-Verlag: Phoenix, AZ, USA. p. 99–104.
37. Fan, J.W., J. Li, and Y.A. Lussier, *Semantic Modeling for Exposomics with Exploratory Evaluation in Clinical Context.* J Healthc Eng, 2017. **2017**: p. 3818302.
38. Zhao, Y., et al., *Using data-driven sublanguage pattern mining to induce knowledge models: application in medical image reports knowledge representation.* BMC Med Inform Decis Mak, 2018. **18**(1): p. 61.
39. Riepenhausen, S., et al., *Portal of Medical Data Models: Status 2018.* Stud Health Technol Inform, 2019. **258**: p. 239-240.
40. Li, Q., et al., *Ontology acquisition from on-line knowledge sources.* Proc AMIA Symp, 2000: p. 497-501.
41. Hahn, U. and S. Schulz, *Massive bio-ontology engineering for NLP*, in *Proceedings of the second international conference on Human Language Technology Research*. 2002, Morgan Kaufmann Publishers Inc.: San Diego, California. p. 68–75.
42. McCray, A.T., A.C. Browne, and O. Bodenreider, *The lexical properties of the gene ontology.* Proc AMIA Symp, 2002: p. 504-8.
43. Hahn, U. and S. Schulz, *Towards a broad-coverage biomedical ontology based on description logics.* Pac Symp Biocomput, 2003: p. 577-88.
44. Hahn, U., S. Schulz, and K. Markó, *Mereological semantics for bio-ontologies*, in *Proceedings of the 19th national conference on Artifical intelligence*. 2004, AAAI Press: San Jose, California. p. 257–262.
45. Deitzer, J.R., P.R. Payne, and J.B. Starren, *Coverage of clinical trials tasks in existing ontologies.* AMIA Annu Symp Proc, 2006: p. 903.
46. Lee, Y. and J. Geller, *Semantic enrichment for medical ontologies.* J Biomed Inform, 2006. **39**(2): p. 209-26.
47. Marquet, G., J. Mosser, and A. Burgun, *Aligning biomedical ontologies using lexical methods and the UMLS: the case of disease ontologies.* Stud Health Technol Inform, 2006. **124**: p. 781-6.
48. Srinivasan, A., et al., *Semantic web representation of LOINC: an ontological perspective.* AMIA Annu Symp Proc, 2006: p. 1107.
49. Vizenor, L., et al., *Enhancing biomedical ontologies through alignment of semantic relationships: exploratory approaches.* AMIA Annu Symp Proc, 2006: p. 804-8.
50. Marquet, G., J. Mosser, and A. Burgun, *A method exploiting syntactic patterns and the UMLS semantics for aligning biomedical ontologies: the case of OBO disease ontologies.* Int J Med Inform, 2007. **76 Suppl 3**: p. S353-61.
51. Ghazvinian, A., et al., *What Four Million Mappings Can Tell You about Two Hundred Ontologies*, in *Proceedings of the 8th International Semantic Web Conference*. 2009, Springer-Verlag: Chantilly, VA. p. 229–242.
52. Jean-Mary, Y.R., E.P. Shironoshita, and M.R. Kabuka, *Ontology matching with semantic verification.* Web Semant., 2009. **7**(3): p. 235–251.

53. Osborne, J.D., et al., *Annotating the human genome with Disease Ontology.* BMC Genomics, 2009. **10 Suppl 1**: p. S6.
54. Jiménez-Ruiz, E., et al., *Towards a UMLS-based silver standard for matching biomedical ontologies*, in *Proceedings of the 5th International Conference on Ontology Matching - Volume 689*. 2010, CEUR-WS.org: Shanghai, China. p. 220–221.
55. Jiménez-Ruiz, E. and B.C. Grau, *Towards more challenging problems for ontology matching tools*, in *Proceedings of the 6th International Conference on Ontology Matching - Volume 814*. 2011, CEUR-WS.org: Bonn, Germany. p. 236–237.
56. Silachan, K. and P. Tantatsanawong, *Domain Ontology Health Informatics Service from Text Medical Data Classification*, in *Proceedings of the 2011 Annual SRII Global Conference*. 2011, IEEE Computer Society. p. 357–362.
57. Slabbekoorn, K., L. Hollink, and G.-J. Houben, *Domain-Aware ontology matching*, in *Proceedings of the 11th international conference on The Semantic Web - Volume Part I*. 2012, Springer-Verlag: Boston, MA. p. 542–558.
58. Flahive, A., D. Taniar, and W. Rahayu, *Ontology as a Service (OaaS): a case for sub-ontology merging on the cloud.* J. Supercomput., 2013. **65**(1): p. 185–216.
59. Flahive, A., D. Taniar, and W. Rahayu, *Ontology as a Service (OaaS): extracting and replacing sub-ontologies on the cloud.* Cluster Computing, 2013. **16**(4): p. 947–960.
60. Pesquita, C., et al., *To repair or not to repair: reconciling correctness and coherence in ontology reference alignments*, in *Proceedings of the 8th International Conference on Ontology Matching - Volume 1111*. 2013, CEUR-WS.org: Sydney, Australia. p. 13–24.
61. Flahive, A., D. Taniar, and W. Rahayu, *Merging sub-ontologies.* Int. J. Web Grid Serv., 2014. **10**(2/3): p. 273–295.
62. Hajagos, J.G. and V. Agwan, *Linking Clinicians to Biomedical Researchers: An Application of the ISF Ontology at Stony Brook Medicine*, in *Proceedings of the 2014 IEEE 27th International Symposium on Computer-Based Medical Systems*. 2014, IEEE Computer Society. p. 503–504.
63. Uchibayashi, T., B.O. Apduhan, and N. Shiratori, *A domain specific sub-ontology derivation end-user tool for the Semantic Grid.* Telecommun. Syst., 2014. **55**(1): p. 125–135.
64. Cui, L., *COHeRE: Cross-Ontology Hierarchical Relation Examination for Ontology Quality Assurance.* AMIA Annu Symp Proc, 2015. **2015**: p. 456-65.
65. Saripalle, R.K., *UMLS Semantic Network as a UML Metamodel for Improving Biomedical Ontology and Application Modeling.* Int. J. Healthc. Inf. Syst. Inform., 2015. **10**(2): p. 34–56.
66. Hempelmann, C.F., et al., *An entropy-based evaluation method for knowledge bases of medical information systems.* Expert Syst. Appl., 2016. **46**(C): p. 262–273.
67. Iqtidar, A., et al., *A biomedical ontology on genetic disease*, in *Proceedings of the Second International Conference on Internet of things, Data and Cloud Computing*. 2017, Association for Computing Machinery: Cambridge, United Kingdom. p. Article 67.
68. Wei, D.H., et al., *Construction of Disease Similarity Networks Using Concept Embedding and Ontology.* Stud Health Technol Inform, 2019. **264**: p. 442-446.
69. Campbell, K.E. and M.A. Musen, *Representation of clinical data using SNOMED III and conceptual graphs.* Proc Annu Symp Comput Appl Med Care, 1992: p. 354-8.
70. Kahn, C.E., Jr., *Standard Generalized Markup Language for self-defining structured reports.* Int J Med Inform, 1999. **53**(2-3): p. 203-11.
71. Michael, J., J.L. Mejino, Jr., and C. Rosse, *The role of definitions in biomedical concept representation.* Proc AMIA Symp, 2001: p. 463-7.
72. Burgun, A., et al., *Representation of roles in biomedical ontologies: a case study in functional genomics.* Proc AMIA Symp, 2002: p. 86-90.

73. Lee, C.-W. and C.-H. Huang, *Toward cooperative genomic knowledge inference.* Parallel Comput., 2004. **30**(9–10): p. 1127–1135.
74. Bertaud, V., et al., *Toward a unified representation of findings in clinical radiology.* Stud Health Technol Inform, 2005. **116**: p. 671-6.
75. Bertaud, V., et al., *A unified representation of findings in clinical radiology using the UMLS and DICOM.* Int J Med Inform, 2008. **77**(9): p. 621-9.
76. Denecke, K., *Enhancing knowledge representations by ontological relations.* Stud Health Technol Inform, 2008. **136**: p. 791-6.
77. Denecke, K., *Semantic structuring of and information extraction from medical documents using the UMLS.* Methods Inf Med, 2008. **47**(5): p. 425-34.
78. Patel, C.O. and C. Weng, *ECRL: an eligibility criteria representation language based on the UMLS Semantic Network.* AMIA Annu Symp Proc, 2008: p. 1084.
79. Friedlin, J. and M. Overhage, *An evaluation of the UMLS in representing corpus derived clinical concepts.* AMIA Annu Symp Proc, 2011. **2011**: p. 435-44.
80. Becker, M. and B. Bockmann, *Semi-Automatic Mark-Up and UMLS Annotation of Clinical Guidelines.* Stud Health Technol Inform, 2017. **245**: p. 294-297.
