# Supplementary material for: The Unified Medical Language System at 30 Years and How It Is Used and Published: Systematic Review and Content Analysis
Source: JMIR Med Inform. 2021 Aug 27;9(8):e20675. doi: 10.2196/20675 (PMC8433943; doi:10.2196/20675)
Supplement: Multimedia Appendix 10 [file medinform_v9i8e20675_app10.pdf]

Multimedia Appendix 10. Publications that used the Unified Medical Language System in patient care.

**Author**	**Publication year**	**Title**	**What was UMLS used for?**
Miller, et al[1]	1992	CHARTLINE: providing bibliographic references relevant to patient charts using the UMLS Metathesaurus Knowledge Sources	CHARTLINE, provide bibliographic references, UMLS
Schuyler, et al[2]	1992	SIG/MED and SIG/ALP—decision-support systems in the Unified Medical Language System (UMLS)	UMLS, decision support system
Cimino[3]	1995	Use of the Unified Medical Language System in patient care at the Columbia-Presbyterian Medical Center	UMLS, patient care
Johnson, et al[4]	1997	The rubber meets the road: integrating the Unified Medical Language System Knowledge Source Server into the computer-based patient record	Integration of UMLS with patient's records, integration of codification into clinical workflow
Geissbuhler, et al[5]	1998	Clinical application of the UMLS in a computerized order entry and decision-support system	UMLS application, CPOE, CDSS
Van Mulligen[6]	1998	UMLS-based access to CPR data	UMLS, mapping CPR terms to UMLS concepts
Van Mulligen[7]	1999	UMLS-based access to CPR data. Unified Medical Language Systems	UMLS-based access to CPR, mapping CPR terms to UMLS concepts
Boxwala, et al[8]	2003	Coverage of patient safety terms in the UMLS metathesaurus	UMLS coverage, patient safety
Coonan, et al[9]	2004	Medical Informatics Standards Applicable to Emergency Department Information Systems: Making Sense of the Jumble	Medical informatics standards, UMLS as an example, emergency department
Currie, et al[10]	2004	Development and representation of a fall-injury risk assessment instrument in a clinical information system	Fall risk assessment instrument, inpatient safety, injury prevention
Kawamoto, et al[11]	2006	Design, implementation, use, and preliminary evaluation of an UMLS-enabled terminology Web service for clinical decision support	UMLS for clinical decision support, inter-vocabulary translation, terminology services
Tolentino, et al[12]	2006	Concept negation in free text components of vaccine safety reports	Negation identification, adverse events, vaccine safety

Tolentino, et al[13]	2007	A UMLS-based spell checker for natural language processing in vaccine safety	Vaccine safety, vaccine adverse events, UMLS concepts extraction
Lee, et al[14]	2008	Effectiveness of automatic acute stroke alert system based on UMLS mapped local terminology codes at emergency department	Automatic acute stroke alert system, emergency department
Pires, et al[15]	2008	A UMLS interoperable solution to support collaborative diagnosis decision making over the internet	UMLS interoperable solution, clinical decision support
Hashmi, et al[16]	2009	Computerization framework for clinical practice guidelines by extending the XML guidelines element model (GEM)	Clinical practice guidelines, computerization framework
Bodenreider, et al[17]	2010	Investigating drug classes in biomedical terminologies from the perspective of clinical decision support	Drug classes, UMLS, CDS, drug-drug interaction
Fujita, et al[18]	2010	Virtual doctor system (VDS): medical decision reasoning based on physical and mental ontologies	UMLS for testing, emotional modeling and physical views, mental ontology
Bonacin, et al[19]	2011	Careflow personalization services: concepts and tool for the evaluation of computer-interpretable guidelines	Computer-interpretable Clinical Guidelines, personalized treatment plans,
McCoy, et al[20]	2011	A prototype knowledge base and SMART app to facilitate organization of patient medications by clinical problems	Problem-medication links, medication organization
Lehman, et al[21]	2012	Risk stratification of ICU patients using topic models inferred from unstructured progress notes	Hospital mortality prediction, ICU risk stratification, UMLS
Sanchez-Garzon, et al [22]	2012	An approach for representing and managing medical exceptions in care pathways based on temporal hierarchical planning techniques	Personalized care pathways, rule represented by UMLS
Winnenburg, et al[23]	2012	Issues in creating and maintaining value sets for clinical quality measures	Clinical quality measures, value sets, UMLS
Patil, et al[24]	2013	Estimating personalized risk ranking using laboratory test and medical knowledge (UMLS)	Personalized risk ranking, lab test, UMLS
Valkenhoef, et al[25]	2013	ADDIS: A decision support system for evidence-based medicine	Safety evaluation of medical treatments, decision support system, UMLS

MacKellar, et al[26]	2014	Patient-Centered Clinical Trials Decision Support using Linked Open Data	Clinical trials query, UMLS, patient's decision support
Kuang, et al[27]	2015	Representation of Functional Status Concepts from Clinical Documents and Social Media Sources by Standard Terminologies	UMLS coverage of functional status, outcome measures
Shivade, et al[28]	2015	Comparison of UMLS terminologies to identify risk of heart disease using clinical notes	Automatic identification of risk factors for heart diseases
Varghese, et al[29]	2015	Standardized Cardiovascular Quality Assurance Forms with Multilingual Support, UMLS Coding and Medical Concept Analyses	Standardized quality assurance, cardiovascular procedures, UMLS concepts
Varghese, et al[30]	2015	Standardized quality assurance forms for organ transplantations with multilingual support, open access and UMLS coding	Clinical quality assurance, organ transplantation, UMLS for form elements
Choong, et al[31]	2017	Linking clinical quality indicators to research evidence - a case study in asthma management for children	Clinical quality indicator, outcome measures, asthma management
Moreira, et al[32]	2017	Enhancing Collaborative Case Diagnoses Through Unified Medical Language System-Based Disambiguation: A Case Study of the Zika Virus	Collaborative Case Diagnoses, Zika virus, UMLS, disambiguation
Bakal, et al[33]	2018	Exploiting semantic patterns over biomedical knowledge graphs for predicting treatment and causative relations	NLP, causal relations identification, supervised predictive models
Le, et al[34]	2018	Risk prediction using natural language processing of electronic mental health records in an inpatient forensic psychiatry setting	Risk of harm prediction, psychiatry, UMLS
Modaresnezhad, et al[35]	2019	A rule-based semantic approach for data integration, standardization and dimensionality reduction utilizing the UMLS: Application to predicting bariatric surgery outcomes	Predicting bariatric surgery outcomes, rule-based semantic networks, dimensionality reduction

References

1. Miller, R.A., et al., *CHARTLINE: providing bibliographic references relevant to patient charts using the UMLS Metathesaurus Knowledge Sources.* Proc Annu Symp Comput Appl Med Care, 1992: p. 86-90.
2. Schuyler, P., *SIG/MED and SIG/ALP—decision-support systems in the Unified Medical Language System (UMLS)*, in *Proceedings of the 55th annual meeting on Celebrating change : information

management on the move: information management on the move. 1992, American Society for Information Science: Pittsburgh, Pennsylvania, USA. p. 347.
3. Cimino, J.J., *Use of the Unified Medical Language System in patient care at the Columbia-Presbyterian Medical Center.* Methods Inf Med, 1995. **34**(1-2): p. 158-64.
4. Johnson, K.B. and E.B. George, *The rubber meets the road: integrating the Unified Medical Language System Knowledge Source Server into the computer-based patient record.* Proc AMIA Annu Fall Symp, 1997: p. 17-21.
5. Geissbuhler, A. and R.A. Miller, *Clinical application of the UMLS in a computerized order entry and decision-support system.* Proc AMIA Symp, 1998: p. 320-4.
6. van Mulligen, E.M., *UMLS-based access to CPR data.* Stud Health Technol Inform, 1998. **52 Pt 1**: p. 166-70.
7. van Mulligen, E.M., *UMLS-based access to CPR data. Unified Medical Language Systems.* Int J Med Inform, 1999. **53**(2-3): p. 125-31.
8. Boxwala, A.A., et al., *Coverage of patient safety terms in the UMLS metathesaurus.* AMIA Annu Symp Proc, 2003: p. 110-4.
9. Coonan, K.M., *Medical Informatics Standards Applicable to Emergency Department Information Systems: Making Sense of the Jumble.* Academic Emergency Medicine, 2004. **11**(11): p. 1198-205.
10. Currie, L.M., et al., *Development and representation of a fall-injury risk assessment instrument in a clinical information system.* Stud Health Technol Inform, 2004. **107**(Pt 1): p. 721-5.
11. Kawamoto, K. and D.F. Lobach, *Design, implementation, use, and preliminary evaluation of an UMLS-enabled terminology Web service for clinical decision support.* AMIA Annu Symp Proc, 2006: p. 979.
12. Tolentino, H., et al., *Concept negation in free text components of vaccine safety reports.* AMIA Annu Symp Proc, 2006: p. 1122.
13. Tolentino, H.D., et al., *A UMLS-based spell checker for natural language processing in vaccine safety.* BMC Med Inform Decis Mak, 2007. **7**: p. 3.
14. Lee, J.H., et al., *Effectiveness of automatic acute stroke alert system based on UMLS mapped local terminology codes at emergency department.* AMIA Annu Symp Proc, 2008: p. 1018.
15. Pires, D.F., C.A.C. Teixeira, and E.E.S. Ruiz, *A UMLS interoperable solution to support collaborative diagnosis decision making over the internet*, in *Proceedings of the 2008 ACM symposium on Applied computing*. 2008, Association for Computing Machinery: Fortaleza, Ceara, Brazil. p. 1400–1404.
16. Hashmi, Z., T. Zrimec, and A. Hopkins, *Computerization framework for clinical practice guidelines by extending the XML guidelines element model (GEM).* Stud Health Technol Inform, 2009. **150**: p. 638-42.
17. Bodenreider, O. and D.D. Fushman, *Investigating drug classes in biomedical terminologies from the perspective of clinical decision support.* AMIA Annu Symp Proc, 2010. **2010**: p. 56-60.
18. Fujita, H., J. Hakura, and M. Kurematsu, *Virtual doctor system (VDS): medical decision reasoning based on physical and mental ontologies*, in *Proceedings of the 23rd international conference on Industrial engineering and other applications of applied intelligent systems - Volume Part III*. 2010, Springer-Verlag: Cordoba, Spain. p. 419–428.
19. Bonacin, R., C. Pruski, and M.D. Silveira, *Careflow personalization services: concepts and tool for the evaluation of computer-interpretable guidelines*, in *Proceedings of the 3rd international conference on Knowledge Representation for Health-Care*. 2011, Springer-Verlag: Bled, Slovenia. p. 80–93.
20. McCoy, A.B., et al., *A prototype knowledge base and SMART app to facilitate organization of patient medications by clinical problems.* AMIA Annu Symp Proc, 2011. **2011**: p. 888-94.

21. Lehman, L.W., et al., *Risk stratification of ICU patients using topic models inferred from unstructured progress notes.* AMIA Annu Symp Proc, 2012. **2012**: p. 505-11.
22. Sánchez-Garzón, I., J. Fdez-Olivares, and L. Castillo, *An approach for representing and managing medical exceptions in care pathways based on temporal hierarchical planning techniques*, in *Proceedings of the 2012 international conference on Process Support and Knowledge Representation in Health Care*. 2012, Springer-Verlag: Tallinn, Estonia. p. 168–182.
23. Winnenburg, R. and O. Bodenreider, *Issues in creating and maintaining value sets for clinical quality measures.* AMIA Annu Symp Proc, 2012. **2012**: p. 988-96.
24. Patil, M.A., et al., *Estimating personalized risk ranking using laboratory test and medical knowledge (UMLS).* Conf Proc IEEE Eng Med Biol Soc, 2013. **2013**: p. 1274-7.
25. Valkenhoef, G.V., et al., *ADDIS: A decision support system for evidence-based medicine.* Decis. Support Syst., 2013. **55**(2): p. 459–475.
26. MacKellar, B., C. Schweikert, and S.A. Chun, *Patient-Centered Clinical Trials Decision Support using Linked Open Data.* Int. J. Softw. Sci. Comput. Intell., 2014. **6**(3): p. 31–48.
27. Kuang, J., et al., *Representation of Functional Status Concepts from Clinical Documents and Social Media Sources by Standard Terminologies.* AMIA Annu Symp Proc, 2015. **2015**: p. 795-803.
28. Shivade, C., et al., *Comparison of UMLS terminologies to identify risk of heart disease using clinical notes.* J Biomed Inform, 2015. **58 Suppl**: p. S103-10.
29. Varghese, J., S. Schulze Sunninghausen, and M. Dugas, *Standardized Cardiovascular Quality Assurance Forms with Multilingual Support, UMLS Coding and Medical Concept Analyses.* Stud Health Technol Inform, 2015. **216**: p. 837-41.
30. Varghese, J., S.S. Sunninghausen, and M. Dugas, *Standardized quality assurance forms for organ transplantations with multilingual support, open access and UMLS coding.* Stud Health Technol Inform, 2015. **212**: p. 15-22.
31. Choong, M.K., et al., *Linking clinical quality indicators to research evidence - a case study in asthma management for children.* BMC Health Serv Res, 2017. **17**(1): p. 502.
32. Moreira, A., et al., *Enhancing Collaborative Case Diagnoses Through Unified Medical Language System-Based Disambiguation: A Case Study of the Zika Virus.* Telemed J E Health, 2017. **23**(7): p. 608-614.
33. Bakal, G., et al., *Exploiting semantic patterns over biomedical knowledge graphs for predicting treatment and causative relations.* J Biomed Inform, 2018. **82**: p. 189-199.
34. Le, D.V., et al., *Risk prediction using natural language processing of electronic mental health records in an inpatient forensic psychiatry setting.* J Biomed Inform, 2018. **86**: p. 49-58.
35. Modaresnezhad, M., et al., *A rule-based semantic approach for data integration, standardization and dimensionality reduction utilizing the UMLS: Application to predicting bariatric surgery outcomes.* Comput Biol Med, 2019. **106**: p. 84-90.
