# Supplementary material for: The Unified Medical Language System at 30 Years and How It Is Used and Published: Systematic Review and Content Analysis
Source: JMIR Med Inform. 2021 Aug 27;9(8):e20675. doi: 10.2196/20675 (PMC8433943; doi:10.2196/20675)
Supplement: Multimedia Appendix 11 [file medinform_v9i8e20675_app11.pdf]

Multimedia Appendix 11. Unified Medical Language System publications about terminology studies.

Author	Publication year	Title	What was UMLS used for?
**Comparison of terminologies**			
Campbell, et al[1]	1994	A comparison of four schemes for codification of problem lists	UMLS, SNOMED, Read, ICD-9-CM, problem list
Campbell, et al[2]	1997	Phase II evaluation of clinical coding schemes: completeness, taxonomy, mapping, definitions, and clarity. CPRI Work Group on Codes and Structures	Clinical coding schemes, completeness, taxonomy, mapping, clarity, definitions, READ, SNOMED CT, UMLS
Strang, et al[3]	2002	Which coding system for therapeutic information in evidence-based medicine	Coding systems, therapeutic information, evaluation
De Coronado, et al[4]	2007	Using the UMLS Semantic Network to validate NCI Thesaurus structure and analyze its alignment with the OBO relations ontology	Validate NCI Thesaurus via UMLS, alignment of UMLS with OBO
Lezcano, et al[5]	2012	Associating clinical archetypes through UMLS Metathesaurus term clusters	Archetype, term cluster from UMLS, graph connectivity measures, association identification
Mougin, et al[6]	2012	Comparing drug-class membership in ATC and NDF-RT	The Anatomical Therapeutic Chemical (ATC), NDF-RT, mapping via UMLS
**Construction of terminology/taxonomy**			
Carenini, et al[7]	1993	Using the UMLS Semantic Network as a basis for constructing a terminological knowledge base: a preliminary report	UMLS, terminological knowledge base
Payne, et al[8]	1993	How useful is the UMLS metathesaurus in developing a controlled vocabulary for an automated problem list?	UMLS, controlled vocabulary, problem list
Volot, et al[9]	1993	Structuration and acquisition of medical knowledge. Using UMLS in the conceptual graph formalism	Concept type lattice (CTL) of Conceptual Graphs, UMLS
Burgun, et al[10]	1998	A collaborative approach to building a terminology for medical procedures using a Web-based application: from specifications to daily use	Terminology for medical procedures, MAOUSSC
Dessena, et al[11]	1998	Building cross-thesauri with the support of UMLS	Cross-thesauri, terminological corpora

Hales, et al[12]	1998	Extracting medical knowledge for a coded problem list vocabulary from the UMLS Knowledge Sources	Coded problem list, UMLS, knowledge extraction
Tuttle, et al[13]	1998	Metaphrase: an aid to the clinical conceptualization and formalization of patient problems in healthcare enterprises	Metaphrase, clinical conceptuation, clinical formalization
Schulz, et al[14]	2001	Medical knowledge reengineering--converting major portions of the UMLS into a terminological knowledge base	UMLS, terminological knowledge base, anatomy, pathology
Carter, et al[15]	2002	Initializing the VA medication reference terminology using UMLS metathesaurus co-occurrences	VA medication reference terminology, medication and diseases
Berman[16]	2003	A tool for sharing annotated research data: the "Category 0" UMLS (Unified Medical Language System) vocabularies	Sharing annotated research data, UMLS
Travers, et al[17]	2003	Using nurses' natural language entries to build a concept-oriented terminology for patients' chief complaints in the emergency department	Chief complaints, emergency department, NLP, UMLS, nursing vocabulary
Tse, et al[18]	2003	Exploring medical expressions used by consumers and the media: an emerging view of consumer health vocabularies	Consumer health vocabulary, UMLS,
Asbeh, et al[19]	2006	Creating consistent diagnoses list for developmental disorders using UMLS	Diagnoses list for developmental disorders, UMLS
Handler, et al[20]	2006	Improving a UMLS based allergy list for use in live electronic medical record systems	UMLS-based allergy list, SNOMED CT
Woods, et al[21]	2006	Using UMLS metathesaurus concepts to describe medical images: dermatology vocabulary	Image description, dermatology vocabulary
Keselman, et al[22]	2008	Consumer health concepts that do not map to the UMLS: where do they fit?	Consumer health vocabulary, UMLS
Fung, et al[23]	2010	The UMLS-CORE project: a study of the problem list terminologies used in large healthcare institutions	Problem list, UMLS, mapping
Matney, et al[24]	2012	Development of the nursing problem list subset of SNOMED	Nursing problem list, terminology subset, SNOMED CT
He, et al[25]	2016	Topological-Pattern-Based Recommendation of UMLS Concepts for National Cancer Institute Thesaurus	UMLS to enrich NCItUMLS
**Harmonization**			

Cimino, et al[26]	1993	From ICD9-CM to MeSH using the UMLS: a how-to guide	ICD9-CM to MeSH via UMLS, terms conversion
Levesque, et al[27]	1994	MD Concept: a model for integrating medical knowledge	MD concept, terminology integration
Rindflesch, et al[28]	1994	Ambiguity resolution while mapping free text to the UMLS Metathesaurus	Ambiguity resolution, mapping free text to UMLS concepts
Tuttle, et al[29]	1995	Merging terminologies	Merging terminologies, UMLS
Zeng, et al[30]	1996	Mapping medical vocabularies to the Unified Medical Language System	Mapping medical vocabularies to UMLS, automated term translation
Bodenreider, et al[31]	1998	Beyond synonymy: exploiting the UMLS semantics in mapping vocabularies	Mapping, UMLS, synonymy, inconsistency
Dessena, et al[32]	1999	Development of a cross-thesaurus with Internet-based refinement supported by UMLS	Cross-thesaurus, UMLS, refinement
Mendonca, et al[33]	1999	Evaluation of the Information Sources Map	UMLS, map different terminologies, indexing, connection of sources
Pisanelli, et al[34]	1999	A Medical Ontology Library That Integrates<br>the UMLS MetathesaurusTM	Ontological analysis, UMLS, integration
Burgun, et al[35]	2001	Mapping the UMLS Semantic Network into general ontologies	UMLS, mapping, ontology
Leroy, et al[36]	2001	Meeting medical terminology needs--the Ontology-Enhanced Medical Concept Mapper	Medical Concept Mapper, UMLS, WordNet, concept space, deep semantic parsing
Cantor, et al[37]	2003	An evaluation of hybrid methods for matching biomedical terminologies: mapping the gene ontology to the UMLS	Mapping from gene ontology to UMLS, evaluation
Sarkar, et al[38]	2003	Linking biomedical language information and knowledge resources: GO and UMLS	UMLS, GO, linking information and resources
Lomax, et al[39]	2004	Mapping the Gene Ontology into the Unified Medical Language System: Research Papers	Mapping GO and UMLS
Lussier[40]	2004	Terminological mapping for high throughput comparative biology of phenotypes	SNOMED CT, terminological mapping, phenotypes

Ruan, et al[41]	2004	Mapping various information sources to a semantic network	Drug information resources mapping, UMLS
Fung, et al[42]	2005	Integrating SNOMED CT into the UMLS: an exploration of different views of synonymy and quality of editing	Integration of SNOMED CT into UMLS
Fung, et al[43]	2005	Utilizing the UMLS for semantic mapping between terminologies	Semantic mapping, UMLS, quality assurance
Nachimuthu, et al[44]	2005	Applying hybrid algorithms for text matching to automated biomedical vocabulary mapping	Text matching, automatic biomedical vocabulary mapping
Mougin, et al[45]	2006	Using WordNet to improve the mapping of data elements to UMLS for data sources integration	Mapping of data elements to UMLS, data sources integration, WordNet
Sun, et al[46]	2006	A system for automated lexical mapping	Automated lexical mapping, UMLS, LOINC
Fung, et al[47]	2007	Combining lexical and semantic methods of inter-terminology mapping using the UMLS	Mapping, UMLS, SNOMED CT, ICD9CM
Hishiki, et al[48]	2007	Linking the clinical vocabulary of diseases to the genes by mapping UMLS to OMIM allelic variant fields	Mapping UMLS to OMIM, linking clinical vocabulary of diseases to gene
Huang, et al[49]	2007	Piecewise synonyms for enhanced UMLS source terminology integration	Piecewise synonyms, UMLS sources integration
Patel, et al[50]	2007	A scale-free network view of the UMLS to learn terminology translations	Terminology mapping via UMLS
Patel, et al[51]	2007	Decompositional terminology translation using network analysis	Decompositional terminology, mapping
Taboada, et al[52]	2007	Effectiveness Study of Lexically Mapping Two Thesauri	Mapping thesauri,
Weng, et al[53]	2007	User-centered semantic harmonization: a case study	Semantic interoperability, metadata, ontology alignment
Andreopoulos, et al[54]	2008	Integration of Genomic, Proteomic and Biomedical Information on the Semantic Web	Integrating ontologies and vocabularies, GO, UMLS
Mehan, et al[55]	2008	An integrative network approach to map the transcriptome to the phenome	Mapping of gene coexpression to phenotypes, Gene Ontology, GeneRIF and UMLS

Mougin, et al[56]	2008	Automatic Methods for Integrating Biomedical Data Sources in a Mediator-Based System	Heterogeneous sources, Integration systems, global schema from the UMLS
Shamdasani, et al[57]	2008	Semantic Matching for the Medical Domain	Match algorithm, semantic matching of medical terminologies via UMLS
Wang, et al[58]	2008	A computational linguistics motivated mapping of ICPC-2 PLUS to SNOMED CT	Automatic mapping from ICPC-2 PLUS to SNOMED CT via UMLS
Schulz, et al [59]	2009	Alignment of the UMLS semantic network with BioTop: methodology and assessment	Alignment between UMLS semantic network and BioTop, OWL DL
Taboada, et al[60]	2009	An automated approach to mapping external terminologies to the UMLS	Automatic mapping, UMLS, similar strings, lexical alignment
Carlo, et al[61]	2010	Aligning Structured and Unstructured Medical Problems Using UMLS	Mapping, medical problems, UMLS
Nadkarni, et al[62]	2010	Determining correspondences between high-frequency MedDRA concepts and SNOMED: a case study	MedDRA, SNOMED, mapping, integration error
Yip, et al[63]	2010	Concept integration from the caTIES to i2b2 using the UMLS semantic network	Cancer Text Information Extraction System (caTIES) to i2b2, concept integration, UMLS
Meizoso, et al[64]	2011	Automated mapping of observation archetypes to SNOMED CT concepts	Automated mapping between open-EHR Archetypes and SNOMED CT, UMLS
Mougin, et al[65]	2011	Improving the mapping between MedDRA and SNOMED CT	Mapping MedDRA and SNOMED CT via UMLS
Griffon, et al[66]	2014	Evaluating alignment quality between iconic language and reference terminologies using similarity metrics	Similarity metrics, alignment quality, mapping quality
He, et al[67]	2015	A comparative analysis of the density of the SNOMED CT conceptual content for semantic harmonization	semantic harmonization, terminology mapping, integration
Alnazzavi, et al[68]	2016	Mapping Phenotypic Information in Heterogeneous Textual Sources to a Domain-Specific Terminological Resource	Automatic link of phenotype and UMLS concepts, mapping
Kim[69]	2016	Automating lexical cross-mapping of ICNP to SNOMED CT	Automating lexical mapping, ICNP to SNOMED CT
Storck, et al[70]	2016	ODMSummary: A Tool for Automatic Structured Comparison of Multiple	Semantic annotation with UMLS, structured

		Medical Forms Based on Semantic Annotation with the Unified Medical Language System	comparison, multiple medical forms
Cuzzola, et al[71]	2018	UMLS to DBPedia link discovery through circular resolution	Linking UMLS concepts to DBpedia resources
**Interoperability**			
Tuttle, et al[72]	1993	Toward an interim standard for patient-centered knowledge-access	Patient-centered knowledge-access, knowledge needs
Aymard, et al[73]	1998	Towards interoperability of information sources within a hospital Intranet	Interoperability, ARIANE, information sources, UMLS
Ingenerf, et al[74]	2001	Standardized terminological services enabling semantic interoperability between distributed and heterogeneous systems	UMLS, interoperability, standard terminology, MUSTANG system
Liu[75]	2007	Enabling Electronic Healthcare Information Exchange	Standardized representation, UMLS, interoperability
Warnekar, et al[76]	2007	Use of RxNorm to exchange codified drug allergy information between Department of Veterans Affairs (VA) and Department of Defense (DoD)	Standardized and codified drug allergy information, sharing, UMLS, RxNorm
Richesson, et al[77]	2008	Heterogeneous but "standard" coding systems for adverse events: Issues in achieving interoperability between apples and oranges	Coding systems for adverse events, interoperability
Dugas, et al[78]	2013	Automated UMLS-based comparison of medical forms	Automated medical form comparison, harmonization, interoperability
**Quality assurance**			
Gu[79]	1999	Developing techniques for enhancing comprehensibility of controlled medical terminologies	Comprehensibility of controlled medical terminologies
Bodenreider, et al[80]	2002	Assessing the consistency of a biomedical terminology through lexical knowledge	Biomedical terminology, consistency, similar lexical
Bodenreider[81]	2003	Strength in numbers: exploring redundancy in hierarchical relations across biomedical terminologies	Redundancy, UMLS, terminology overlap, consistency, auditing
Vizenor, et al[82]	2009	Auditing associative relations across two knowledge sources	Auditing associative relationships, consistency, UMLS
Jiang, et al[83]	2011	Quality evaluation of cancer study Common Data Elements using the UMLS Semantic Network	Cancer, common data elements, quality assurance

Adamusiak, et al[84]	2012	Quality assurance in LOINC using Description Logic	LOINC, SNOMED CT mapping via UMLS
Jiang, et al[85]	2012	Quality evaluation of value sets from cancer study common data elements using the UMLS semantic groups	Quality of value set, cancer, common data elements
**Other publications of terminology**			
Joubert, et al[86]	1994	Users conceptual views on medical information databases	Conceptual graph, database queries, semantic network, UMLS as an example
Hahn, et al[87]	2000	Towards Very Large Terminological Knowledge Bases: A Case Study from Medicine	Informal medical thesaurus (UMLS), automatically convert, sound description logics system
Geller, et al[88]	2002	Evaluation and application of a semantic network partition	Semantic network partition, UMLS, partial view
Hahn, et al[89]	2002	Turning Lead into Gold? Feeding a Formal Knowledge Base with Informal Conceptual Knowledge	Formal knowledge base, informal conceptual knowledge
Bales, et al[90]	2007	Topological analysis of large-scale biomedical terminology structures	Topological analysis, terminology structure

References

1. Campbell, J.R. and T.H. Payne, *A comparison of four schemes for codification of problem lists.* Proc Annu Symp Comput Appl Med Care, 1994: p. 201-5.
2. Campbell, J.R., et al., *Phase II evaluation of clinical coding schemes: completeness, taxonomy, mapping, definitions, and clarity. CPRI Work Group on Codes and Structures.* J Am Med Inform Assoc, 1997. **4**(3): p. 238-51.
3. Strang, N., M. Cucherat, and J.P. Boissel, *Which coding system for therapeutic information in evidence-based medicine.* Comput Methods Programs Biomed, 2002. **68**(1): p. 73-85.
4. de Coronado, S., M.S. Tuttle, and H.R. Solbrig, *Using the UMLS Semantic Network to validate NCI Thesaurus structure and analyze its alignment with the OBO relations ontology.* AMIA Annu Symp Proc, 2007: p. 165-70.
5. Lezcano, L., S. Sanchez-Alonso, and M.A. Sicilia, *Associating clinical archetypes through UMLS Metathesaurus term clusters.* J Med Syst, 2012. **36**(3): p. 1249-58.
6. Mougin, F., A. Burgun, and O. Bodenreider, *Comparing drug-class membership in ATC and NDF-RT*, in *Proceedings of the 2nd ACM SIGHIT International Health Informatics Symposium*. 2012, Association for Computing Machinery: Miami, Florida, USA. p. 437–444.
7. Carenini, G. and J.D. Moore, *Using the UMLS Semantic Network as a basis for constructing a terminological knowledge base: a preliminary report.* Proc Annu Symp Comput Appl Med Care, 1993: p. 725-9.
8. Payne, T.H. and D.R. Martin, *How useful is the UMLS metathesaurus in developing a controlled vocabulary for an automated problem list?* Proc Annu Symp Comput Appl Med Care, 1993: p. 705-9.

9. Volot, F., et al., *Structuration and acquisition of medical knowledge. Using UMLS in the conceptual graph formalism.* Proc Annu Symp Comput Appl Med Care, 1993: p. 710-4.
10. Burgun, A., et al., *A collaborative approach to building a terminology for medical procedures using a Web-based application: from specifications to daily use.* Stud Health Technol Inform, 1998. **52 Pt 1**: p. 596-9.
11. Dessena, S., A. Rossi Mori, and E. Galeazzi, *Building cross-thesauri with the support of UMLS.* Stud Health Technol Inform, 1998. **52 Pt 1**: p. 654-9.
12. Hales, J.W., K.M. Schoeffler, and D.P. Kessler, *Extracting medical knowledge for a coded problem list vocabulary from the UMLS Knowledge Sources.* Proc AMIA Symp, 1998: p. 275-9.
13. Tuttle, M.S., et al., *Metaphrase: an aid to the clinical conceptualization and formalization of patient problems in healthcare enterprises.* Methods Inf Med, 1998. **37**(4-5): p. 373-83.
14. Schulz, S. and U. Hahn, *Medical knowledge reengineering--converting major portions of the UMLS into a terminological knowledge base.* Int J Med Inform, 2001. **64**(2-3): p. 207-21.
15. Carter, J.S., et al., *Initializing the VA medication reference terminology using UMLS metathesaurus co-occurrences.* Proc AMIA Symp, 2002: p. 116-20.
16. Berman, J.J., *A tool for sharing annotated research data: the "Category 0" UMLS (Unified Medical Language System) vocabularies.* BMC Med Inform Decis Mak, 2003. **3**: p. 6.
17. Travers, D.A. and S.W. Haas, *Using nurses' natural language entries to build a concept-oriented terminology for patients' chief complaints in the emergency department.* J Biomed Inform, 2003. **36**(4-5): p. 260-70.
18. Tse, T. and D. Soergel, *Exploring medical expressions used by consumers and the media: an emerging view of consumer health vocabularies.* AMIA Annu Symp Proc, 2003: p. 674-8.
19. Asbeh, N., et al., *Creating consistent diagnoses list for developmental disorders using UMLS*, in *Proceedings of the 6th international conference on Next Generation Information Technologies and Systems*. 2006, Springer-Verlag: Kibbutz Shefayim, Israel. p. 333–336.
20. Handler, J., et al., *Improving a UMLS based allergy list for use in live electronic medical record systems.* AMIA Annu Symp Proc, 2006: p. 942.
21. Woods, J.W., et al., *Using UMLS metathesaurus concepts to describe medical images: dermatology vocabulary.* Comput Biol Med, 2006. **36**(1): p. 89-100.
22. Keselman, A., et al., *Consumer health concepts that do not map to the UMLS: where do they fit?* J Am Med Inform Assoc, 2008. **15**(4): p. 496-505.
23. Fung, K.W., C. McDonald, and S. Srinivasan, *The UMLS-CORE project: a study of the problem list terminologies used in large healthcare institutions.* J Am Med Inform Assoc, 2010. **17**(6): p. 675-80.
24. Matney, S.A., et al., *Development of the nursing problem list subset of SNOMED CT(R).* J Biomed Inform, 2012. **45**(4): p. 683-8.
25. He, Z., et al., *Topological-Pattern-Based Recommendation of UMLS Concepts for National Cancer Institute Thesaurus.* AMIA Annu Symp Proc, 2016. **2016**: p. 618-627.
26. Cimino, J.J., et al., *From ICD9-CM to MeSH using the UMLS: a how-to guide.* Proc Annu Symp Comput Appl Med Care, 1993: p. 730-4.
27. Levesque, Y., A.R. LeBlanc, and M. Maksud, *MD Concept: a model for integrating medical knowledge.* Proc Annu Symp Comput Appl Med Care, 1994: p. 252-6.
28. Rindflesch, T.C. and A.R. Aronson, *Ambiguity resolution while mapping free text to the UMLS Metathesaurus.* Proc Annu Symp Comput Appl Med Care, 1994: p. 240-4.
29. Tuttle, M.S., et al., *Merging terminologies.* Medinfo, 1995. **8 Pt 1**: p. 162-6.
30. Zeng, Q. and J.J. Cimino, *Mapping medical vocabularies to the Unified Medical Language System.* Proc AMIA Annu Fall Symp, 1996: p. 105-9.

31. Bodenreider, O., et al., *Beyond synonymy: exploiting the UMLS semantics in mapping vocabularies.* Proc AMIA Symp, 1998: p. 815-9.
32. Dessena, S., A.R. Mori, and E. Galeazzi, *Development of a cross-thesaurus with Internet-based refinement supported by UMLS.* Int J Med Inform, 1999. **53**(1): p. 29-41.
33. Mendonca, E.A. and J.J. Cimino, *Evaluation of the Information Sources Map.* Proc AMIA Symp, 1999: p. 873-7.
34. Pisanelli, D.M., A. Gangemi, and G. Steve, *A Medical Ontology Library That Integrates the UMLS MetathesaurusTM*, in *Proceedings of the Joint European Conference on Artificial Intelligence in Medicine and Medical Decision Making*. 1999, Springer-Verlag. p. 239–248.
35. Burgun, A. and O. Bodenreider, *Mapping the UMLS Semantic Network into general ontologies.* Proc AMIA Symp, 2001: p. 81-5.
36. Leroy, G. and H. Chen, *Meeting medical terminology needs--the Ontology-Enhanced Medical Concept Mapper.* IEEE Trans Inf Technol Biomed, 2001. **5**(4): p. 261-70.
37. Cantor, M.N., et al., *An evaluation of hybrid methods for matching biomedical terminologies: mapping the gene ontology to the UMLS.* Stud Health Technol Inform, 2003. **95**: p. 62-7.
38. Sarkar, I.N., et al., *Linking biomedical language information and knowledge resources: GO and UMLS.* Pac Symp Biocomput, 2003: p. 439-50.
39. Lomax, J. and A.T. McCray, *Mapping the Gene Ontology into the Unified Medical Language System: Research Papers.* Comp. Funct. Genomics, 2004. **5**(4): p. 354–361.
40. Lussier, Y.A. and J. Li, *Terminological mapping for high throughput comparative biology of phenotypes.* Pac Symp Biocomput, 2004: p. 202-13.
41. Ruan, W., T. Buerkle, and J.W. Dudeck, *Mapping various information sources to a semantic network.* Stud Health Technol Inform, 2004. **107**(Pt 1): p. 430-3.
42. Fung, K.W., et al., *Integrating SNOMED CT into the UMLS: an exploration of different views of synonymy and quality of editing.* J Am Med Inform Assoc, 2005. **12**(4): p. 486-94.
43. Fung, K.W. and O. Bodenreider, *Utilizing the UMLS for semantic mapping between terminologies.* AMIA Annu Symp Proc, 2005: p. 266-70.
44. Nachimuthu, S.K. and L.M. Lau, *Applying hybrid algorithms for text matching to automated biomedical vocabulary mapping.* AMIA Annu Symp Proc, 2005: p. 555-9.
45. Mougin, F., A. Burgun, and O. Bodenreider, *Using WordNet to improve the mapping of data elements to UMLS for data sources integration.* AMIA Annu Symp Proc, 2006: p. 574-8.
46. Sun, J.Y. and Y. Sun, *A system for automated lexical mapping.* J Am Med Inform Assoc, 2006. **13**(3): p. 334-43.
47. Fung, K.W., et al., *Combining lexical and semantic methods of inter-terminology mapping using the UMLS.* Stud Health Technol Inform, 2007. **129**(Pt 1): p. 605-9.
48. Hishiki, T. and I. Tamada, *Linking the clinical vocabulary of diseases to the genes by mapping UMLS to OMIM allelic variant fields.* AMIA Annu Symp Proc, 2007: p. 976.
49. Huang, K.C., et al., *Piecewise synonyms for enhanced UMLS source terminology integration.* AMIA Annu Symp Proc, 2007: p. 339-43.
50. Patel, C.O. and J.J. Cimino, *A scale-free network view of the UMLS to learn terminology translations.* Stud Health Technol Inform, 2007. **129**(Pt 1): p. 689-93.
51. Patel, C.O. and J.J. Cimino, *Decompositional terminology translation using network analysis.* AMIA Annu Symp Proc, 2007: p. 588-92.
52. Taboada, M., et al., *Effectiveness Study of Lexically Mapping Two Thesauri*, in *Current Topics in Artificial Intelligence: 12th Conference of the Spanish Association for Artificial Intelligence, CAEPIA 2007, Salamanca, Spain, November 12-16, 2007. Selected Papers*. 2007, Springer-Verlag. p. 268–277.

53. Weng, C., J.H. Gennari, and D.B. Fridsma, *User-centered semantic harmonization: a case study.* J Biomed Inform, 2007. **40**(3): p. 353-64.
54. Andreopoulos, B., et al., *Integration of Genomic, Proteomic and Biomedical Information on the Semantic Web*, in *Proceedings of the ER 2008 Workshops (CMLSA, ECDM, FP-UML, M2AS, RIGiM, SeCoGIS, WISM) on Advances in Conceptual Modeling: Challenges and Opportunities*. 2008, Springer-Verlag: Barcelona, Spain. p. 33–42.
55. Mehan, M.R., et al., *An integrative network approach to map the transcriptome to the phenome*, in *Proceedings of the 12th annual international conference on Research in computational molecular biology*. 2008, Springer-Verlag: Singapore. p. 232–245.
56. Mougin, F., et al., *Automatic Methods for Integrating Biomedical Data Sources in a Mediator-Based System*, in *Proceedings of the 5th international workshop on Data Integration in the Life Sciences*. 2008, Springer-Verlag: Evry, France. p. 61–76.
57. Shamdasani, J., P. Bloodsworth, and R. Mcclatchey, *Semantic Matching for the Medical Domain*, in *Proceedings of the 25th British national conference on Databases: Sharing Data, Information and Knowledge*. 2008, Springer-Verlag: Cardiff, Wales, UK. p. 198–202.
58. Wang, Y., et al., *A computational linguistics motivated mapping of ICPC-2 PLUS to SNOMED CT.* BMC Med Inform Decis Mak, 2008. **8 Suppl 1**: p. S5.
59. Schulz, S., et al., *Alignment of the UMLS semantic network with BioTop: methodology and assessment.* Bioinformatics, 2009. **25**(12): p. i69-76.
60. Taboada, M., R. Lalin, and D. Martinez, *An automated approach to mapping external terminologies to the UMLS.* IEEE Trans Biomed Eng, 2009. **56**(6): p. 1598-605.
61. Carlo, L., H.S. Chase, and C. Weng, *Aligning Structured and Unstructured Medical Problems Using UMLS.* AMIA Annu Symp Proc, 2010. **2010**: p. 91-5.
62. Nadkarni, P.M. and J.D. Darer, *Determining correspondences between high-frequency MedDRA concepts and SNOMED: a case study.* BMC Med Inform Decis Mak, 2010. **10**: p. 66.
63. Yip, V. and U. Topaloglu, *Concept integration from the caTIES to i2b2 using the UMLS semantic network*, in *Proceedings of the 1st ACM International Health Informatics Symposium*. 2010, Association for Computing Machinery: Arlington, Virginia, USA. p. 366–370.
64. Meizoso, M., et al., *Automated mapping of observation archetypes to SNOMED CT concepts*, in *Proceedings of the 4th international conference on Interplay between natural and artificial computation - Volume Part I*. 2011, Springer-Verlag: Canary Islands, Spain. p. 550–561.
65. Mougin, F., M. Dupuch, and N. Grabar, *Improving the mapping between MedDRA and SNOMED CT*, in *Proceedings of the 13th conference on Artificial intelligence in medicine*. 2011, Springer-Verlag: Bled, Slovenia. p. 220–224.
66. Griffon, N., et al., *Evaluating alignment quality between iconic language and reference terminologies using similarity metrics.* BMC Med Inform Decis Mak, 2014. **14**: p. 17.
67. He, Z., J. Geller, and Y. Chen, *A comparative analysis of the density of the SNOMED CT conceptual content for semantic harmonization.* Artif Intell Med, 2015. **64**(1): p. 29-40.
68. Alnazzawi, N., P. Thompson, and S. Ananiadou, *Mapping Phenotypic Information in Heterogeneous Textual Sources to a Domain-Specific Terminological Resource.* PLoS One, 2016. **11**(9).
69. Kim, T.Y., *Automating lexical cross-mapping of ICNP to SNOMED CT.* Inform Health Soc Care, 2016. **41**(1): p. 64-77.
70. Storck, M., R. Krumm, and M. Dugas, *ODMSummary: A Tool for Automatic Structured Comparison of Multiple Medical Forms Based on Semantic Annotation with the Unified Medical Language System.* PLoS One, 2016. **11**(10): p. e0164569.
71. Cuzzola, J., E. Bagheri, and J. Jovanovic, *UMLS to DBPedia link discovery through circular resolution.* J Am Med Inform Assoc, 2018. **25**(7): p. 819-826.

72. Tuttle, M.S., et al., *Toward an interim standard for patient-centered knowledge-access.* Proc Annu Symp Comput Appl Med Care, 1993: p. 564-8.
73. Aymard, S., et al., *Towards interoperability of information sources within a hospital Intranet.* Proc AMIA Symp, 1998: p. 638-42.
74. Ingenerf, J., J. Reiner, and B. Seik, *Standardized terminological services enabling semantic interoperability between distributed and heterogeneous systems.* Int J Med Inform, 2001. **64**(2-3): p. 223-40.
75. Liu, S., *Enabling Electronic Healthcare Information Exchange.* IT Professional, 2007. **9**(6): p. 17–23.
76. Warnekar, P.P., et al., *Use of RxNorm to exchange codified drug allergy information between Department of Veterans Affairs (VA) and Department of Defense (DoD).* AMIA Annu Symp Proc, 2007: p. 781-5.
77. Richesson, R.L., K.W. Fung, and J.P. Krischer, *Heterogeneous but "standard" coding systems for adverse events: Issues in achieving interoperability between apples and oranges.* Contemp Clin Trials, 2008. **29**(5): p. 635-45.
78. Dugas, M., et al., *Automated UMLS-Based Comparison of Medical Forms.* PLoS One, 2013. **8**(7).
79. Gu, H., *Developing techniques for enhancing comprehensibility of controlled medical terminologies*. 1999, New Jersey Institute of Technology.
80. Bodenreider, O., A. Burgun, and T.C. Rindflesch, *Assessing the consistency of a biomedical terminology through lexical knowledge.* Int J Med Inform, 2002. **67**(1-3): p. 85-95.
81. Bodenreider, O., *Strength in numbers: exploring redundancy in hierarchical relations across biomedical terminologies.* AMIA Annu Symp Proc, 2003: p. 101-5.
82. Vizenor, L.T., O. Bodenreider, and A.T. McCray, *Auditing associative relations across two knowledge sources.* J Biomed Inform, 2009. **42**(3): p. 426-39.
83. Jiang, G., H.R. Solbrig, and C.G. Chute, *Quality evaluation of cancer study Common Data Elements using the UMLS Semantic Network.* J Biomed Inform, 2011. **44 Suppl 1**: p. S78-85.
84. Adamusiak, T. and O. Bodenreider, *Quality assurance in LOINC using Description Logic.* AMIA Annu Symp Proc, 2012. **2012**: p. 1099-108.
85. Jiang, G., H.R. Solbrig, and C.G. Chute, *Quality evaluation of value sets from cancer study common data elements using the UMLS semantic groups.* J Am Med Inform Assoc, 2012. **19**(e1): p. e129-36.
86. Joubert, M., et al., *Users conceptual views on medical information databases.* Int J Biomed Comput, 1994. **37**(2): p. 93-104.
87. Hahn, U. and S. Schulz, *Towards Very Large Terminological Knowledge Bases: A Case Study from Medicine*, in *Proceedings of the 13th Biennial Conference of the Canadian Society on Computational Studies of Intelligence: Advances in Artificial Intelligence*. 2000, Springer-Verlag. p. 176–186.
88. Geller, J., et al., *Evaluation and application of a semantic network partition.* Trans. Info. Tech. Biomed., 2002. **6**(2): p. 109–115.
89. Hahn, U. and S. Schulz, *Turning Lead into Gold? Feeding a Formal Knowledge Base with Informal Conceptual Knowledge*, in *Proceedings of the 13th International Conference on Knowledge Engineering and Knowledge Management. Ontologies and the Semantic Web*. 2002, Springer-Verlag. p. 182–196.
90. Bales, M.E., Y.A. Lussier, and S.B. Johnson, *Topological analysis of large-scale biomedical terminology structures.* J Am Med Inform Assoc, 2007. **14**(6): p. 788-97.
