# Supplementary material for: The Unified Medical Language System at 30 Years and How It Is Used and Published: Systematic Review and Content Analysis
Source: JMIR Med Inform. 2021 Aug 27;9(8):e20675. doi: 10.2196/20675 (PMC8433943; doi:10.2196/20675)
Supplement: Multimedia Appendix 12 [file medinform_v9i8e20675_app12.pdf]

Multimedia Appendix 12. Publications about studies of the Unified Medical Language System itself.

Author	Publication year	Title	What was UMLS used for?
**Applications or tools for UMLS**			
Komorowski, et al[1]	1988	Browsing and authoring tools for a unified medical language system	UMLS browser, UMLS authoring tool
Barber, et al[2]	1992	Integrating the UMLS into VNS Retriever	UMLS Retriever, VNS Retriever architecture
Harbourt, et al[3]	1993	The ranking algorithm of the Coach browser for the UMLS metathesaurus	Coach browser, UMLS metathesaurus
McCray, et al[4]	1995	The UMLS Knowledge Source server	UMLS knowledge source server, a command-line application
Miller, et al[5]	1995	Lessons learned from a pilot implementation of the UMLS information sources map	UMLS information sources map, implementation, ISM KB and navigational tools
Tuttle, et al[6]	1995	Navigating to knowledge	UMLS, NCI Knowledge Server, user-friendly interface, knowledge navigation
McCray, et al[7]	1996	The UMLS Knowledge Source Server: a versatile Internet-based research tool	UMLS knowledge source server, the Web interface
Nadkarni[8]	1997	Concept locator: a client-server application for retrieval of UMLS metathesaurus concepts through complex boolean query	UMLS metathesaurus concept locator
McCray, et al[9]	1999	Terminology issues in user access to Web-based medical information	Users' queries, UMLS-based terminology server
Frankewitsch, et al[10]	2000	Graphical tool for navigation within the semantic network of the UMLS metathesaurus on a locally installed database	UMLS metathesaurus, navigation, visualization
Wang, et al[11]	2000	Versatile user interface using UMLS Metathesaurus	UMLS, interface, SHIRE Refined Search
Brandt, et al[12]	2001	Web-based UMLS concept retrieval by automatic text scanning: a comparison of two methods	UMLS concept retrieval, automatic text scanning

Bangalore, et al[13]	2003	The UMLS knowledge source server: an object model for delivering UMLS data	UMLS knowledge source server, API
Browne, et al[14]	2003	UMLS language and vocabulary tools	UMLS, lexical variations
Tao, et al[15]	2003	A "systematics" tool for medical terminologies	Visualization, navigation and editing tool, UMLS
Mirhaji, et al[16]	2005	A Web Services architecture for UMLS Knowledge Sources	UMLS Web Services
Bangalore, et al[17]	2006	UMLSKS SUGGEST: an auto-complete feature for the UMLSKS interface using AJAX	UMLS knowledge source, auto-completion
Thorn, et al[18]	2006	Plug-and-play UMLS knowledge source server using web services and portlets	UMLS source server, information retrieval
Thorn, et al[19]	2007	The UMLS Knowledge Source Server: an experience in Web 2.0 technologies	UMLS knowledge source server, Web 2.0
Aronson, et al[20]	2008	Methodology for creating UMLS content views appropriate for biomedical natural language processing	UMLS content view, NLP
Shah, et al[21]	2008	UMLS-Query: a perl module for querying the UMLS	UMLS query, Perl
Demner-Fushmen, et al[22]	2010	UMLS content views appropriate for NLP processing of the biomedical literature vs. clinical text	Automatic indexing, problem list extraction, automatic generation of a subset
Geller, et al[23]	2013	Rule-based support system for multiple UMLS semantic type assignments	UMLS quality assurance, rule-based support system
Neuhaus, et al[24]	2015	Standardized mappings--a framework to combine different semantic mappers into a standardized web-API	Automatic coding of medical terms, UMLS mapping, similarity search
Rance, et al[25]	2015	Fingerprinting Biomedical Terminologies--Automatic Classification and Visualization of Biomedical Vocabularies through UMLS Semantic Group Profiles	Automatic classification, automatic visualization, UMLS semantic group profiles
**Auditing of UMLS**			
Cimino[26]	1998	Auditing the Unified Medical Language System with semantic methods	UMLS, auditing, detection of ambiguity, inconsistency, redundancy
Gu, et al[27]	2002	Using the metaschema to audit UMLS classification errors	Metaschema, auditing, UMLS

			classification error, inconsistency
Peng, et al[28]	2002	Auditing the UMLS for redundant classifications	UMLS, auditing, redundant classification identification
Bodenreider, et al[29]	2003	Exploring semantic groups through visual approaches	Visual approaches, semantic coherence, auditing, validation
Cimino, et al[30]	2003	Consistency across the hierarchies of the UMLS Semantic Network and Metathesaurus	Automatic detection of inconsistency, UMLS
Gu, et al[31]	2004	Auditing concept categorizations in the UMLS	UMLS auditing, concept categorization
Gu, et al[32]	2007	Evaluation of a UMLS Auditing Process of Semantic Type Assignments	UMLS auditing, evaluation, semantic type
Chen, et al[33]	2009	Expanding the extent of a UMLS semantic type via group neighborhood auditing	UMLS expansion, group neighborhood auditing, missing semantic type identification
Chen, et al[34]	2009	Structural group auditing of a UMLS semantic type's extent	UMLS group auditing
Chen, et al[35]	2009	Structural group-based auditing of missing hierarchical relationships in UMLS	Missing hierarchical relationships, UMLS group-based auditing
Geller, et al[36]	2009	Comparing inconsistent relationship configurations indicating UMLS errors	Auditing inconsistency, UMLS
Morrey, et al[37]	2009	The Neighborhood Auditing Tool: a hybrid interface for auditing the UMLS	UMLS neighborhood auditing
Mougin, et al[38]	2009	Analyzing polysemous concepts from a clinical perspective: application to auditing concept categorization in the UMLS	Auditing concept categorization, UMLS, polysemous terms
Erdogan, et al[39]	2010	Exploiting UMLS semantics for checking semantic consistency among UMLS concepts	UMLS, semantic consistency
Erdogn, et al[40]	2010	Finding semantic inconsistencies in UMLS using answer set programming	Semantic inconsistency detection
Huang, et al[41]	2010	Auditing SNOMED Integration into the UMLS for Duplicate Concepts	Auditing, UMLS, duplicate concepts detection,
Rudniy, et al[42]	2010	Shortest Path Edit Distance for Enhancing UMLS Integration and Audit	UMLS integration, audit, string similarity measures

Halper, et al[43]	2011	Auditing hierarchical cycles to locate other inconsistencies in the UMLS	UMLS auditing, inconsistency detection, error detection
Morrey, et al[44]	2011	Resolution of redundant semantic type assignments for organic chemicals in the UMLS	UMLS organic chemistry, redundant semantic type identification
Ochs, et al[45]	2011	A RELATIONSHIP-CENTRIC HYBRID INTERFACE FOR BROWSING AND AUDITING THE UMLS	UMLS browser, auditing,
Gu, et al[46]	2012	A study of terminology auditors' performance for UMLS semantic type assignments	UMLS auditing
Wei, et al[47]	2012	Using SNOMED semantic concept groupings to enhance semantic-type assignment consistency in the UMLS	Semantic grouping, semantic-type assignment, consistency enhancement, UMLS
Mougin, et al[48]	2014	Auditing the multiply-related concepts within the UMLS	UMLS auditing, multiple relations concepts in UMLS
Gu, et al[49]	2018	Validating UMLS Semantic Type Assignments Using SNOMED CT Semantic Tags	Semantic type assignments, error detection, inconsistency, UMLS audit
**Components of UMLS and UMLS**			
Komorowski, et al[50]	1990	Knowledge modeling for the unified medical language system	UMLS, modeling
Humphreys, et al[51]	1991	Assessing and enhancing the value of the UMLS Knowledge Sources	UMLS knowledge sources, assessment and enhancement
Nelson, et al[52]	1991	From meaning to term: semantic locality in the UMLS Metathesaurus	UMLS Metathesaurus, semantic locality
Tuttle, et al[53]	1991	Adding your terms and relationships to the UMLS Metathesaurus	UMLS Metathesaurus enhancement
Yang, et al[54]	1991	A schematic analysis of the Unified Medical Language System	UMLS, schematic analysis
Lindberg, et al[55]	1992	The unified medical language system (UMLS) and computer-based patient records	UMLS, EMR
Masys[56]	1992	An evaluation of the source selection elements of the prototype UMLS Information Sources Map	UMLS information Sources Map, automated source selection

Nelson, et al[57]	1992	The semantic structure of the UMLS Metathesaurus	Contextual information, semantic types, co-occurrence terms
Sperzel, et al[58]	1992	The Meta-1.2 engine: a refined strategy for linking biomedical vocabularies	Meta-1.2 engine, linking UMLS sources
Tuttle, et al[59]	1992	The homogenization of the Metathesaurus schema and distribution format	Metathesaurus schema, UMLS distribution format
Clyman, et al[60]	1993	Using a network menu and the UMLS Information Sources Map to facilitate access to online reference materials	UMLS information sources map, online reference access
Humphreys, et al[61]	1993	The UMLS project: making the conceptual connection between users and the information they need	UMLS, conceptual connection, information needs
Humphreys, et al[62]	1993	The unified medical language system: moving beyond the vocabulary of bibliographic retrieval	UMLS
Lindberg, et al[63]	1993	The Unified Medical Language System	UMLS
McCray[64]	1993	Representing biomedical knowledge in the UMLS semantic network	UMLS, representation
Schuyler, et al[65]	1993	The UMLS Metathesaurus: representing different views of biomedical concepts	UMLS Metathesaurus
Tuttle, et al[66]	1994	The role of the UMLS in 'storing' and 'sharing' across systems	UMLS, reusing, sharing
Burgun, et al[67]	1995	Knowledge acquisition from the UMLS sources: application to the description of surgical procedures	Knowledge acquisition, UMLS sources, surgical procedures
Joubert, et al[68]	1995	A conceptual graphs modeling of UMLS components	UMLS graph modeling
McCray, et al[69]	1995	ASN.1: defining a grammar for the UMLS knowledge sources	UMLS knowledge sources
McCray, et al[70]	1995	The representation of meaning in the UMLS	UMLS metathesaurus and semantic network design principles
Bodenreider, et al[71]	1998	Evaluation of the Unified Medical Language System as a medical knowledge source	UMLS evaluation, medical knowledge resource
Campbell, et al[72]	1998	Representing thoughts, words, and things in the UMLS	UMLS, knowledge representation,
Campbell, et al[73]	1998	The Unified Medical Language System: toward a collaborative approach for solving terminologic problems	UMLS, terminology
Goldberg, et al[74]	1998	An evaluation of UMLS as a controlled terminology for the Problem List Toolkit	UMLS, evaluation, problem list, mapping

Huff, et al[75]	1998	A proposal for incorporating health level seven (HL7) vocabulary in the UMLS Metathesaurus	UMLS, HL7, vocabulary, data elements, value sets
Humphreys, et al[76]	1998	The Unified Medical Language System: an informatics research collaboration	UMLS, collaborative research, collaborative development
Pisanalli, et al[77]	1998	An ontological analysis of the UMLS Metathesaurus	UMLS Metathesaurus, ontological analysis
Gu, et al[78]	1999	Modeling the UMLS using an OODB	UMLS, modeling, OODB schema
Srinivasan[79]	1999	Exploring the UMLS: a rough sets based theoretical framework	UMLS, vocabularies integration, text retrieval
Gu, et al[80]	2000	Representing the UMLS as an object-oriented database: modeling issues and advantages	Object-oriented database, UMLS comprehension and navigation
Hole, et al[81]	2000	Discovering missed synonymy in a large concept-oriented Metathesaurus	Missing synonymy identification, UMLS
Schulz, et al[82]	2000	Knowledge engineering the UMLS	UMLS, knowledge engineering
Aronson, et al[83]	2001	Effective mapping of biomedical text to the UMLS Metathesaurus: the MetaMap program	MetaMap, mapping, automatic indexing
Bodenreider[84]	2001	An object-oriented model for representing semantic locality in the UMLS	UMLS, semantic locality, object-oriented model, reducing complexity
Bodenreider[85]	2001	Circular hierarchical relationships in the UMLS: etiology, diagnosis, treatment, complications and prevention	Circular hierarchical relationships, UMLS
Cimino[86]	2001	Battling Scylla and Charybdis: the search for redundancy and ambiguity in the 2001 UMLS metathesaurus	UMLS metathesaurus, redundancy, ambiguity
Halper, et al[87]	2001	A metaschema of the UMLS based on a partition of its semantic network	UMLS metaschema, semantic network
McCray, et al[88]	2001	Aggregating UMLS semantic types for reducing conceptual complexity	Aggregation, UMLS, reducing conceptual complexity
Bodenreider, et al[89]	2002	Evaluation of the UMLS as a terminology and knowledge resource for biomedical informatics	LocusLink, Gene Ontology, UMLS evaluation, knowledge resource
Chen, et al[90]	2002	Partitioning the UMLS semantic network	UMLS semantic network, partition

Cornet, et al[91]	2002	Usability of expressive description logics--a case study in UMLS	UMLS, usability, description logicspatient care
Perl, et al[92]	2002	The cohesive metaschema: a higher-level abstraction of the UMLS Semantic Network	Metaschema, the abstraction of the UMLS semantic network
Powell, et al[93]	2002	Tracking meaning over time in the UMLS Metathesaurus	UMLS Metathesaurus, missing synonymy identification
Zhang, et al[94]	2002	Enriching the structure of the UMLS semantic network	UMLS semantic network, enrichment
Kashyap[95]	2003	The UMLS Semantic Network and the Semantic Web	UMLS semantic network, Semantic Web
Mary, et al[96]	2003	Method for automatic management of the semantic network ambiguity in the UMLS: possible application for information retrieval on the Web	Automatic management, semantic network ambiguity
Pratt, et al[97]	2003	A study of biomedical concept identification: MetaMap vs. people	Concept identification by MetaMap
Vipul, et al[98]	2003	Representing the UMLS® semantic network using OWL	UMLS semantic network, OWL
Zhang, et al[99]	2003	Designing metaschemas for the UMLS enriched semantic network	Metaschema, UMLS enriched semantic network,
Bodenreider, et al[100]	2004	Aligning knowledge sources in the UMLS: methods, quantitative results, and applications	UMLS knowledge sources alignment, conceptual and lexical similarity
Bodenreider[101]	2004	The Unified Medical Language System (UMLS): integrating biomedical terminology	UMLS
Davita, et al[102]	2004	Failure analysis of MetaMap Transfer (MMTx)	MMTx, failure analysis, concept extraction
Hole, et al[103]	2004	Achieving "source transparency" in the UMLS Metathesaurus	UMLS sources
Nelson, et al[104]	2004	The MeSH translation maintenance system: structure, interface design, and implementation	Concept-centered vocabulary maintenance system for MeSH
Zhang, et al[105]	2004	An enriched unified medical language system semantic network with a multiple subsumption hierarchy	UMLS enriched semantic network

Mougin, et al[106]	2005	Approaches to eliminating cycles in the UMLS Metathesaurus: naive vs. formal	Circular hierarchical relationships identification
Zhang, et al[107]	2005	A lexical metaschema for the UMLS semantic network	UMLS lexical metaschema, partition
Zhang, et al[108]	2005	An expert study evaluating the UMLS lexical metaschema	UMLS lexical metaschema evaluation
Zhang, et al[109]	2005	Relationship structures and semantic type assignments of the UMLS Enriched Semantic Network	UMLS, enriched semantic network
Fung, et al[110]	2006	Who is using the UMLS and how - insights from the UMLS user annual reports	UMLS use analysis, user annual reports
Patel, et al[111]	2006	Mining cross-terminology links in the UMLS	Link mining approaches, cross-terminology links/paths classification
Robu, et al[112]	2006	An introduction to the Semantic Web for health sciences librarians	Semantic Web, UMLS as an application example
Chen, et al[113]	2007	Analysis of a study of the users, uses, and future agenda of the UMLS	UMLS use
Cohen, et al[114]	2007	Updating the genomic component of the UMLS Semantic Network	UMLS update, genomics, semantic network
Kaiser, et al[115]	2008	MapFace - An Editor for MetaMap Transfer (MMTx)	MMTx, mapping UMLS to medical documents
Cohen, et al[116]	2009	Predication-based semantic indexing: permutations as a means to encode predications in semantic space	SemRep, vector space model, semantic prediction
Huang, et al[117]	2009	Using WordNet synonym substitution to enhance UMLS source integration	Synonym substitution, source integration, UMLS, WordNet
Aronson, et al[118]	2010	An overview of MetaMap: historical perspective and recent advances	MetaMap
Elhanan, et al[119]	2010	Source authenticity in the UMLS--a case study of the Minimal Standard Terminology	UMLS source vocabulary
Xu, et al[120]	2010	A Comprehensive Analysis of Five Million UMLS Metathesaurus Terms Using Eighteen Million MEDLINE Citations	UMLS Metathesaurus Terms analysis
Mohammand, et al[121]	2011	Health: related information structuring for the semantic web	MetaMap Transfer (MMTx) API, upper-level ontology
Chen, et al[122]	2012	Overcoming an obstacle in expanding a UMLS semantic type extent	UMLS expansion, semantic type

Demeke, et al[123]	2012	Enhancing semantic relation quality of UMLS knowledge sources	UMLS knowledge sources, UMLS enhancement
Rosemblat, et al[124]	2013	A methodology for extending domain coverage in SemRep	UMLS, extending domain coverage in SemRep
Ren, et al[125]	2014	Effectively processing medical term queries on the UMLS Metathesaurus by layered dynamic programming	UMLS Metathesaurus query
Gu, et al[126]	2016	Quality Assurance of UMLS Semantic Type Assignments Using SNOMED CT Hierarchies	UMLS quality assurance, UMLS semantic type assignments
Demner-Fushman, et al[127]	2017	MetaMap Lite: an evaluation of a new Java implementation of MetaMap	MetaMap Lite, evaluation
**Coverage of UMLS**			
Cimino[128]	1991	Representation of clinical laboratory terminology in the Unified Medical Language System	UMLS, clinical laboratory terminology
Burgun, et al[129]	1992	Methodology for using the UMLS as a background knowledge for the description of surgical procedures	UMLS, surgical procedures, MAOUSSC modeling
Campbell, et al[130]	1992	The clinical utility of META: an analysis for hypertension	UMLS coverage, ambulatory management of hypertension
Friedman[131]	1992	The UMLS coverage of clinical radiology	UMLS coverage, clinical radiology
Greenes, et al[132]	1992	The findings--diagnosis continuum: implications for image descriptions and clinical databases	UMLS coverage, clinical findings, image descriptions, image findings
Sato, et al[133]	1992	Enhancing the Metathesaurus with clinically relevant concepts: anatomic representations	UMLS coverage, anatomy
Zielstorff, et al[134]	1992	Representation of nursing terminology in the UMLS Metathesaurus: a pilot study	UMLS coverage, nursing
O'keefe, et al[135]	1993	Mendelian inheritance in man: diagnoses in the UMLS	UMLS coverage, human genetic diseases
Humphreys, et al[136]	1996	Planned NLM/AHCPR large-scale vocabulary test: using UMLS technology to determine the extent to which controlled vocabularies cover terminology needed for health care and public health	UMLS coverage evaluation, healthcare, public health

Lange, et al[137]	1996	Representation of everyday clinical nursing language in UMLS and SNOMED	UMLS coverage, nursing
Humphreys, et al[138]	1997	Evaluating the coverage of controlled health data terminologies: report on the results of the NLM/AHCPR large scale vocabulary test	UMLS coverage evaluation
Yu, et al[139]	1999	Representing genomic knowledge in the UMLS semantic network	UMLS, genomic knowledge
Ruggieri, et al[140]	2000	Representation by standard terminologies of health status concepts contained in two health status assessment instruments used in rheumatic disease management	UMLS coverage, rheumatic disease
Barac'h, et al[141]	2002	Dental concepts in the Unified Medical Language System	UMLS coverage, dentistry
Bodenreider, et al[142]	2002	Unsupervised, corpus-based method for extending a biomedical terminology	UMLS coverage, new term identification
Langlotz, et al[143]	2002	The completeness of existing lexicons for representing radiology report information	UMLS coverage, radiology
Mary, et al[144]	2004	MeSH and specialized terminologies: coverage in the field of molecular biology	UMLS coverage, molecular biology
Travers, et al[145]	2006	Unified Medical Language System Coverage of Emergency-medicine Chief Complaints	UMLS coverage, emergency medicine chief complaints
Dorsey, et al[146]	2010	Vocabulary and taxonomy issues when searching lesbian, gay, bisexual and transgender (LGBT) health literature	UMLS coverage, LGBT
Kim, et al[147]	2011	Representation of nursing terminologies in UMLS	Nursing terminology, cross-mapping, UMLS
Kim, et al[148]	2012	Semantic mappings and locality of nursing diagnostic concepts in UMLS	Nursing diagnosis concepts, UMLS, cross-mapping
Manohar, et al[149]	2015	Evaluation of Herbal and Dietary Supplement Resource Term Coverage	UMLS coverage, herbal and dietary supplement
Wang, et al[150]	2016	Term Coverage of Dietary Supplements Ingredients in Product Labels	UMLS coverage, dietary supplements

References

1. Komorowski, H.J., et al., *Browsing and authoring tools for a unified medical language system*, in *User-Oriented Content-Based Text and Image Handling*. 1988, LE CENTRE DE HAUTES ETUDES INTERNATIONALES D'INFORMATIQUE DOCUMENTAIRE: Cambridge, Massachusetts. p. 624–634.
2. Barber, S., et al., *Integrating the UMLS into VNS Retriever.* Proc Annu Symp Comput Appl Med Care, 1992: p. 273-7.

3. Harbourt, A.M., et al., *The ranking algorithm of the Coach browser for the UMLS metathesaurus.* Proc Annu Symp Comput Appl Med Care, 1993: p. 720-4.
4. McCray, A.T. and A. Razi, *The UMLS Knowledge Source server.* Medinfo, 1995. **8 Pt 1**: p. 144-7.
5. Miller, P.L., et al., *Lessons learned from a pilot implementation of the UMLS information sources map.* J Am Med Inform Assoc, 1995. **2**(2): p. 102-15.
6. Tuttle, M.S., et al., *Navigating to knowledge.* Methods Inf Med, 1995. **34**(1-2): p. 214-31.
7. McCray, A.T., et al., *The UMLS Knowledge Source Server: a versatile Internet-based research tool.* Proc AMIA Annu Fall Symp, 1996: p. 164-8.
8. Nadkarni, P.M., *Concept locator: a client-server application for retrieval of UMLS metathesaurus concepts through complex boolean query.* Comput Biomed Res, 1997. **30**(4): p. 323-36.
9. McCray, A.T., et al., *Terminology issues in user access to Web-based medical information.* Proc AMIA Symp, 1999: p. 107-11.
10. Frankewitsch, T. and H.U. Prokosch, *Graphical tool for navigation within the semantic network of the UMLS metathesaurus on a locally installed database.* Stud Health Technol Inform, 2000. **77**: p. 847-51.
11. Wang, J., et al., *Versatile user interface using UMLS Metathesaurus.* Proc AMIA Symp, 2000: p. 888-92.
12. Brandt, C. and P. Nadkarni, *Web-based UMLS concept retrieval by automatic text scanning: a comparison of two methods.* Comput Methods Programs Biomed, 2001. **64**(1): p. 37-43.
13. Bangalore, A., et al., *The UMLS knowledge source server: an object model for delivering UMLS data.* AMIA Annu Symp Proc, 2003: p. 51-5.
14. Browne, A.C., et al., *UMLS language and vocabulary tools.* AMIA Annu Symp Proc, 2003: p. 798.
15. Tao, Y., E.A. Mendonca, and Y.A. Lussier, *A "systematics" tool for medical terminologies.* AMIA Annu Symp Proc, 2003: p. 1028.
16. Mirhaji, P., et al., *A Web Services architecture for UMLS Knowledge Sources.* AMIA Annu Symp Proc, 2005: p. 1055.
17. Bangalore, A., A. Browne, and G. Divita, *UMLSKS SUGGEST: an auto-complete feature for the UMLSKS interface using AJAX.* AMIA Annu Symp Proc, 2006: p. 851.
18. Thorn, K.E., A. Bangalore, and A. Browne, *Plug-and-play UMLS knowledge source server using web services and portlets.* AMIA Annu Symp Proc, 2006: p. 1121.
19. Thorn, K.E., A.K. Bangalore, and A.C. Browne, *The UMLS Knowledge Source Server: an experience in Web 2.0 technologies.* AMIA Annu Symp Proc, 2007: p. 721-5.
20. Aronson, A.R., et al., *Methodology for creating UMLS content views appropriate for biomedical natural language processing.* AMIA Annu Symp Proc, 2008: p. 21-5.
21. Shah, N.H. and M.A. Muse, *UMLS-Query: a perl module for querying the UMLS.* AMIA Annu Symp Proc, 2008: p. 652-6.
22. Demner-Fushman, D., et al., *UMLS content views appropriate for NLP processing of the biomedical literature vs. clinical text.* J Biomed Inform, 2010. **43**(4): p. 587-94.
23. Geller, J., et al., *Rule-based support system for multiple UMLS semantic type assignments.* J Biomed Inform, 2013. **46**(1): p. 97-110.
24. Neuhaus, P., J. Doods, and M. Dugas, *Standardized mappings--a framework to combine different semantic mappers into a standardized web-API.* Stud Health Technol Inform, 2015. **212**: p. 23-6.
25. Rance, B., T. Le, and O. Bodenreider, *Fingerprinting Biomedical Terminologies--Automatic Classification and Visualization of Biomedical Vocabularies through UMLS Semantic Group Profiles.* Stud Health Technol Inform, 2015. **216**: p. 771-5.
26. Cimino, J.J., *Auditing the Unified Medical Language System with semantic methods.* J Am Med Inform Assoc, 1998. **5**(1): p. 41-51.

27. Gu, H.H., et al., *Using the metaschema to audit UMLS classification errors.* Proc AMIA Symp, 2002: p. 310-4.
28. Peng, Y., et al., *Auditing the UMLS for redundant classifications.* Proc AMIA Symp, 2002: p. 612-6.
29. Bodenreider, O. and A.T. McCray, *Exploring semantic groups through visual approaches.* J Biomed Inform, 2003. **36**(6): p. 414-32.
30. Cimino, J.J., H. Min, and Y. Perl, *Consistency across the hierarchies of the UMLS Semantic Network and Metathesaurus.* J Biomed Inform, 2003. **36**(6): p. 450-61.
31. Gu, H., et al., *Auditing concept categorizations in the UMLS.* Artif Intell Med, 2004. **31**(1): p. 29-44.
32. Gu, H.H., et al., *Evaluation of a UMLS Auditing Process of Semantic Type Assignments.* AMIA Annu Symp Proc, 2007: p. 294-8.
33. Chen, Y., et al., *Expanding the extent of a UMLS semantic type via group neighborhood auditing.* J Am Med Inform Assoc, 2009. **16**(5): p. 746-57.
34. Chen, Y., et al., *Structural group auditing of a UMLS semantic type's extent.* J Biomed Inform, 2009. **42**(1): p. 41-52.
35. Chen, Y., et al., *Structural group-based auditing of missing hierarchical relationships in UMLS.* J Biomed Inform, 2009. **42**(3): p. 452-67.
36. Geller, J., et al., *Comparing inconsistent relationship configurations indicating UMLS errors.* AMIA Annu Symp Proc, 2009. **2009**: p. 193-7.
37. Morrey, C.P., et al., *The Neighborhood Auditing Tool: a hybrid interface for auditing the UMLS.* J Biomed Inform, 2009. **42**(3): p. 468-89.
38. Mougin, F., O. Bodenreider, and A. Burgun, *Analyzing polysemous concepts from a clinical perspective: application to auditing concept categorization in the UMLS.* J Biomed Inform, 2009. **42**(3): p. 440-51.
39. Erdogan, H., E. Erdem, and O. Bodenreider, *Exploiting UMLS semantics for checking semantic consistency among UMLS concepts.* Stud Health Technol Inform, 2010. **160**(Pt 1): p. 749-53.
40. Erdoğan, H., O. Bodenreider, and E. Erdem, *Finding semantic inconsistencies in UMLS using answer set programming*, in *Proceedings of the Twenty-Fourth AAAI Conference on Artificial Intelligence*. 2010, AAAI Press: Atlanta, Georgia. p. 1927–1928.
41. Huang, K.C., et al., *Auditing SNOMED Integration into the UMLS for Duplicate Concepts.* AMIA Annu Symp Proc, 2010. **2010**: p. 321-5.
42. Rudniy, A., J. Geller, and M. Song, *Shortest Path Edit Distance for Enhancing UMLS Integration and Audit.* AMIA Annu Symp Proc, 2010. **2010**: p. 697-701.
43. Halper, M., et al., *Auditing hierarchical cycles to locate other inconsistencies in the UMLS.* AMIA Annu Symp Proc, 2011. **2011**: p. 529-36.
44. Morrey, C.P., et al., *Resolution of redundant semantic type assignments for organic chemicals in the UMLS.* Artif Intell Med, 2011. **52**(3): p. 141-51.
45. Ochs, C., J. Geller, and Y. Perl, *A RELATIONSHIP-CENTRIC HYBRID INTERFACE FOR BROWSING AND AUDITING THE UMLS.* J. Integr. Des. Process Sci., 2011. **15**(4): p. 3–25.
46. Gu, H.H., et al., *A study of terminology auditors' performance for UMLS semantic type assignments.* J Biomed Inform, 2012. **45**(6): p. 1042-8.
47. Wei, D., M. Halper, and G. Elhanan, *Using SNOMED semantic concept groupings to enhance semantic-type assignment consistency in the UMLS*, in *Proceedings of the 2nd ACM SIGHIT International Health Informatics Symposium*. 2012, Association for Computing Machinery: Miami, Florida, USA. p. 825–830.
48. Mougin, F. and N. Grabar, *Auditing the multiply-related concepts within the UMLS.* J Am Med Inform Assoc, 2014. **21**(e2): p. e185-93.

49. Gu, H., et al., *Validating UMLS Semantic Type Assignments Using SNOMED CT Semantic Tags.* Methods Inf Med, 2018. **57**(1): p. 43-53.
50. Komorowski, J. and Å. Akademi, *Knowledge modeling for the unified medical language system*, in *Information modelling and knowledge bases*. 1990, IOS Press. p. 313–317.
51. Humphreys, B.L., D.A. Lindberg, and W.T. Hole, *Assessing and enhancing the value of the UMLS Knowledge Sources.* Proc Annu Symp Comput Appl Med Care, 1991: p. 78-82.
52. Nelson, S.J., et al., *From meaning to term: semantic locality in the UMLS Metathesaurus.* Proc Annu Symp Comput Appl Med Care, 1991: p. 209-13.
53. Tuttle, M.S., et al., *Adding your terms and relationships to the UMLS Metathesaurus.* Proc Annu Symp Comput Appl Med Care, 1991: p. 219-23.
54. Yang, Y. and C.G. Chute, *A schematic analysis of the Unified Medical Language System.* Proc Annu Symp Comput Appl Med Care, 1991: p. 204-8.
55. Lindberg, D.A.B. and B.L. Humphreys, *The unified medical language system (UMLS) and computer-based patient records*, in *Aspects of the computer-based patient record*. 1992, Springer-Verlag. p. 165–175.
56. Masys, D.R., *An evaluation of the source selection elements of the prototype UMLS Information Sources Map.* Proc Annu Symp Comput Appl Med Care, 1992: p. 295-8.
57. Nelson, S.J., et al., *The semantic structure of the UMLS Metathesaurus.* Proc Annu Symp Comput Appl Med Care, 1992: p. 649-53.
58. Sperzel, W.D., et al., *The Meta-1.2 engine: a refined strategy for linking biomedical vocabularies.* Proc Annu Symp Comput Appl Med Care, 1992: p. 304-8.
59. Tuttle, M.S., et al., *The homogenization of the Metathesaurus schema and distribution format.* Proc Annu Symp Comput Appl Med Care, 1992: p. 299-303.
60. Clyman, J.I., et al., *Using a network menu and the UMLS Information Sources Map to facilitate access to online reference materials.* Bull Med Libr Assoc, 1993. **81**(2): p. 207-16.
61. Humphreys, B.L. and D.A. Lindberg, *The UMLS project: making the conceptual connection between users and the information they need.* Bull Med Libr Assoc, 1993. **81**(2): p. 170-7.
62. Humphreys, B.L. and P.L. Schuyler, *The unified medical language system: moving beyond the vocabulary of bibliographic retrieval*, in *High performance medical libraries: Advances in information management for the virtual era*. 1993, Meckler Corporation. p. 31–44.
63. Lindberg, D.A., B.L. Humphreys, and A.T. McCray, *The Unified Medical Language System.* Methods Inf Med, 1993. **32**(4): p. 281-91.
64. McCray, A.T., *Representing biomedical knowledge in the UMLS semantic network*, in *High performance medical libraries: Advances in information management for the virtual era*. 1993, Meckler Corporation. p. 45–55.
65. Schuyler, P.L., et al., *The UMLS Metathesaurus: representing different views of biomedical concepts.* Bull Med Libr Assoc, 1993. **81**(2): p. 217-22.
66. Tuttle, M.S. and S.J. Nelson, *The role of the UMLS in 'storing' and 'sharing' across systems.* Int J Biomed Comput, 1994. **34**(1-4): p. 207-37.
67. Burgun, A., et al., *Knowledge acquisition from the UMLS sources: application to the description of surgical procedures.* Medinfo, 1995. **8 Pt 1**: p. 75-9.
68. Joubert, M., et al., *A conceptual graphs modeling of UMLS components.* Medinfo, 1995. **8 Pt 1**: p. 90-4.
69. McCray, A.T. and G. Divita, *ASN.1: defining a grammar for the UMLS knowledge sources.* Proc Annu Symp Comput Appl Med Care, 1995: p. 868-72.
70. McCray, A.T. and S.J. Nelson, *The representation of meaning in the UMLS.* Methods Inf Med, 1995. **34**(1-2): p. 193-201.

71. Bodenreider, O., et al., *Evaluation of the Unified Medical Language System as a medical knowledge source.* J Am Med Inform Assoc, 1998. **5**(1): p. 76-87.
72. Campbell, K.E., et al., *Representing thoughts, words, and things in the UMLS.* J Am Med Inform Assoc, 1998. **5**(5): p. 421-31.
73. Campbell, K.E., D.E. Oliver, and E.H. Shortliffe, *The Unified Medical Language System: toward a collaborative approach for solving terminologic problems.* J Am Med Inform Assoc, 1998. **5**(1): p. 12-6.
74. Goldberg, H., et al., *An evaluation of UMLS as a controlled terminology for the Problem List Toolkit.* Stud Health Technol Inform, 1998. **52 Pt 1**: p. 609-12.
75. Huff, S.M., et al., *A proposal for incorporating health level seven (HL7) vocabulary in the UMLS Metathesaurus.* Proc AMIA Symp, 1998: p. 800-4.
76. Humphreys, B.L., et al., *The Unified Medical Language System: an informatics research collaboration.* J Am Med Inform Assoc, 1998. **5**(1): p. 1-11.
77. Pisanelli, D.M., A. Gangemi, and G. Steve, *An ontological analysis of the UMLS Metathesaurus.* Proc AMIA Symp, 1998: p. 810-4.
78. Gu, H., et al., *Modeling the UMLS using an OODB.* Proc AMIA Symp, 1999: p. 82-6.
79. Srinivasan, P., *Exploring the UMLS: a rough sets based theoretical framework.* Proc AMIA Symp, 1999: p. 156-60.
80. Gu, H., et al., *Representing the UMLS as an object-oriented database: modeling issues and advantages.* J Am Med Inform Assoc, 2000. **7**(1): p. 66-80.
81. Hole, W.T. and S. Srinivasan, *Discovering missed synonymy in a large concept-oriented Metathesaurus.* Proc AMIA Symp, 2000: p. 354-8.
82. Schulz, S., M. Romacker, and U. Hahn, *Knowledge engineering the UMLS.* Stud Health Technol Inform, 2000. **77**: p. 701-5.
83. Aronson, A.R., *Effective mapping of biomedical text to the UMLS Metathesaurus: the MetaMap program.* Proc AMIA Symp, 2001: p. 17-21.
84. Bodenreider, O., *An object-oriented model for representing semantic locality in the UMLS.* Stud Health Technol Inform, 2001. **84**(Pt 1): p. 161-5.
85. Bodenreider, O., *Circular hierarchical relationships in the UMLS: etiology, diagnosis, treatment, complications and prevention.* Proc AMIA Symp, 2001: p. 57-61.
86. Cimino, J.J., *Battling Scylla and Charybdis: the search for redundancy and ambiguity in the 2001 UMLS metathesaurus.* Proc AMIA Symp, 2001: p. 120-4.
87. Halper, M.H., et al., *A metaschema of the UMLS based on a partition of its semantic network.* Proc AMIA Symp, 2001: p. 234-8.
88. McCray, A.T., A. Burgun, and O. Bodenreider, *Aggregating UMLS semantic types for reducing conceptual complexity.* Stud Health Technol Inform, 2001. **84**(Pt 1): p. 216-20.
89. Bodenreider, O., J.A. Mitchell, and A.T. McCray, *Evaluation of the UMLS as a terminology and knowledge resource for biomedical informatics.* Proc AMIA Symp, 2002: p. 61-5.
90. Chen, Z., et al., *Partitioning the UMLS semantic network.* IEEE Trans Inf Technol Biomed, 2002. **6**(2): p. 102-8.
91. Cornet, R. and A. Abu-Hanna, *Usability of expressive description logics--a case study in UMLS.* Proc AMIA Symp, 2002: p. 180-4.
92. Perl, Y., et al., *The cohesive metaschema: a higher-level abstraction of the UMLS Semantic Network.* J Biomed Inform, 2002. **35**(3): p. 194-212.
93. Powell, T., et al., *Tracking meaning over time in the UMLS Metathesaurus.* Proc AMIA Symp, 2002: p. 622-6.
94. Zhang, L., et al., *Enriching the structure of the UMLS semantic network.* Proc AMIA Symp, 2002: p. 939-43.

95. Kashyap, V., *The UMLS Semantic Network and the Semantic Web.* AMIA Annu Symp Proc, 2003: p. 351-5.
96. Mary, V., et al., *Method for automatic management of the semantic network ambiguity in the UMLS: possible application for information retrieval on the Web.* Stud Health Technol Inform, 2003. **95**: p. 475-9.
97. Pratt, W. and M. Yetisgen-Yildiz, *A study of biomedical concept identification: MetaMap vs. people.* AMIA Annu Symp Proc, 2003: p. 529-33.
98. Kashyap, V. and A. Borgida, *Representing the UMLS® semantic network using OWL*, in *Proceedings of the Second International Conference on Semantic Web Conference*. 2003, Springer-Verlag: Sanibel Island, FL. p. 1–16.
99. Zhang, L., et al., *Designing metaschemas for the UMLS enriched semantic network.* J Biomed Inform, 2003. **36**(6): p. 433-49.
100. Bodenreider, O. and A. Burgun, *Aligning knowledge sources in the UMLS: methods, quantitative results, and applications.* Stud Health Technol Inform, 2004. **107**(Pt 1): p. 327-31.
101. Bodenreider, O., *The Unified Medical Language System (UMLS): integrating biomedical terminology.* Nucleic Acids Res, 2004. **32**(Database issue): p. D267-70.
102. Divita, G., T. Tse, and L. Roth, *Failure analysis of MetaMap Transfer (MMTx).* Stud Health Technol Inform, 2004. **107**(Pt 2): p. 763-7.
103. Hole, W.T., et al., *Achieving "source transparency" in the UMLS Metathesaurus.* Stud Health Technol Inform, 2004. **107**(Pt 1): p. 371-5.
104. Nelson, S.J., et al., *The MeSH translation maintenance system: structure, interface design, and implementation.* Stud Health Technol Inform, 2004. **107**(Pt 1): p. 67-9.
105. Zhang, L., et al., *An enriched unified medical language system semantic network with a multiple subsumption hierarchy.* J Am Med Inform Assoc, 2004. **11**(3): p. 195-206.
106. Mougin, F. and O. Bodenreider, *Approaches to eliminating cycles in the UMLS Metathesaurus: naive vs. formal.* AMIA Annu Symp Proc, 2005: p. 550-4.
107. Zhang, L., et al., *A lexical metaschema for the UMLS semantic network.* Artif Intell Med, 2005. **33**(1): p. 41-59.
108. Zhang, L., et al., *An expert study evaluating the UMLS lexical metaschema.* Artif Intell Med, 2005. **34**(3): p. 219-33.
109. Zhang, L., et al., *Relationship structures and semantic type assignments of the UMLS Enriched Semantic Network.* J Am Med Inform Assoc, 2005. **12**(6): p. 657-66.
110. Fung, K.W., W.T. Hole, and S. Srinivasan, *Who is using the UMLS and how - insights from the UMLS user annual reports.* AMIA Annu Symp Proc, 2006: p. 274-8.
111. Patel, C.O. and J.J. Cimino, *Mining cross-terminology links in the UMLS.* AMIA Annu Symp Proc, 2006: p. 624-8.
112. Robu, I., V. Robu, and B. Thirion, *An introduction to the Semantic Web for health sciences librarians*.* Journal of the Medical Library Association, 2006. **94**(2): p. 198-205.
113. Chen, Y., et al., *Analysis of a study of the users, uses, and future agenda of the UMLS.* J Am Med Inform Assoc, 2007. **14**(2): p. 221-31.
114. Cohen, B., Y. Chen, and Y. Perl, *Updating the genomic component of the UMLS Semantic Network.* AMIA Annu Symp Proc, 2007: p. 150-4.
115. Kaiser, K., T. Gschwandtner, and P. Martini, *MapFace - An Editor for MetaMap Transfer (MMTx)*, in *Proceedings of the 2008 21st IEEE International Symposium on Computer-Based Medical Systems*. 2008, IEEE Computer Society. p. 150–152.
116. Cohen, T., R.W. Schvaneveldt, and T.C. Rindflesch, *Predication-based semantic indexing: permutations as a means to encode predications in semantic space.* AMIA Annu Symp Proc, 2009. **2009**: p. 114-8.

117. Huang, K.C., et al., *Using WordNet synonym substitution to enhance UMLS source integration.* Artif Intell Med, 2009. **46**(2): p. 97-109.
118. Aronson, A.R. and F.M. Lang, *An overview of MetaMap: historical perspective and recent advances.* J Am Med Inform Assoc, 2010. **17**(3): p. 229-36.
119. Elhanan, G., K.C. Huang, and Y. Perl, *Source authenticity in the UMLS--a case study of the Minimal Standard Terminology.* J Biomed Inform, 2010. **43**(6): p. 988-97.
120. Xu, R., M.A. Musen, and N.H. Shah, *A Comprehensive Analysis of Five Million UMLS Metathesaurus Terms Using Eighteen Million MEDLINE Citations.* AMIA Annu Symp Proc, 2010. **2010**: p. 907-11.
121. Eljinini, M.A.H., *Health: related information structuring for the semantic web*, in *Proceedings of the 2011 International Conference on Intelligent Semantic Web-Services and Applications*. 2011, Association for Computing Machinery: Amman, Jordan. p. Article 6.
122. Chen, Y., et al., *Overcoming an obstacle in expanding a UMLS semantic type extent.* J Biomed Inform, 2012. **45**(1): p. 61-70.
123. Ayele, D., et al., *Enhancing semantic relation quality of UMLS knowledge sources*, in *Proceedings of the International Conference on Management of Emergent Digital EcoSystems*. 2012, Association for Computing Machinery: Addis Ababa, Ethiopia. p. 59–66.
124. Rosemblat, G., et al., *A methodology for extending domain coverage in SemRep.* J Biomed Inform, 2013. **46**(6): p. 1099-107.
125. Ren, K., et al., *Effectively processing medical term queries on the UMLS Metathesaurus by layered dynamic programming.* BMC Med Genomics, 2014. **7 Suppl 1**: p. S11.
126. Gu, H., et al., *Quality Assurance of UMLS Semantic Type Assignments Using SNOMED CT Hierarchies.* Methods Inf Med, 2016. **55**(2): p. 158-65.
127. Demner-Fushman, D., W.J. Rogers, and A.R. Aronson, *MetaMap Lite: an evaluation of a new Java implementation of MetaMap.* J Am Med Inform Assoc, 2017. **24**(4): p. 841-844.
128. Cimino, J.J., *Representation of clinical laboratory terminology in the Unified Medical Language System.* Proc Annu Symp Comput Appl Med Care, 1991: p. 199-203.
129. Burgun, A., et al., *Methodology for using the UMLS as a background knowledge for the description of surgical procedures.* Int J Biomed Comput, 1996. **43**(3): p. 189-202.
130. Campbell, J.R., G.A. Kallenberg, and R.C. Sherrick, *The clinical utility of META: an analysis for hypertension.* Proc Annu Symp Comput Appl Med Care, 1992: p. 397-401.
131. Friedman, C., *The UMLS coverage of clinical radiology.* Proc Annu Symp Comput Appl Med Care, 1992: p. 309-13.
132. Greenes, R.A., et al., *The findings--diagnosis continuum: implications for image descriptions and clinical databases.* Proc Annu Symp Comput Appl Med Care, 1992: p. 383-7.
133. Sato, L., et al., *Enhancing the Metathesaurus with clinically relevant concepts: anatomic representations.* Proc Annu Symp Comput Appl Med Care, 1992: p. 388-91.
134. Zielstorff, R.D., et al., *Representation of nursing terminology in the UMLS Metathesaurus: a pilot study.* Proc Annu Symp Comput Appl Med Care, 1992: p. 392-6.
135. O'Keefe, K.M., M. Sievert, and J.A. Mitchell, *Mendelian inheritance in man: diagnoses in the UMLS.* Proc Annu Symp Comput Appl Med Care, 1993: p. 735-9.
136. Humphreys, B.L., et al., *Planned NLM/AHCPR large-scale vocabulary test: using UMLS technology to determine the extent to which controlled vocabularies cover terminology needed for health care and public health.* J Am Med Inform Assoc, 1996. **3**(4): p. 281-7.
137. Lange, L.L., *Representation of everyday clinical nursing language in UMLS and SNOMED.* Proc AMIA Annu Fall Symp, 1996: p. 140-4.

138. Humphreys, B.L., A.T. McCray, and M.L. Cheh, *Evaluating the coverage of controlled health data terminologies: report on the results of the NLM/AHCPR large scale vocabulary test.* J Am Med Inform Assoc, 1997. **4**(6): p. 484-500.
139. Yu, H., et al., *Representing genomic knowledge in the UMLS semantic network.* Proc AMIA Symp, 1999: p. 181-5.
140. Ruggieri, A.P., P. Elkin, and C.G. Chute, *Representation by standard terminologies of health status concepts contained in two health status assessment instruments used in rheumatic disease management.* Proc AMIA Symp, 2000: p. 734-8.
141. Barac'h, V. and T.K. Schleyer, *Dental concepts in the Unified Medical Language System.* Quintessence Int, 2002. **33**(1): p. 69-74.
142. Bodenreider, O., T.C. Rindflesch, and A. Burgun, *Unsupervised, corpus-based method for extending a biomedical terminology*, in *Proceedings of the ACL-02 workshop on Natural language processing in the biomedical domain - Volume 3*. 2002, Association for Computational Linguistics: Phildadelphia, Pennsylvania. p. 53–60.
143. Langlotz, C.P. and S.A. Caldwell, *The completeness of existing lexicons for representing radiology report information.* J Digit Imaging, 2002. **15 Suppl 1**: p. 201-5.
144. Mary, V., G. Marquet, and P. Le Beux, *MeSH and specialized terminologies: coverage in the field of molecular biology.* Stud Health Technol Inform, 2004. **107**(Pt 1): p. 530-4.
145. Travers, D.A. and S.W. Haas, *Unified Medical Language System Coverage of Emergency-medicine Chief Complaints.* Academic Emergency Medicine, 2006. **13**(12): p. 1319.
146. Dorsey, M.J. and E. Detlefsen, *Vocabulary and taxonomy issues when searching lesbian, gay, bisexual and transgender (LGBT) health literature*, in *Proceedings of the 2010 International Conference on Dublin Core and Metadata Applications*. 2010, Dublin Core Metadata Initiative: Pittsburgh, Pennsylvania. p. 180–181.
147. Kim, T.Y., et al., *Representation of nursing terminologies in UMLS.* AMIA Annu Symp Proc, 2011. **2011**: p. 709-14.
148. Kim, T.Y., A. Coenen, and N. Hardiker, *Semantic mappings and locality of nursing diagnostic concepts in UMLS.* J Biomed Inform, 2012. **45**(1): p. 93-100.
149. Manohar, N., et al., *Evaluation of Herbal and Dietary Supplement Resource Term Coverage.* Stud Health Technol Inform, 2015. **216**: p. 785-9.
150. Wang, Y., T.J. Adam, and R. Zhang, *Term Coverage of Dietary Supplements Ingredients in Product Labels.* AMIA Annu Symp Proc, 2016. **2016**: p. 2053-2061.
