# Supplementary material for: The Unified Medical Language System at 30 Years and How It Is Used and Published: Systematic Review and Content Analysis
Source: JMIR Med Inform. 2021 Aug 27;9(8):e20675. doi: 10.2196/20675 (PMC8433943; doi:10.2196/20675)
Supplement: Multimedia Appendix 13 [file medinform_v9i8e20675_app13.pdf]

Multimedia Appendix 13. Unified Medical Language System publications for purposes other than the themes noted above.

Author	Publication year	Title	What was UMLS used for?
**Auditing**			
Goldberg, et al[1]	1998	Validation of clinical problems using a UMLS-based semantic parser	Validation of clinical problems, enrichment of UMLS lexicon, UMLS-based semantic parser
Nawab, et al[2]	2008	Comparing Medline citations using modified N-grams	Identification of duplicate MEDLINE citations
Mahato, et al[3]	2019	A Method to Detect Inconsistent Annotations in a Medical Document using UMLS	Information retrieval, annotated corpus, inconsistency, identification of UMLS concepts
**Consumer health**			
Leroy, et al[4]	2010	Perils of providing visual health information overviews for consumers with low health literacy or high stress	Visualization, low health literacy or high stress, consumer health
Leroy, et al[5]	2012	Improving perceived and actual text difficulty for health information consumers using semi-automated methods	Simplification of medical text, term familiarity, Easier alternatives from UMLS
Leroy, et al[6]	2013	User evaluation of the effects of a text simplification algorithm using term familiarity on perception, understanding, learning, and information retention	Text simplification, term familiarity, text analysis
Qenam, et al[7]	2017	Text Simplification Using Consumer Health Vocabulary to Generate Patient-Centered Radiology Reporting: Translation and Evaluation	Consumer Health Vocabulary, Radiology Reporting, lexical simplification
**Integrated system/data**			
Sperzel, et al[8]	1991	Biomedical database inter-connectivity: an experiment linking MIM, GENBANK, and META-1 via MEDLINE	Linking MIM, GENBANK, META-1 via MEDLINE, inter-connectivity
Chute, et al[9]	1994	Medical data and knowledge management by integrated medical workstations: summary and recommendations	Clinical data management, knowledge management, integrated system

Detmer, et al[10]	1997	MedWeaver: integrating decision support, literature searching, and Web exploration using the UMLS Metathesaurus	MedWeaver, decision support, literature search, UMLS
Volot, et al[11]	1997	A UMLS-based method for integrating information databases into an Intranet	Integration of information databases, UMLS-based methods
Joubert, et al[12]	1998	ARIANE: integration of information databases within a hospital intranet	ARIANE, access and query heterogeneous information databases, UMLS
Joubert, et al[13]	1998	Conceptual integration of information databases into an Intranet	ARIANE, access and query information databases, UMLS, conceptual integration
Aymard, et al[14]	2000	Mediation services with health information sources	ARIANE, mediation services, UMLS knowledge sources
Joubert, et al[15]	2001	ARIANE: a mediation framework with health information sources	ARIANE, mediation framework, UMLS, access heterogeneous information sources
Chun, et al[16]	2002	Social health data integration using semantic Web	Link scattered health-related data, RDF
Cantor, et al[17]	2003	Putting data integration into practice: using biomedical terminologies to add structure to existing data sources	Data integration, UMLS, SNOMED CT, semantic relationship, logical relationship
Nardon, et al[18]	2004	Knowledge sharing and information integration in healthcare using ontologies and deductive databases	Ontology, knowledge sharing, information integration
Wang, et al[19]	2004	Automating terminological networks to link heterogeneous biomedical databases	Automated mapping, linking heterogeneous databases
Berlanga, et al[20]	2008	Medical Data Integration and the Semantic Annotation of Medical Protocols	Integration of heterogeneous biomedical information, decision support system
Yang, et al[21]	2008	An integrated database-pipeline system for studying single nucleotide polymorphisms and diseases	SNP and disease, disease and genetic variations, unite disease names and gene names, UMLS
Yamamoto, et al[22]	2011	Building linked open data using approximate string matching methods and domain specific resources	Linked open data set, abbreviation-long form pairs, UMLS as standard term source
Neveol, et al[23]	2012	Linking multiple disease-related resources through UMLS	Multiple disease-related resources, UMLS

Mazouz, et al[24]	2017	Towards a system for integrating heterogeneous health records	Integration of heterogeneous health records, exchange and share patient information
**Other research use**			
Berman[25]	2003	Concept-match medical data scrubbing. How pathology text can be used in research	Concept match, scrubbing, pathology text, deidentification, UMLS
Zhang, et al[26]	2007	Building user research interest profiles through a MeSH indexer	Research profiles by MeSH terms, MeSH indexer
Boyd, et al[27]	2018	Physician nurse care: A new use of UMLS to measure professional contribution: Are we talking about the same patient a new graph matching algorithm?	UMLS for contribution measurement, graph algorithm

References

1. Goldberg, H.S., et al., *Validation of clinical problems using a UMLS-based semantic parser.* Proc AMIA Symp, 1998: p. 805-9.
2. Nawab, R.M., M. Stevenson, and P. Clough, *Comparing Medline citations using modified N-grams.* J Am Med Inform Assoc, 2014. **21**(1): p. 105-10.
3. Mahato, D., et al., *A Method to Detect Inconsistent Annotations in a Medical Document using UMLS*, in *Proceedings of the 11th Forum for Information Retrieval Evaluation*. 2019, Association for Computing Machinery: Kolkata, India. p. 47–51.
4. Leroy, G. and T. Miller, *Perils of providing visual health information overviews for consumers with low health literacy or high stress.* J Am Med Inform Assoc, 2010. **17**(2): p. 220-3.
5. Leroy, G., et al., *Improving perceived and actual text difficulty for health information consumers using semi-automated methods.* AMIA Annu Symp Proc, 2012. **2012**: p. 522-31.
6. Leroy, G., et al., *User evaluation of the effects of a text simplification algorithm using term familiarity on perception, understanding, learning, and information retention.* J Med Internet Res, 2013. **15**(7): p. e144.
7. Qenam, B., et al., *Text Simplification Using Consumer Health Vocabulary to Generate Patient-Centered Radiology Reporting: Translation and Evaluation.* J Med Internet Res, 2017. **19**(12): p. e417.
8. Sperzel, W.D., et al., *Biomedical database inter-connectivity: an experiment linking MIM, GENBANK, and META-1 via MEDLINE.* Proc Annu Symp Comput Appl Med Care, 1991: p. 190-3.
9. Chute, C.G., B. Cesnik, and J.H. van Bemmel, *Medical data and knowledge management by integrated medical workstations: summary and recommendations.* Int J Biomed Comput, 1994. **34**(1-4): p. 175-83.
10. Detmer, W.M., G.O. Barnett, and W.R. Hersh, *MedWeaver: integrating decision support, literature searching, and Web exploration using the UMLS Metathesaurus.* Proc AMIA Annu Fall Symp, 1997: p. 490-4.
11. Volot, F., et al., *A UMLS-based method for integrating information databases into an Intranet.* Proc AMIA Annu Fall Symp, 1997: p. 495-9.

12. Joubert, M., et al., *ARIANE: integration of information databases within a hospital intranet.* Int J Med Inform, 1998. **49**(3): p. 297-309.
13. Joubert, M., et al., *Conceptual integration of information databases into an Intranet.* Stud Health Technol Inform, 1998. **52 Pt 1**: p. 161-5.
14. Aymard, S., et al., *Mediation services with health information sources.* Proc AMIA Symp, 2000: p. 37-41.
15. Joubert, M., et al., *ARIANE: a mediation framework with health information sources.* Stud Health Technol Inform, 2001. **84**(Pt 1): p. 343-7.
16. Chun, S.A. and B. MacKellar, *Social health data integration using semantic Web*, in *Proceedings of the 27th Annual ACM Symposium on Applied Computing*. 2012, Association for Computing Machinery: Trento, Italy. p. 392–397.
17. Cantor, M.N. and Y.A. Lussier, *Putting data integration into practice: using biomedical terminologies to add structure to existing data sources.* AMIA Annu Symp Proc, 2003: p. 125-9.
18. Nardon, F.B. and L.A. Moura, *Knowledge sharing and information integration in healthcare using ontologies and deductive databases.* Stud Health Technol Inform, 2004. **107**(Pt 1): p. 62-6.
19. Wang, X., et al., *Automating terminological networks to link heterogeneous biomedical databases.* Stud Health Technol Inform, 2004. **107**(Pt 1): p. 555-9.
20. Berlanga, R., et al., *Medical Data Integration and the Semantic Annotation of Medical Protocols*, in *Proceedings of the 2008 21st IEEE International Symposium on Computer-Based Medical Systems*. 2008, IEEE Computer Society. p. 644–649.
21. Yang, J.O., et al., *An integrated database-pipeline system for studying single nucleotide polymorphisms and diseases.* BMC Bioinformatics, 2008. **9 Suppl 12**: p. S19.
22. Yamamoto, Y., A. Yamaguchi, and A. Yonezawa, *Building linked open data using approximate string matching methods and domain specific resources*, in *Proceedings of the 4th International Workshop on Semantic Web Applications and Tools for the Life Sciences*. 2011, Association for Computing Machinery: London, United Kingdom. p. 121–122.
23. Névéol, A., J. Li, and Z. Lu, *Linking multiple disease-related resources through UMLS*, in *Proceedings of the 2nd ACM SIGHIT International Health Informatics Symposium*. 2012, Association for Computing Machinery: Miami, Florida, USA. p. 767–772.
24. Mazouz, S., O.M.C. Malki, and A. Elloub, *Towards a system for integrating heterogeneous health records*, in *Proceedings of the Mediterranean Symposium on Smart City Application*. 2017, Association for Computing Machinery: Tangier, Morocco. p. Article 1.
25. Berman, J.J., *Concept-match medical data scrubbing: How pathology text can be used in research.* Archives of Pathology & Laboratory Medicine, 2003. **127**(6): p. 680-6.
26. Zhang, D., et al., *Building user research interest profiles through a MeSH indexer.* AMIA Annu Symp Proc, 2007: p. 1171.
27. Boyd, A.D., et al., *Physician nurse care: A new use of UMLS to measure professional contribution: Are we talking about the same patient a new graph matching algorithm?* Int J Med Inform, 2018. **113**: p. 63-71.
